# ALKBH5‐Mediated M^6^A Demethylation of G3BP1 Attenuates Ferroptosis Via Cytoplasmic Retention of YBX1/p53 in Diabetic Myocardial Ischemia‐Reperfusion Injury

**DOI:** 10.1002/advs.202507254

**Published:** 2025-06-23

**Authors:** Wenyuan Li, Wei Li, Yan Leng, Heng Xu, Zhongyuan Xia, Yao Wang

**Affiliations:** ^1^ Department of Anesthesiology Renmin Hospital of Wuhan University Wuhan Hubei 430060 China; ^2^ Department of Infectious Diseases Renmin Hospital of Wuhan University Wuhan Hubei 430060 China

**Keywords:** ALKBH5, diabetes, ferroptosis, G3BP1, myocardial ischemia‒reperfusion injury

## Abstract

The overexpression of ALKBH5 alleviates damage to cardiomyocytes and tissues, decreases the expression of SLC7A11, and inhibits the expression of p53^N/T^ and ferroptosis. ALKBH5 induces ferroptosis in the HH/R model by mutating the 3′‐UTR of G3BP1 mRNA and m^6^A sites at positions 142 and 173. Mutations of sites 142 and 173 only partially affect the levels of p53^N/T^, SLC7A11 and ferroptosis. G3BP1, YBX1 and p53 protein can bind to each other, and p53 can inhibit the expression of SLC7A11 via nuclear binding to the promoter region of SLC7A11 mRNA. The p53 and YBX1 proteins can synergistically enter the nucleus to aggravate cell damage, while G3BP1 is expressed in the cytoplasm and inhibits p53 binding to SLC7A11 by blocking the entry of the p53 and YBX1 proteins into the nucleus. ALKBH5 promotes G3BP1 expression through m^6^A methylation at m^6^A sites 142 and 173. Subsequently, G3BP1, YBX1, and p53 interact with each other, the amount of synergistic nuclear translocation of YBX1 and p53 is reduced, and the level of p53 nuclear translocation that inhibiting SLC7A11 transcription is decreased, thereby inhibits cardiomyocyte ferroptosis and reducing myocardial tissue damage during myocardial IRI in diabetes.

## Graphical abstract

ALKBH5 promoted G3BP1 expression via m⁶A methylation at sites 142/173. G3BP1 interacts with YBX1 and p53, reducing their nuclear translocation and decreasing p53‐mediated SLC7A11 repression. This inhibites cardiomyocyte ferroptosis and mitigates myocardial damage during diabetic ischemia‐reperfusion injury.

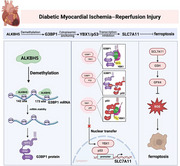

## 1 Introduction

Diabetes mellitus (DM) is a highly prevalent metabolic disease characterized by chronic hyperglycemia and multiple life‐threatening complications. Diabetic ischemic heart disease has become the leading cause of death in diabetic patients.^[^ ^1^ ^]^ During reperfusion of the ischemic myocardium, diabetic patients can produce more reactive oxygen species (ROS), aggravating cell inflammation, necrosis, apoptosis, etc., resulting in more severe ischemia‒reperfusion injury (IRI) and a worse prognosis.^[^ ^2^ ^]^ Patients with diabetes and coronary heart disease often suffer from myocardial infarction without typical pain symptoms. When they seek medical treatment, large areas of infarction have already appeared. After blood flow is restored, they are prone to secondary severe reperfusion injury, which further exacerbates their condition. Therefore, this important issue needs to be urgently addressed in related disciplines to clarify the pathogenesis of DM‐IRI (DIR) from a new perspective, find more effective protective measures and intervention opportunities, and improve patient prognosis.

As one of the most abundant and common internal RNA modifications, m^6^A methylation regulates gene expression at the post‐transcriptional level by affecting RNA translation, stability, splicing and transport processes and affecting cell metabolism, proliferation, differentiation, and other biological processes.^[^ ^3^, ^4^, ^5^ ^]^ The regulation of m^6^A methylation jointly involves methyltransferases (writers), demethylases (erasers), and methylation reader proteins (readers). Among them, ALKBH5 is a key enzyme for RNA demethylation. m^6^A affects various biological processes in cells by regulating the m^6^A modification of target genes. Studies have shown that ALKBH5 plays important roles in cardiomyocyte proliferation and cardiac regeneration,^[^ ^6^ ^]^ ischemic diseases,^[^ ^7^ ^]^ blood diseases,^[^ ^8^ ^]^ and tumors.^[^ ^9^ ^]^ Studies have also shown that m^6^A modification is closely related to the hypoxia‒reoxygenation process in cardiomyocytes,^[^ ^10^ ^]^ but the specific mechanism by which ALKBH5 is involved and how it affects diabetic myocardial ischemia‒reperfusion injury are still unknown.

Ferroptosis is a type of regulated cell death discovered in 2012 that relies on iron and reactive oxygen species (ROS). Its main manifestations are the accumulation of lipid reactive oxygen species and iron, significant shrinkage of mitochondria, and increased membrane density.^[^ ^11^ ^]^ By regulating intracellular oxidative stress, iron homeostasis, iron metabolism and other related processes, m^6^A methylation may play a regulatory role in cellular ferroptosis and tumor progression. Recent studies have suggested that the m^6^A methylase METTL3 can participate in the process of septic lung injury by regulating ferroptosis.^[^ ^12^ ^]^ FTO‐mediated m^6^A modification plays a key role in alleviating IRI in elderly livers by regulating the key ferroptosis molecule ACSL4.^[^ ^13^ ^]^ METTL3‐mediated SLC7A11 m^6^A modification induces hepatoblastoma resistance to ferroptosis.^[^ ^14^ ^]^ Our prior work identified GTPase‐activating protein SH3‐binding protein 1 (G3BP1), a stress granules (SGs) protein,^[^ ^15^ ^]^ as a regulator of p53 nuclear translocation in hepatic ferroptosis,^[^ ^16^ ^]^ but its role in diabetic cardiac injury remains uncharacterized.

Here, we investigate whether ALKBH5‐mediated m^6^A demethylation of G3BP1 modulates ferroptosis in diabetic I/R injury. We hypothesize that ALKBH5 enhances G3BP1 expression to sequester YBX1 and p53 in the cytoplasm, inhibiting their nuclear translocation and subsequent repression of the ferroptosis‐suppressive gene SLC7A11. This study aims to define the ALKBH5‐G3BP1‐p53 axis as a novel epigenetic mechanism in diabetic myocardial injury, providing insights into targeted therapeutic strategies.

## 2 Experimental Section

### 2.1 Reagents

Low‐glucose Dulbecco's modified Eagle's medium (DMEM, Cat No. 11885084) was purchased from Gibco (Grand Island, USA). Fetal bovine serum (FBS, Cat No. 11011–8611) was purchased from TIANHANG (Hangzhou, China). A Cell Counting Kit‐8 (CCK‐8, Cat No. C0037), a lactate dehydrogenase (LDH) kit (Cat No. C0016), a reactive oxygen species (ROS) kit (Cat No. S0033S), a glutathione (GSH) kit (Cat No. S0053), and a nuclear and cytoplasmic protein extraction kit (Cat No. P0028) were purchased from Beyotime (Shanghai, China). A rat p53 enzyme‐linked immunosorbent assay (ELISA) kit (Cat. No. BG‐RAT11726) was purchased from Novatein Biosciences (Boston, USA). An iron ion detection kit (Cat. No. ab83366) was purchased from Abcam (Cambridge, UK). Streptozotocin (STZ, Cat No. 18883‐66‐4) was obtained from Beijing Boaigang Biological Technology Co., Ltd., and 2,3,5‐triphenyltetrazolium chloride (TTC, Cat No. 298‐96‐4) was obtained from Wuhan Servicebio Technology Co., Ltd. Erastin (Cat No. S7242) was purchased from Selleck (Houston, TX). Rabbit anti‐rat ALKBH5 (Cat No. 16837‐1‐AP), G3BP1 (Cat No. 13057‐2‐AP), GPX4 (Cat No. 30388‐1‐AP), YBX1 (Cat No. 20339‐1‐AP), GAPDH (Cat No. 60004‐1‐Ig) α‐tubulin (Cat No. 11224‐1‐AP) primary antibodies, Cy3‐conjugated AffiniPure goat anti‐rabbit IgG (H+L) secondary antibodies (Cat No. SA00009‐2), and fluorescein (FITC)‐conjugated AffiniPure goat anti‐mouse IgG (H+L) secondary antibodies (Cat No. SA00003‐1) were purchased from Proteintech (Wuhan, China). A rabbit anti‐rat SLC7A11 primary antibody (Cat RNAiso Plus Kit, Cat. No. 9108Q), PrimeScript RT reagent (Cat. No. 9108Q), and SYBR Premix Ex Taq Kit (Cat. No. RR420A) were purchased from Takara (Dalian, China).

### 2.2 Animal Experiment Grouping and Intervention

A model of diabetic myocardial ischemia‒reperfusion injury (DIR) was established, ALKBH5 and YBX1 were overexpressed using adeno‐associated viruses. SPF healthy adult male Sprague–Dawley (SD) rats, 6 weeks old and weighing 210–240 g, were purchased from Shubeili (Wuhan) Biotechnology Co., Ltd. (license number SCXK (Xiang) 2019‐0004). The animal experimental protocol was approved by the Animal Experiment Ethics Committee of Renmin Hospital of Wuhan University, with the ethics number WDRM (Welfare) No. 20230706C. Figure 2A shows the animal experimental process, 6‐week‐old male SD rats were adaptively fed for 1 week, followed by an intraperitoneal injection of 65 mg kg^−1^ of STZ. If the fasting blood glucose level was ≥16.7 mmol L^−1^ after 1 week, the diabetic model was successfully established. After being fed again for 6 weeks, adeno‐associated virus was injected through the tail vein to overexpress ALKBH5 and YBX1. Four weeks later, the diabetic rats were subjected to cardiac ischemia‒reperfusion injury, with 30 min of cardiac ischemia followed by 120 min of reperfusion. ECG, echocardiography, LSCI, and moorO2Flo‐2 levels were detected before ischemia. Myocardial tissue and serum samples were collected after reperfusion.

For the diabetic rat model, 65 mg kg^−1^ of 1% STZ was injected intraperitoneally after the rats were fasted for 12 h. After 5 d, the rats were fasted for 6 h, and the fasting blood glucose value was greater than 16.7 mmol L^−1^. The corresponding symptoms (polydipsia, polyphagia, polyuria, and weight loss) appeared, and the diabetic rat model was successfully established. Thereafter, the rats' diet and water intake were recorded daily, and blood glucose and body weight were monitored weekly. Adeno‐associated virus (AAV) intervention was started 6 weeks after the model was established, and the ischemia‒reperfusion model was established after feeding for 4 weeks. For the DIR model, DM rats were fasted for 12 h before surgery, anesthetized via an intraperitoneal injection of 1% sodium pentobarbital (60 mg kg^−1^), fixed on the experimental table, connected to an ECG monitor, and connected to a ventilator after endotracheal intubation for mechanically controlled breathing. The right internal jugular vein was separated to establish venous access, the right femoral artery was separated and cannulated to record invasive arterial pressure, and needle electrodes were inserted subcutaneously into the limbs and chest of the rats and connected to a small animal electrocardiograph. The chest skin was disinfected with 75% ethanol solution, and a 1 cm incision was made beside the left sternum. The skin was separated and exposed layer by layer, and the third and fourth ribs were cut. The hooks were used to stretch the pericardium along the intercostal space to fully expose the heart. A ligature was inserted ≈2 mm below the left anterior descending coronary artery (LAD) branch with a 6‐0 suture for ligation. During ligation, a thin plastic tube with a diameter of 0.2 cm was passed through the ligature and clamped with microhemostatic forceps. After stabilization for 10 min, the LAD was ligated to cause myocardial ischemia. After 30 min, the ligature was loosened, and the mixture was reperfused for 2 h. The criteria for successful LAD ligation were as follows: the precordial area turned white, and ST segment elevation appeared in the electrocardiogram lead II (the T wave was tall, fused with the QRS wave, and the QRS wave was widened and taller) for successful ligation. The successful reperfusion criteria were as follows: the S‐T segment fell back, and the apex was red again.

### 2.3 Cell Experiment Grouping and Intervention

A cell high‐glucose hypoxia‐reoxygenation (HH/R) model was constructed, and ALKBH5, G3BP1, and YBX1 were overexpressed using lentivirus. Figure 3A shows the process of the cell experiment. H9c2 rat embryonic cardiomyocytes were purchased from the China Collection of Culture Collections of Wuhan University. DMEM medium containing 10% FBS was used for culture, and the cells were subcultured and grouped when they reached ≈80% confluence in the culture flask. A HH/R model was constructed for H9c2 and Neonatal Mouse Ventricular Cardiomyocytes (NMVCs) cells. First, the cells were synchronized with DMEM medium containing 1% FBS for 12 h, and then, lentivirus transfection was performed to overexpress ALKBH5, G3BP1, and YBX1. After 24 h, 10 µmol L^−1^ erastin was added to the cell culture medium, and 30 mmol L^−1^ of high glucose was administered after 48 h. The HH/R model was established 66 h later, with 4 h of hypoxia and 2 h of enriched oxygen. After 72 h, the cells and supernatants were collected for subsequent experiments.

The cells in the HG group were injected with 50% glucose to achieve a final glucose concentration of 30 mmol L^−1^. After incubation for 24 h, the cells in the HR group were placed in a three‐gas incubator at 37 °C, 95% N_2_, and 5% CO_2_ for 4 h of hypoxia. After completion, they were immediately placed in a constant‐temperature aerobic incubator at 37 °C and 5% CO_2_ for 2 h. HHR group cells were further established with an HR model based on the HG cell model. The cells in the HH/R+erastin group were stimulated with erastin 24 h before the HH/R model was established. For the intervention of the LV‐ALKBH5/G3BP1/YBX1 and siG3BP1/YBX1/p53 groups, the cells were transfected with a plasmid overexpressing ALKBH5/G3BP1/YBX1 using a lentivirus 48 h before the establishment of the HH/R model, and the cells were subsequently transfected with siRNA to knock down G3BP1/YBX1/p53. The process of the transfection of siRNA and lentivirus was performed according to previous studies.^[^ ^17^, ^18^ ^]^

The steps for isolating and culturing primary rat cardiomyocytes were as follows: First, 1–3‐day‐old suckling mice were soaked in 75% ethanol for disinfection. The chest was opened to remove the heart, which was then placed in precooled PBS. The connective tissue at the bottom of the heart was removed, and the apical tissue was removed. The samples were cut into pieces and washed with PBS at least 3 times. At least 3 times, a volume of digestion solution (containing 0.04% type II collagenase and 0.08% trypsin) was added, the mixture was placed in a 37 °C constant‐temperature water bath for digestion for 10 min, and the supernatant was discarded. The digestion solution was added again for 8 min, the supernatant was extracted, and the mixture was incubated with serum. The digestion solution was added again for 8 min, the supernatant was extracted, and the mixture was incubated with serum until the tissue was completely digested. The collected supernatant was passed through a 100‐mesh sieve. The filtrate was centrifuged at 300 × g for 5 min. The supernatant was discarded, and the cell culture bottle was spread. The mixture was placed in an incubator for differential attachment twice, each time for 50–60 min.

### 2.4 Cardiac Ultrasound, MoorO2Flo‐2 and Laser Speckle Contrast Imaging (LSCI) Detection

After anesthesia, each group of rats underwent echocardiography via a Doppler ultrasound diagnostic instrument to measure the following indicators: heart rate (HR), left ventricular ejection fraction (LVEF%), diastolic left ventricular internal diameter (LVIDd), and systolic left ventricular internal diameter (LVIDs). The precordial tissue was surgically exposed according to the instructions for the use of moorO2Flo‐2 and LSCI, and the precordial blood flow was detected via laser speckle contrast imaging technology and PeriFlux6000 technology.

### 2.5 Determination of the Myocardial Infarction Area, HE Staining, Electron Microscopy, and Immunofluorescence Detection

After reperfusion, 6 rats were randomly selected from each group, and the LAD was ligated again. One milliliter of 3% Evans blue was injected into the left ventricular cavity. The residual blood was washed with precooled saline, and the dye was distributed in the myocardium through the coronary artery. The heart was quickly removed and stored at −20 °C for 2 h. The slices were cut perpendicular to the long axis of the myocardium with a thickness of 2 mm, incubated in 1% TTC buffer at 37 °C in the dark for 15 min, and fixed in 4% paraformaldehyde for 30 min before being scanned and filmed. The Evans blue‐stained part is the viable myocardial tissue, which is called normal myocardial tissue (nonischemic myocardium). The TTC‐stained area is brick red (ischemic myocardium), and the non‐TTC‐stained area is gray (infarcted myocardium). The sizes of different regions of each slice were analyzed via Image‐Pro Plus 6.0 software. The percentages of the myocardial ischemic area and infarct area were measured and are represented by the percentage of the ischemic myocardial area to the left ventricular area (AAR/LV%) and the percentage of the infarcted myocardial area to the ischemic myocardial area (IA/AAR%).

Hematoxylin‐eosin (HE) staining was used to detect pathological changes in the myocardial tissue. After 2 h of reperfusion, the apical tissue was cut, fixed with 4% paraformaldehyde solution, embedded in paraffin, and sliced with a microtome. The pathological morphology of the myocardium was observed under a microscope after HE staining. Ultrastructural changes in the myocardial tissue and cells were detected via electron microscopy. For myocardial tissue, the apical tissue was cut after 2 h of reperfusion. The myocardial tissues of each group were fixed with 2.5% glutaraldehyde solution, embedded and sliced, and the ultrastructural changes in the heart were observed under an electron microscope.

### 2.6 Cell Activity and Biochemical Index Detection

In total, 10 000 cells per well of a 96‐well plate were used, with 6 replicates per group, and the treatment reagents were added to the 96‐well plate in turn, with a final volume of 100 µL. After 24 h of culture, 10 µL of CCK‐8 solution was added to each well for microplate reader detection. The intervention group absorbance value (A value), control value (B value, cells without intervention), and blank control well (C value, no cells with culture medium) was detected at 450 nm using a microplate reader. The cell viability was calculated according to the following formula: Cell activity (%) = (A value‐ C value)/(B value‐ C value) × 100%.

The serum and cell supernatants of the rats in each group were collected and centrifuged at 3000 r min^−1^ for 15 min at 4 °C, and the concentrations of CK‐MB and LDH were determined according to the instructions of the kits. The serum of the rats in each group was collected and centrifuged, and the levels of myocardial Fe^2+^ and MDA were detected using an automatic blood biochemical analyzer.

### 2.7 RT‒PCR Detection of mRNA Levels in Cardiac Tissues and Cells

The primer sequences are shown in Table S1 (Supporting Information), as described previously.^[^ ^1^ ^]^ Total RNA was extracted from the myocardial tissues of each group using an RNAiso Plus kit (Cat. No. 9108Q; Takara, Dalian, China). Total RNA was reverse transcribed into cDNA using the PrimeScript RT reagent (Cat. No. 9108Q; Takara, Dalian, China). The reverse transcription program was 37 °C for 15 min and 85 °C for 5 s. qRT‒PCR was performed using a SYBR Premix Ex Taq kit (Cat. No. RR420A, Takara, Dalian, China). The PCR program was set at 95 °C for 5 s, followed by 95 °C for 5 s and 60 °C for 34 s for a total of 40 cycles. The expression levels of all the genes were calculated using the 2^−ΔΔCT^ method.

### 2.8 Western Blot Detection

The sample protein was added to loading buffer and boiled for 10 min, and 20 µg of sample was quantitatively loaded in each well according to the protein concentration. The protein was transferred to a PVDF membrane by constant voltage electrophoresis at 100 V and electrotransfer at 200 mA. The membrane was blocked with skim milk powder for 1 h, incubated with a specific antibody (1:1000) at 4 °C overnight, and washed with washing buffer 3 times for 5 min each. The samples were then incubated with a rabbit anti‐goat antibody (1:1000) for 1 h and washed with washing buffer 3 times for 5 min each. The membrane was scanned and analyzed using an Odyssey fluorescence infrared imaging system, and the gray value was read.

### 2.9 Dual‐Luciferase Assay

The plasmid and mutant plasmid (WT, Mut 142, Mut 173) were inserted into the two sites 142 and 173. The WT and Mut plasmids were introduced into H9c2 and NMVC cells, respectively, and luciferase activity was detected using a dual luciferase assay kit. The well‐grown H9c2 and NMVC cells were seeded into a 24‐well plate at a density of 30% and transfected after the cells were firmly attached to the wall. When the cells were ≈40–50% dense, the reporter gene plasmids WT, Mut 142, and Mut 173 were transfected. Each sample contained 3 replicate wells. The complete culture medium was added to the Lipo3000 transfection reagent, which was subsequently diluted and mixed thoroughly. WT and Mut plasmid configuration: The WT and Mut plasmid mixtures were diluted with complete culture medium and mixed thoroughly. The sh‐NC and sh‐ALKBH5 plasmids were diluted with complete culture medium and mixed thoroughly. Each reagent mixture was incubated at room temperature for 5 min and mixed gently. The WT or Mut plasmid dilution, sh‐NC or sh‐ALKBH5 plasmid dilution and the corresponding transfection reagent dilution were mixed, and the mixture was incubated at room temperature for 20 to 30 min. After incubation, complete culture medium was added, mixed gently, and then added evenly to the corresponding wells of each group (3 replicate wells per group) for cell transfection.

After sufficient time of transfection (generally 48 h), the cells were removed, and the culture medium was removed. The samples were washed three times gently, and the PBS in each well was aspirated three times. Five layers of PLB (lysis buffer) were diluted into 1 layer of PLB liquid in advance, and the mixture was maintained at room temperature. Then, 100 µL of PLB lysis buffer was added to each well, and the samples were incubated on a shaker for 15 min. The lysis buffer was transferred to a 1.5 mL EP tube without nuclease, the tube was centrifuged (12 000 × g, 30 s), and the supernatant was separated for use. The Luciferase Assay Substrate reagent in the Luciferase Assay Buffer II liquid provided in the kit was dissolved, divided into 1.5 mL lightproof EP tubes, stored at −80 °C, and returned to room temperature before use. The Stop & Glo mixture was prepared as follows: 50 × Stop & Glo substrate was mixed with Stop & Glo buffer at a ratio of 1:50, and the mixture was maintained at room temperature. A light‐proof 96‐well ELISA plate was used, 100 µL of preprepared Luciferase Assay Reagent II and 10 µL of cell lysis supernatant (extracted in step 5) were added to each well, the mixture was mixed thoroughly, and the fluorescence intensity (firefly luciferase absorbance) was measured with an ELISA reader. Then, 100 µL of preprepared Stop&Glo mixture was added to each well, the mixture was mixed thoroughly, and the fluorescence intensity (Renilla luciferase absorbance) was measured with an ELISA reader. The absorbance value of each well was recorded: RLU1—firefly enzyme absorbance value, RLU2—Renilla enzyme absorbance value, and the ratio of the two groups, i.e., RLU1/RLU2, were calculated. Software was used for statistical analysis, and the experimental data were analyzed after three repetitions.

### 2.10 Statistical Analysis

GraphPad Prism 9.0 statistical software was used for statistical analysis. The experimental data are expressed as the means ± standard deviations. All continuous data were first assessed for normality using the Shapiro–Wilk test. For normally distributed data with homogeneous variance, Student's *t*‐test (for two groups) was used or one‐way ANOVA with Tukey's post‐hoc test (for multiple groups). For non‐normally distributed data, non‐parametric tests (Mann–Whitney U test or Kruskal‐Wallis test with Dunn's post‐hoc) were applied. Categorical data were analyzed using Fisher's exact test or chi‐squared test as appropriate. *p* < 0.05 was considered statistically difference. *p* < 0.01 was considered a significant statistically difference.

### 2.11 Ethics Approval

The animal study was reviewed and approved by the Animal Research Committee of the Renmin Hospital of Wuhan University.

## 3 Results

### 3.1 Changes in ALKBH5 and GPX4 Levels in Patients with Diabetic Ischemic Cardiomyopathy

**Figure 1:**
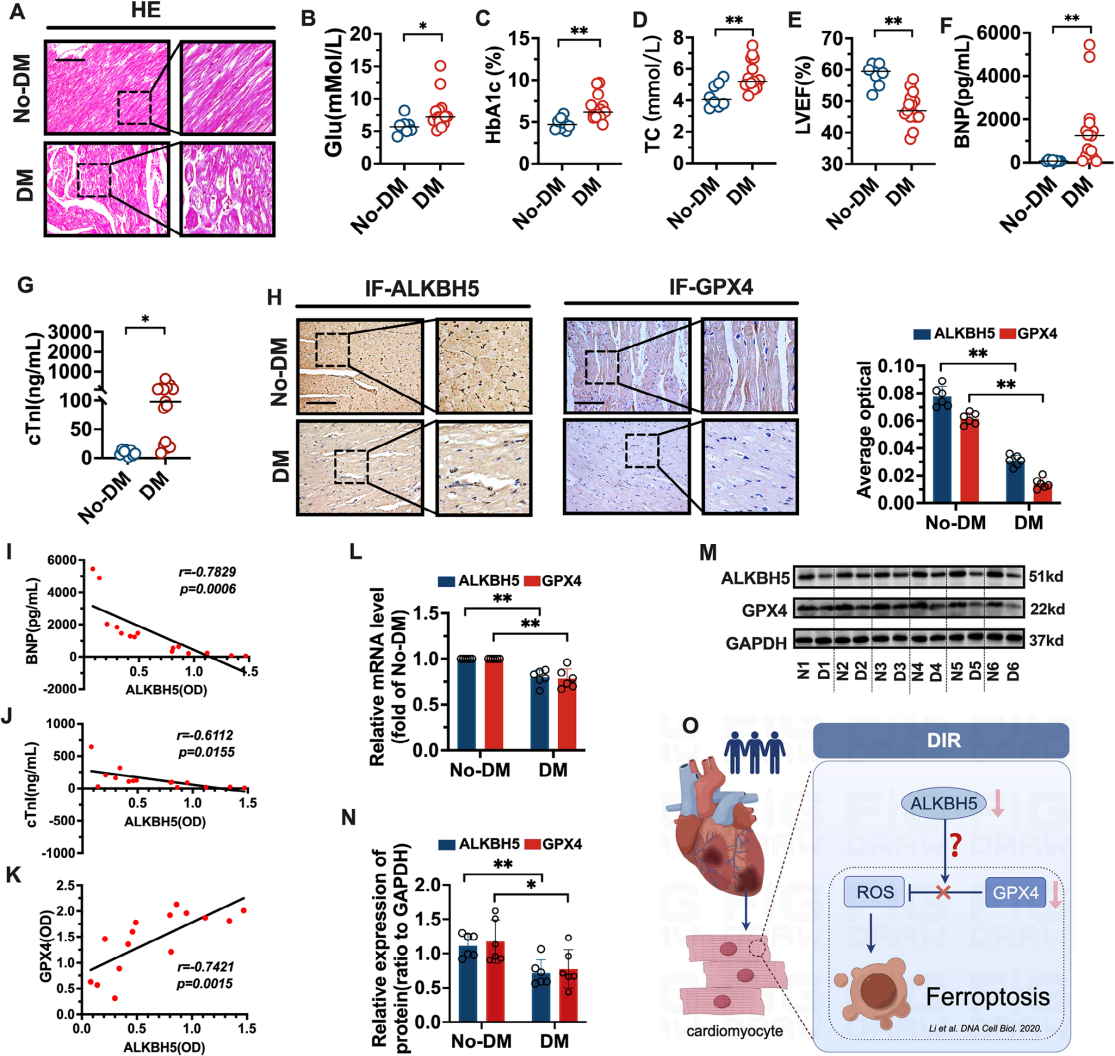
Alterations in ALKBH5 and GPX4 in diabetic (DM) and non‐diabetic (No‐DM) conditions relevant to cardiac parameters. A) Hematoxylin and Eosin (HE) staining was used to detect the pathological changes of myocardial tissue. B–G) The levels of serum glucose (Glu), glycated hemoglobin (HbA1c), total cholesterol (TC), left ventricular ejection fraction (LVEF), serum B‐type natriuretic peptide (BNP) and cardiac troponin I (cTnl) were detected by biochemical analyzer or echocardiography. H) Immunohistochemistry (IHC) was used to detect the protein expression of ALKBH5 and GPX4 in myocardial tissue. I–K) The correlation between BNP levels and ALKBH5 optical density (OD), cTnl levels and ALKBH5 OD, GPX4 OD and ALKBH5 OD. L) The mRNA levels of ALKBH5 and GPX4 in myocardial tissue were detected by RT‐PCR. M,N) The protein levels of ALKBH5 and GPX4 in myocardial tissue were detected by western blot (N1‐N6 for No‐DM, D1‐D6 for DM) O) Schematic illustration depicting the possible mechanism where reduced ALKBH5 may be associated with increased reactive oxygen species (ROS), down‐regulation of GPX4, and subsequent induction of ferroptosis in cardiomyocytes in diabetic ischemic cardiomyopathy (DIR). The schematic diagram in the dashed box illustrates our previous finding that the DIR process was accompanied by cardiomyocyte ferroptosis.^[^ ^19^ ^]^ The data was expressed as means ± SD. *n* = 8 in No‐DM group and *n* = 15 in DM group for Figure A–K. *n* = 6 in No‐DM group and DM group for Figure L–N. Continuous data were tested for normality using the Shapiro–Wilk test. Normally distributed data were analyzed by Student's *t*‐test (two groups) or one‐way ANOVA with Tukey's post‐hoc test (multiple groups). Correlation analyses I–K) used the Pearson correlation coefficient (data confirmed normally distributed). The scale bars in Figure A and H represent 100 µm. ^**^ *p* < 0.01. ^*^ *p* < 0.05. *t*‐test and Pearson correlation coefficient analysis were used.

The clinical experimental part of this study was approved by the Ethics Committee of Renmin Hospital of Wuhan University (WDRY2024‐K205). A total of 15 left ventricular cardiac specimens were obtained from patients with ischemic cardiomyopathy complicated by diabetes mellitus (DM) who were scheduled to undergo heart transplantation. As controls, 8 cardiac samples were collected from normal heart donors deemed ineligible for transplantation due to non‐cardiac reasons (No‐DM). Written informed consent was obtained from all donors and their families in advance. All procedures involving human tissue specimens were reviewed and approved by the Ethics Committee of Renmin Hospital of Wuhan University, and conducted in strict compliance with the principles outlined in the Declaration of Helsinki. The baseline characteristics of the participants were shown in Tables S2 and S3 (Supporting Information). As shown in **Figure** **1**A, the morphology of cardiomyocytes in normal human myocardial tissue was normal, the cells were arranged regularly and neatly, and the intercellular gap was small. The myocardial tissue of patients with diabetic ischemic cardiomyopathy has a disordered arrangement of myocardial cells, broken myocardial fibers, and a large intercellular space. As shown in Figure 1B–G, the levels of blood glucose, BNP, cTnl, HbA1c, and TC in patients with diabetic ischemic cardiomyopathy were significantly greater than those in normal subjects (*p* < 0.01 or 0.05), and the LVEF was significantly lower (*p* < 0.01). As shown in Figure 1H, ALKBH5 and GPX4 protein levels were significantly lower in the myocardial tissues of patients with diabetic ischemic cardiomyopathy than in normal human myocardial tissues. Then, we performed correlation analysis between ALKBH5 and BNP, cTnl, and GPX4 in patients with diabetic ischemic cardiomyopathy. As shown in Figure 1I–K, ALKBH5 was negatively correlated with BNP (*r = 0.7829, p = 0.0006*) and cTnl *(r = 0.6119, p = 0.0155*), and positively correlated with GPX4 (*r = 0.7421, p = 0.0015*). As shown in Figure 1L–N, the mRNA and protein levels of ALKBH5 and GPX4 were significantly lower in the myocardium of patients with diabetic ischemic cardiomyopathy than in the normal human myocardium (*p* < 0.01 or 0.05). There were changes in the ALKBH5 and GPX4 levels in patients with diabetic ischemic cardiomyopathy. The increase in blood lipid and blood glucose levels indicated that glucose and lipid metabolism was significantly abnormal in patients with diabetic cardiomyopathy. Moreover, the changes in myocardial histopathology and BNP, cTnl, and LVEF levels indicated that the myocardial tissue structure and cardiac pumping function were impaired. As shown in Figure 1O, the levels of the RNA methylase ALKBH5 and the ferroptosis marker GPX4 were significantly decreased in diabetic cardiomyopathy patients with myocardial tissue injury. Our previous finding that the DIR process was accompanied by cardiomyocyte ferroptosis.^[^ ^19^ ^]^ Whether ALKBH5‐ and GPX4‐mediated ferroptosis is involved in myocardial tissue injury in diabetic cardiomyopathy remains unknown.

### 3.2 Effects of the RNA Demethylase ALKBH5 on Ferroptosis Levels in the DIR Model

Figure S1 (Supporting Information) showed that the diabetic myocardial ischemia‐reperfusion injury model was successfully constructed with impaired cardiac function. Figure S2 (Supporting Information) indicates increased m^6^A levels and ferroptosis markers in HH/R cells and DIR animals, with ALKBH5 downregulation linked to myocardial and mitochondrial damage. Figure S3 (Supporting Information) verified ALKBH5 and GPX4 expression peaked at 2 h post‐reperfusion, with acute injury reaching its climax at this time point. Figure S4 (Supporting Information) was first validated the overexpression of AAV interventions for ALKBH5.

**Figure 2:**
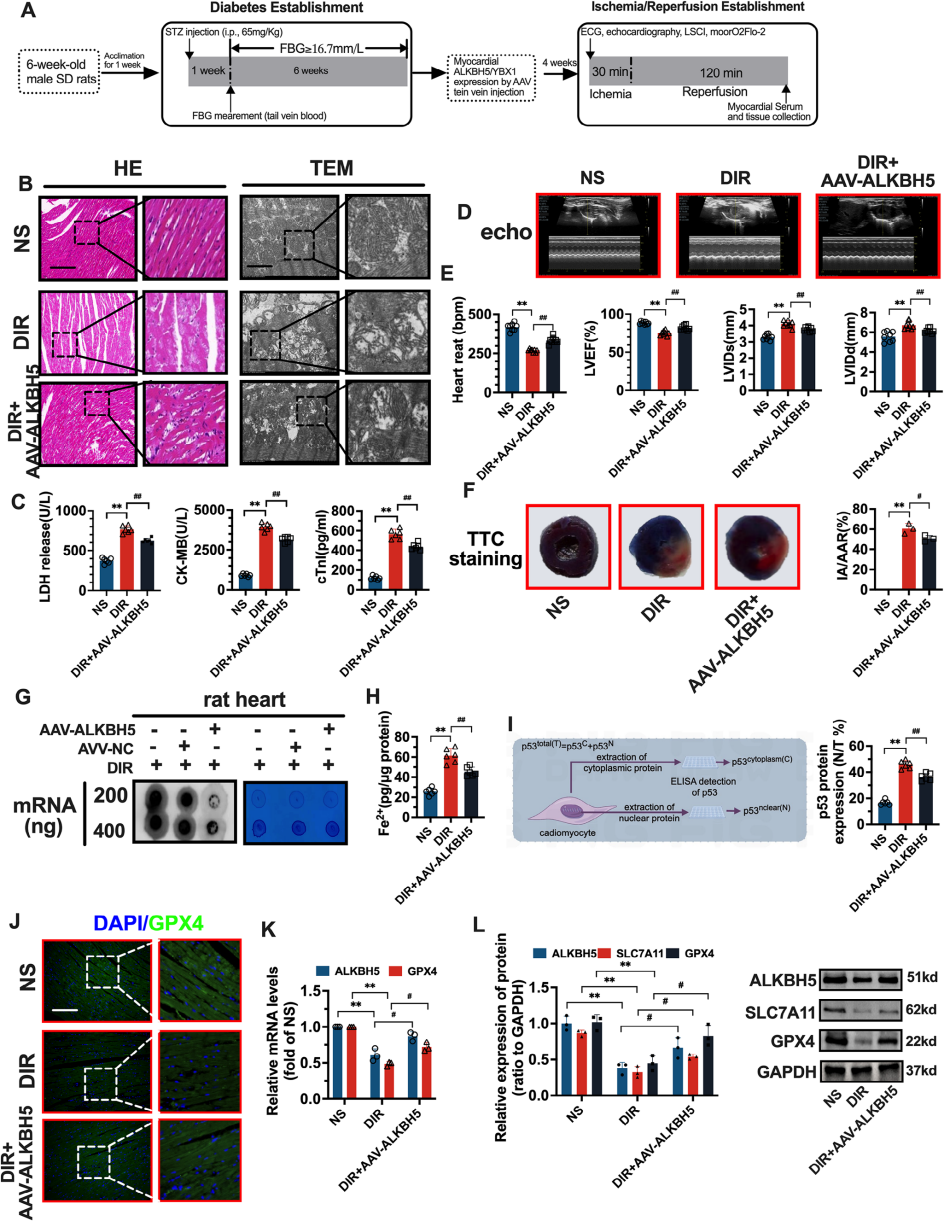
Effect of RNA demethylase ALKBH5 on ferroptosis levels in Diabetic Ischemia/Reperfusion (DIR) models. A) Schematic of DIR model establishment in 6‐week‐old male SD rats: 60 mg kg^−1^ streptozotocin‐induced diabetes + left coronary artery occlusion for 30 min/reperfusion for 24 h. B) HE staining (left) and Transmission Electron Microscopy (TEM) (right) of myocardial tissue from NS group (normal saline control), DIR group (diabetic I/R model), and AAV‐ALKBH5 group (AAV9‐ALKBH5 treated via tail vein, 1 × 10¹^2^ vg kg^−1^, 4 weeks prior to I/R). C) Serum levels of Lactate Dehydrogenase (LDH), Creatine Kinase‐MB (CK‐MB), and cTnl in different groups were detected 24 h post‐reperfusion. D) Echocardiogram (echo) images of the heart in different groups 48 h post‐reperfusion. E) Cardiac parameters, including heart rate, Left Ventricular Ejection Fraction (LVEF), Left Ventricular Internal Diameter in Diastole (LVIDd), and other measurements in different groups, with statistical annotations. F) Triphenyltetrazolium Chloride (TTC) staining (left) of heart sections and calculation of Infarct Area/Affected Area Ratio (IA/AAR) (right) in different groups 24 h post‐reperfusion. G) Dot blot was used to detect the global level of m^6^A in myocardial tissue of rats in each group. H) Measurement of iron ion (Fe^2^⁺) levels in protein samples of different groups. I) Schematic illustration of p53 protein detection mechanism, including cytoplasmic and nuclear protein extraction and ELISA detection. J) Immunofluorescence (IF) images showing co‐localization of DAPI and GPX4 in rat myocardial tissue of different groups. K,L) The expression of ALKBH5 and GPX4 mRNA/protein was detected by RT‐PCR and western blot in myocardial tissue 48 h post‐reperfusion. The data was expressed as means ± SD. *n* = 6. Normality verified by Shapiro–Wilk test. Two‐group comparisons analyzed by Student's *t*‐test. The scale bars in HE, TEM, and Figure J represent 100, 20 and 100 µm. Compared with the NS group, ^**^ *p* < 0.01, ^*^ *p* < 0.05. Compared to the DIR group, ^##^ *p* < 0.01, ^#^ *p* < 0.05.

The pathological examination of the myocardial tissue in the three groups revealed that the degree of disordered myocardial cell arrangement, myocardial fiber breakage, and widening of the intercellular space in the myocardial tissue in the AAV‐ALKBH5 group were greater than those in the DIR group, as shown in **Figure** **2**B. Electron microscopy revealed that the damage to the mitochondrial structure and interruption of mitochondrial cristae continuity in the myocardial tissue of the AAV‐ALKBH5 group were alleviated compared with those of the DIR group.

As shown in Figure 2C, compared with those in the NS group, the levels of LDH, CK‐MB, and cTnl in the serum and myocardial tissue of the DIR group were significantly greater (*p* < 0.01). Compared with those in the DIR group, the levels of LDH, CK‐MB, and cTnl in the serum and myocardial tissue of the AAV‐ALKBH5 group were significantly lower (*p* < 0.01 or 0.05). The echocardiographic results in Figure 2D show that the systolic function of the heart was significantly impaired in DIR rats compared with that in NS rats. After AAV‐ALKBH5 treatment, the cardiac systolic function of the rats was restored. As shown in Figure 2E,F, compared with those in the NS group, the levels of IA/AAR, LVIDs, and LVIDd in the serum and myocardial tissue of the DIR group were significantly greater (*p* < 0.01), and the heart rate and LVEF were significantly lower (*p* < 0.01). Compared with those in the DIR group, the levels of the levels of IA/AAR, LVIDs, and LVIDd in the serum and myocardial tissue of the AAV‐ALKBH5 group were significantly lower (*p* < 0.01 or 0.05), and the heart rate and LVEF were significantly greater (*p* < 0.01).

As shown in Figure 2G, dot blot experiments revealed that, compared with that in the normal group, the overall level of m^6^A in the DIR model group was significantly greater. Compared with that in the DIR model group, the level of m^6^A in the AVV‐ALKBH5 group was lower.

As shown in Figure 2H,I, Figure S5A–D (Supporting Information), compared with those in the NS group, the levels of Fe^2+^, p53^N/T^, ROS, lipid peroxidation markers (MDA, 4‐HNE), and ACSL4 mRNA level in the serum and myocardial tissue of the DIR group were significantly greater (*p* < 0.01). Compared with those in the DIR group, the levels of Fe^2+^, p53^N/T^, ROS, MDA, 4‐HNE, and ACSL4 mRNA level in the serum and myocardial tissue of the AAV‐ALKBH5 group were significantly lower (*p* < 0.01). As shown in Figure 2J and Figure S13A (Supporting Information), the expression of GPX4 in the myocardial tissue of the DIR group was greater than that in the NS group, as determined by immunofluorescence. Compared with that in the DIR group, the expression of GPX4 in the myocardial tissue of the AVV‐ALKBH5 group was significantly greater. As shown in Figure 2K,L, compared with those in the NS group, the mRNA and protein levels of ALKBH5 and GPX4 in the DIR group were significantly lower (*p* < 0.01). Compared with those in the DIR group, the mRNA and protein levels of ALKBH5 and GPX4 were greater in the AVV‐ALKBH5 group (*p* < 0.05).

### 3.3 Effects of the RNA Demethylase ALKBH5 on Ferroptosis Levels in HH/R Models

**Figure 3:**
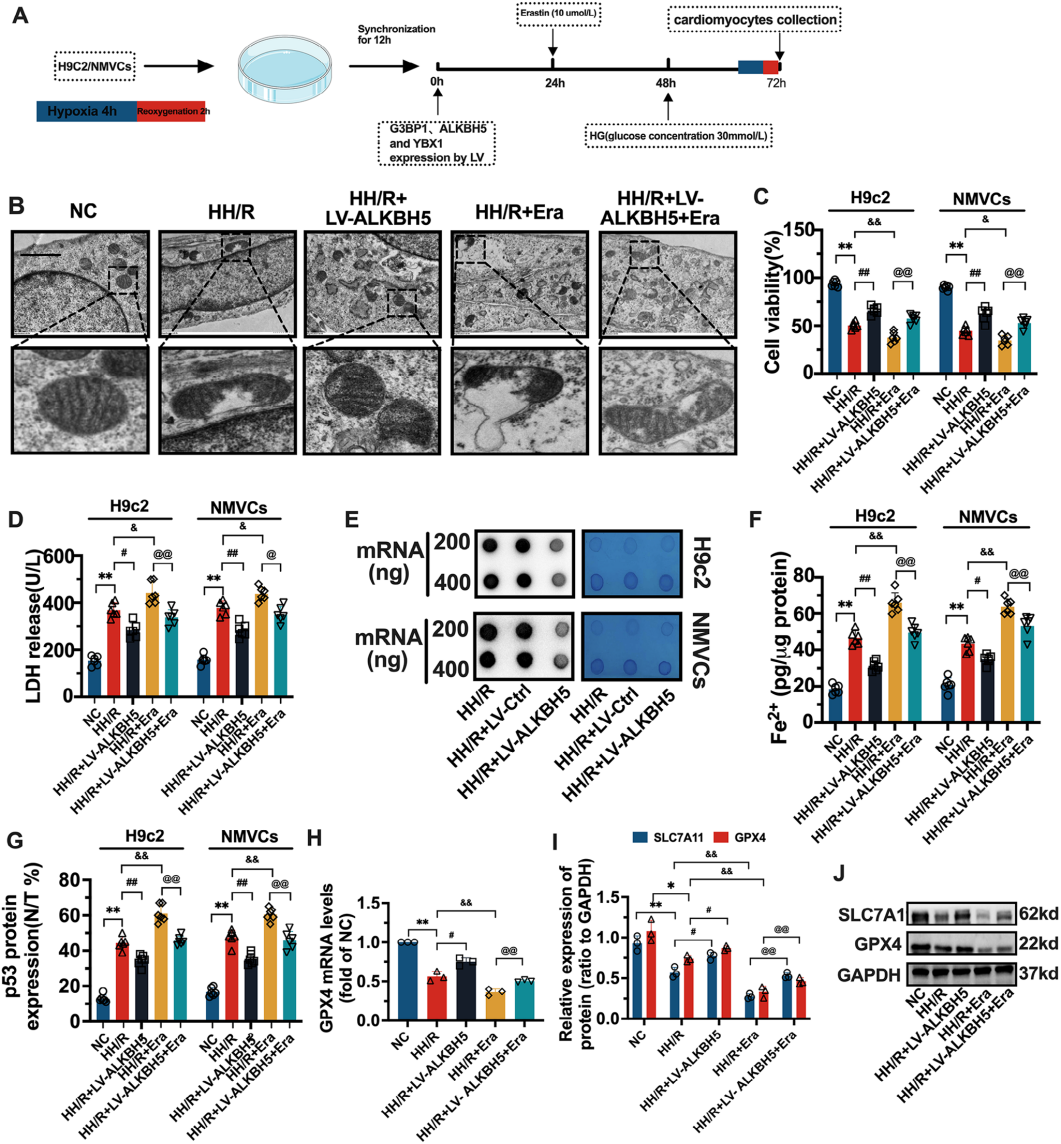
Effect of RNA demethylase ALKBH5 on ferroptosis levels in High‐glucose Hypoxia‐Reoxygenation (HH/R) model. A) Schematic diagram illustrating the experimental protocol for treating H9c2 and Neonatal Mouse Ventricular Cardiomyocytes (NMVCs) cells. The process includes hypoxia for 4 h, reoxygenation, synchronization for 12 h, and treatment with lentivirus (LV) carrying siRNAs against G3BP1, ALKBH5, and YBX1, followed by collection of cardiomyocytes at various time – points (0, 24, 48, 72 h). B) The ultrastructural changes of cells were detected by TEM. C,D) Bar graphs depicting cell viability percentages and LDH release levels in H9c2 and NMVCs cells across different experimental groups. E) Dot blot was used to detect the overall level of m^6^A in NMVCs cell model at 200 and 400 ng mRNA levels. F,G) Bar graphs presenting the levels of Fe^2^⁺ and p53^N/T^ protein levels in H9c2 and NMVCs cells for different experimental groups. H) The changes of GPX4 mRNA in each group of cells were detected by RT‐PCR. I,J) western blot was used to detect the changes of SLC7A11 and GPX4 protein in each group of cells. The data was expressed as means ± SD. *n* = 6 for Figure B–D, F,G. *n* = 3 for Figure H–J. Data were tested for normality via the Shapiro–Wilk test and confirmed to be normally distributed. For multiple group comparisons, one‐way ANOVA with Tukey's post‐hoc test was used; for two‐group comparisons, Student's *t*‐test was applied. The scale bars in Figure B represent 20 µm. ^**^ *p* < 0.01, ^*^ *p* < 0.05. ##*p* < 0.01, #*p* < 0.05. @@*p* < 0.01, @*p* < 0.05. &&*p* < 0.01, &*p* < 0.05.

As shown Figure S6 (Supporting Information), it was first validated the overexpression of Lentivirus (LV) interventions for ALKBH5 in H9c2 and NMVCs cells. As shown in **Figure** **3**E, dot blot experiments revealed that the overall level of m^6^A in the HH/R+LV‐Ctrl group did not change significantly compared with that in the HH/R group. Compared with that in the HH/R+LV‐Ctrl group, the overall m^6^A level in the HH/R+LV‐ALKBH5 group was significantly lower. In subsequent experiments, we overexpressed ALKBH5 in HH/R cells and treated them with the ferroptosis agonist erastin. As shown in Figure 3B, the ultrastructure of the H9c2 cells in the NC group was intact, and the continuity of the mitochondrial cristae was not damaged. The mitochondrial structure was destroyed, and the continuity of the mitochondrial cristae was disrupted in the HH/R group of cells. After LV‐ALKBH5 treatment, the damage to the mitochondrial structure and disruption of mitochondrial cristae were alleviated. However, after erastin intervention, the degree of mitochondrial damage in the cells was further aggravated compared with that in the HH/R group. Compared with that in the HH/R+Era group, the degree of mitochondrial damage was alleviated in the HH/R+LV‐ALKBH5+Era group.

As shown in Figure 3C,D,F–J, Figure S7A–D (Supporting Information), compared with those in the NC group, the levels of LDH, Fe^2+^, p53^N/T^, ROS, MDA, and 4‐HNE, and ACSL4 mRNA level in the HH/R group of H9c2 cells were significantly greater (*p* < 0.01). Cell viability and SLC7A11, GPX4 mRNA, and protein levels were significantly lower (*p* < 0.01). Compared with those in the HH/R group, the LDH, Fe^2+^, p53^N/T^, ROS, MDA, 4‐HNE and ACSL4 mRNA levels in the LV‐ALKBH5 group were significantly lower (*p* < 0.01 or 0.05), and the cell viability and SLC7A11, GPX4 mRNA and protein levels were significantly greater (*p* < 0.01 or 0.05). Compared with those in the HH/R group, the levels of LDH, Fe^2+^, p53^N/T^, ROS, MDA, and 4‐HNE, and ACSL4 mRNA level in the erastin group were further increased (*p* < 0.01 or 0.05); moreover, cell viability and SLC7A11, GPX4 mRNA and protein levels were further decreased (*p* < 0.01 or 0.05). Compared with those in the HH/R+Era and HH/R+LV‐ALKBH5 groups, the levels of LDH, Fe^2+^, p53^N/T^, ROS, MDA, and 4‐HNE, and ACSL4 mRNA level in the HH/R+Era+LV‐ALKBH5 group were decreased and increased, respectively (*p* < 0.01 or 0.05); cell viability and SLC7A11, GPX4 mRNA and protein levels were increased and decreased, respectively (*p* < 0.01 or 0.05). Similarly, the changes of the above markers in NMVC cells were consistent with those in H9c2 cells.

### 3.4 ALKBH5 can Regulate G3BP1 Through RNA Methylation

**Figure 4:**
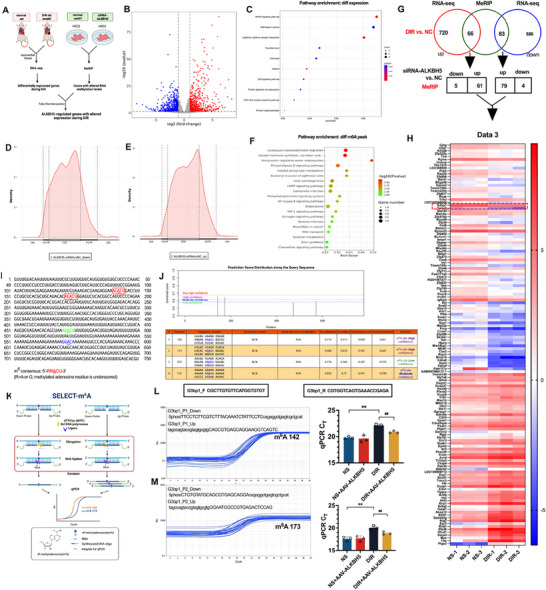
ALKBH5 can regulate G3BP1 through RNA methylation. A) Target molecules regulated by ALKBH5 were detected by RNA‐seq and MeRIP sequencing. B) volcano map of RNA‐seq sequencing. C) KEEG analysis of differential pathways after RNA‐seq sequencing. D) peak downregulation by ALKBH5‐siRNA versus NC, MeRIP sequencing. E) ALKBH5‐siRNA versus NC, peak upregulation by MeRIP sequencing. F) KEEG analysis of differential pathways after MeRIP sequencing. G) RNA‐seq and MeRIP sequencing were used to analyze differentially expressed genes. H) Cluster map of differentially expressed genes selected by RNA‐seq and MeRIP combined sequencing. I) Nucleic acid sequence of G3BP1 molecule. J) m^6^A modification sites of G3BP1 predicted by SRAMP website. K) Flowchart of SELECT‐ m^6^A kit to detect the level of m^6^A methylation sites. L,M) SELECT‐ m^6^A kit was used to detect the levels of 142 and 173 m^6^A sites in each group. The data was expressed as means ± SD. *n* = 3. Data were tested for normality using the Shapiro–Wilk test and confirmed normally distributed. Two‐group comparisons performed via Student's *t*‐test. ^**^ *p* < 0.01, ##*p* < 0.01. *t*‐test was used.

In conclusion, ALKBH5 overexpression can alleviate myocardial I/R injury in diabetic rats by attenuating ferroptosis. RNA‐seq and MeRIP sequencing were performed to identify the molecular targets of ferroptosis regulated by ALKBH5. As shown in **Figure** **4**A, three normal rats and three DIR rats were selected for RNA sequencing to identify differentially expressed genes. After ALKBH5 was knocked down by siRNA in H9c2 cells, MeRIP sequencing was performed to determine which molecules were affected by ALKBH5 in the H9c2 cells. Finally, the two sets of genes intersected, and the genes that were both altered during myocardial I/R injury and regulated by ALKBH5 RNA methylation were selected. As shown in Figure 4B, there were 786 genes with increased expression and 769 genes with decreased expression in the DIR group compared with the normal group. The number of significantly upregulated and downregulated genes was comparable, suggesting that good regulatory molecules are at a comparable level to bad regulatory molecules during pathogenesis. As shown in Figure 4C, KEGG analysis of these genes revealed that most the differentially expressed genes are involved in the MAPK signaling pathway, pathways in cancer, cytokine–cytokine receptor interactions, toxoplasmosis, the cell cycle, malaria, the p53 signaling pathway, and protein digestion and absorption. Since m^6^A is generally enriched in the vicinity of gene transcription termination sites (including the 3′‐UTR and stop codon), the modification of 3′polya in particular contributes to nuclear export, translation initiation and maintenance of the structural stability of mRNAs together with polyA‐binding proteins. As shown in Figure 4D,E, 1594 peaks were upregulated, and 263 peaks were downregulated. The number of upregulated peaks was six times greater than that of downregulated peaks. ALKBH5 itself is an RNA‐modifying protein, and demethylation modifies mRNA to promote mRNA expression by increasing RNA stability. After ALKBH5 knockdown, the demethylation effect of ALKBH5 on m^6^A modification sites was weakened, and the m^6^A level of target genes was increased. By analyzing the distribution of peak frequency in transcript regions, we found that both downregulated and upregulated m^6^A peak levels in the ALKBH5‐siRNA group were mostly distributed in the 3′‐UTR regions compared with those in the NC group.

As shown in Figure 4F, KEGG analysis of these differentially expressed m^6^A peaks revealed that most of the differentially expressed m^6^A peaks were involved in leukocyte transendothelial migration, growth hormone synthesis, vasopressin‐regulated water reabsorption, phospholipase D signaling pathway, inositol phosphate metabolism, bacterial invasion of epithelial cells, viral carcinogenesis, CAMP signaling pathway and other pathways. As shown in Figure 4G, the intersection of genes that were different between RNA‐seq and MeRIP sequencing revealed 66 m^6^A and 83 m^6^A peaks in the MeRIP dataset that were upregulated and downregulated in the RNA‐seq dataset, respectively. Further analysis of these 66 and 83 genes revealed that 5 m^6^A peaks were upregulated in RNA‐seq and downregulated in MeRIP, whereas 61 m^6^A peaks were upregulated in RNA‐seq and upregulated in MeRIP. There were 79 genes whose expression was downregulated according to RNA‐seq and upregulated according to MeRIP and 4 genes whose expression was downregulated according to RNA‐seq and downregulated according to MeRIP. From the above intersection results, most of the genes with changes in the RNA‐seq dataset corresponded to upregulated m^6^A peaks in MeRIP sequencing, and the number of upregulated m^6^A peaks was 140. Knockdown of ALKBH5 reduced the demethylation effect of ALKBH5 on m^6^A modification sites, increased the m^6^A level of target genes, and decreased the mRNA level of target genes. Among these 140 genes, the number of downregulated genes was 1.3 times greater than the number of upregulated genes in the RNA‐seq dataset, and downregulated genes accounted for the majority. This finding indicated that the number of downregulated genes was significantly greater than the number of upregulated genes after ALKBH5 knockdown. The downregulated genes in the RNA‐seq dataset generally had a positive regulatory effect on the disease, which indirectly suggested that ALKBH5 upregulation had a protective effect on myocardial I/R injury in diabetes.

As shown in Figure 4H, heatmap analysis of these 140 genes revealed that G3BP1 molecules were downregulated in the RNA‐seq dataset and upregulated in the MeRIP sequencing m^6^A peak dataset. Our previous study revealed that during acute liver failure, the NTF2 and RRM domains of the G3BP1 protein can bind to the NLS sequence of p53, preventing p53 from being incorporated into the nucleus to bind to the promoter region of SLC7A11, a ferroptosis regulator, and promote the transcription of SLC7A11, thereby inhibiting ferroptosis in hepatocytes.^[^ ^16^ ^]^ Thus, G3BP1 is regulated by both ALKBH5 and ferroptosis. In future studies, we will focus on the specific regulatory mechanisms of G3BP1 upstream and downstream. As shown in Figure 4I,J, to identify the specific m^6^A sites of G3BP1 regulated by ALKBH5, we identified 4 m^6^A modification sites on the G3BP1 molecule via SRAMP website prediction, and 2 sites were found with high confidence (142 and 173). As shown in Figure 4K–M, the SELECT‐m^6^A kit was used to detect the m^6^A levels at sites 142 and 173 in the model group and after overexpression of ALKBH5. Compared with that in the normal group, the expression of m^6^A did not change significantly after ALKBH5 overexpression, whereas the expression of m^6^A increased significantly in the HH/R group (*p* < 0.01). Compared with those in the HH/R group, the m^6^A levels in the HH/R+LV‐ALKBH5 group were significantly lower (*p* < 0.01 or 0.05).

### 3.5 G3BP1 3′‐UTR Region and the m^6^A Site After Cardiomyocyte Ferroptosis

**Figure 5:**
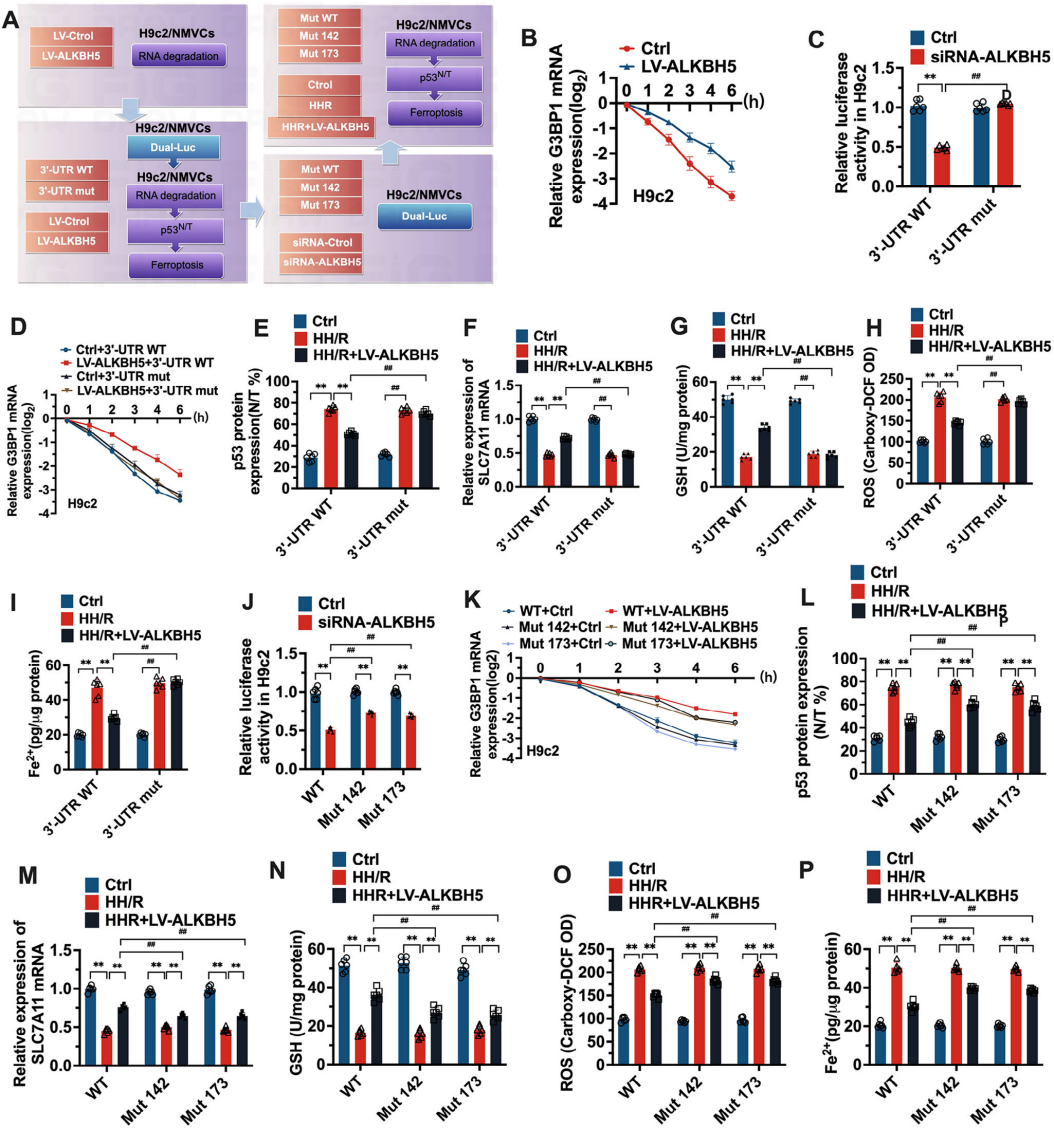
Role of G3BP1 3′UTR region and m^6^A modification site in cardiomyocyte ferroptosis. A) Schematic diagrams illustrating experimental setups in H9c2 and NMVCs cells, including treatments with LV‐Ctrl, LV‐ALKBH5, 3′‐UTR mut, and 3′‐UTR WT, Mut WT, Mut 142, Mut 173, siRNA‐Ctrl and siRNA‐ALKBH5 and manipulations to study RNA degradation and ferroptosis. LV‐Ctrl Group: Transfection with lentiviral control vector (MOI = 50, 48 h). LV‐ALKBH5 Group: Lentiviral overexpression of ALKBH5 (MOI = 50, 48 h). 3′UTR WT Group: Wild‐type G3BP1 3′UTR luciferase reporter plasmid (2 µg, 24 h). 3′UTR Mut Groups: Site‐specific mutations at m⁶A sites 142 (Mut 142) and 173 (Mut 173) (2 µg, 24 h). siRNA‐Ctrl/siRNA‐ALKBH5 Groups: Transfection with control or ALKBH5‐specific siRNA (50 nm, 72 h). B,D,K) Determination of the rate of G3BP1 mRNA degradation in H9c2 cells. Actinomycin D treatment (5 µg mL^−1^) to block transcription, G3BP1 mRNA levels measured by RT‐PCR at 0, 2, 4, 6 h post‐treatment. C,J) Dual‐luciferase assay was used to detect the fluorescence intensity of the reporter gene in H9c2 cells. Firefly/Renilla luciferase activity measured 48 h post‐transfection. Normalized to Renilla luciferase for transfection efficiency. E–I,L–P) The kits were used to detect the levels of p53^N/T^, SLC7A11 mRNA, GSH, ROS and Fe^2+^ in each group. The data was expressed as means ± SD. *n* = 6. Normality was assessed using the Shapiro–Wilk test. For comparisons between two groups, Student's *t*‐test was used. For multiple groups, one‐way ANOVA with Tukey's post‐hoc test was applied. ^**^ *p* < 0.01. ##*p* < 0.01.

**Figure** **5**A shows the effects of the G3BP1 3′‐UTR region and the m^6^A site on G3BP1 mRNA stability and ferroptosis in subsequent experiments. We first examined the effect of altered ALKBH5 expression on the stability of G3BP1 mRNA. The degradation rate of G3BP1 mRNA in H9c2 and NMVC cells at different time points was detected via RT‒PCR (line slope). As shown in Figure 5B and Figure S9A (Supporting Information), after overexpression of ALKBH5, the degradation of G3BP1 mRNA was slower in H9c2 and NMVC cells than in control cells.

The m^6^A site of G3BP1 regulated by ALKBH5 is located in 3′‐UTR. The MeRIP sequencing results suggested that the m^6^A modification of G3BP1 was highly enriched in the 3′‐UTR regions, which was predicted by the SRAMP network. The 3′‐UTR region of G3BP1 was mutated to produce a dual‐luciferase reporter plasmid (3′‐UTR Mut), and a wild‐type reporter plasmid (3′‐UTR WT) was synthesized. H9c2 and NMVC cells were transfected with siRNA‐ALKBH5 and control siRNA (Ctrl), and fluorescence was detected. The stability of 3′‐UTR was analyzed.

As shown in Figure 5C and Figure S9B (Supporting Information), ALKBH5 knockdown significantly reduced the fluorescence intensity in H9c2 and NMVC cells (*p* < 0.01). In the mutant 3′‐UTR reporter plasmid group, ALKBH5 knockdown did not significantly change the fluorescence intensity. Moreover, when ALKBH5 was knocked down, the fluorescence intensity in the mutant 3′‐UTR reporter plasmid group was significantly greater than that in the wild‐type 3′‐UTR reporter plasmid group.

As shown in Figure 5D and Figure S9C (Supporting Information), a plasmid (3′‐UTR Mut) containing the 3′‐UTR region of the mutant G3BP1 gene was constructed, and a wild‐type plasmid (3′‐UTR WT) was synthesized. H9c2 and NMVC cells were transfected with an ALKBH5‐overexpressing lentivirus (LV‐ALKBH5) or a control lentivirus (Ctrl), and the degradation rate of G3BP1 mRNA was detected and analyzed. In both H9c2 and NMVC cells, mRNA degradation was significantly reduced after ALKBH5 overexpression in the wild‐type 3′‐UTR plasmid group. There was no significant difference in the mRNA degradation rate among the 3′‐UTR mutant plasmids. In addition, there was no significant difference in the mRNA degradation rate between the mutant 3′‐UTR plasmid group and the wild‐type 3′‐UTR plasmid group. However, ALKBH5 over‐expression affected the stability of the G3BP1 3′‐UTR, which was dependent on the demethylase activity of ALKBH5 and the location of the G3BP1 mRNA 3′‐UTR.

We next examined the effect of ALKBH5 on ferroptosis after mutation of the G3BP1 3′‐UTR in an HH/R cell model. As shown in Figure 5E–I, the levels of p53^N/T^, ROS, and Fe^2+^ in the HH/R group were significantly greater than those in the Ctl group (*p* < 0.01), and SLC7A11 mRNA and GSH levels were significantly lower (*p* < 0.01). Compared with those in the HH/R group, the levels of p53^N/T^, ROS and Fe^2+^ in the HH/R+LV‐ALKBH5 group were significantly lower (*p* < 0.01), and SLC7A11 mRNA and GSH levels were significantly greater (*p* < 0.01). In the mutant 3′‐UTR plasmid group, the changes in p53^N/T^, ROS, Fe^2+^, SLC7A11 mRNA and GSH in the model group were consistent with those in the wild‐type 3′‐UTR plasmid group. However, compared with those in the model group, the levels of p53^N/T^, ROS, Fe^2+^, SLC7A11 mRNA and GSH in the HH/R+LV‐ALKBH5 group did not significantly differ. Moreover, compared with those in the 3′‐UTR WT group, the levels of p53^N/T^, ROS and Fe^2+^ in the 3′‐UTR Mut group were significantly greater (*p* < 0.01), and SLC7A11 mRNA and GSH levels were significantly lower (*p* < 0.01). These results suggest that ALKBH5 affects ferroptosis in the HH/R model through the 3′‐UTR regions of G3BP1 mRNA.

The m^6^A modification site in the 3′‐UTR was then selected according to the APC peak fragment sequence from the MeRIP‐seq results and the SRAMP website, and the “A→T” mutation was introduced to construct the 3′‐UTR dual‐fluorescence reporter plasmid with a mutation in the methylation site. The two sites of G3BP1 were mutated to produce reporter plasmids (Mut 142 and Mut 173), and the wild‐type reporter plasmid (WT) was synthesized.

As shown in Figure 5J and Figure S9D (Supporting Information), H9c2 and NMVC cells were treated with siRNA‐ALKBH5 or control siRNA (Ctrl) and transfected with reporter plasmids (WT, Mut 142 or Mut 173). The luciferase assay was subsequently performed according to the instructions of the dual luciferase kit, and the m^6^A site of the 3′‐UTR of G3BP1 was determined by the change in the fluorescence value. As shown in Figure 5K and Figure S9E (Supporting Information), ALKBH5 knockdown significantly reduced the fluorescence intensity in H9c2 and NMVC cells (*p* < 0.01). In the Mut 142 and Mut 173 reporter plasmid groups, the fluorescence intensity of the Mut 142 and Mut 173 reporter plasmid groups was significantly greater than that of the wild‐type 3′‐UTR reporter plasmid group after ALKBH5 knockdown.

We next verified the effect of ALKBH5 on ferroptosis after mutation of m^6^A at positions 142 and 173 in the HH/R cell model. As shown in Figure 5L–P, the levels of p53^N/T^, ROS, and Fe^2+^ in the HH/R group were significantly greater than those in the Ctl group (*p* < 0.01), and SLC7A11 mRNA and GSH levels were significantly lower (*p* < 0.01). Compared with those in the HH/R group, the levels of p53^N/T^, ROS, and Fe^2+^ in the HH/R group were significantly lower (*p* < 0.01), and SLC7A11 mRNA and GSH levels were significantly greater (*p* < 0.01). In the Mut 142 and Mut173 plasmid groups, the changes in p53^N/T^, ROS, Fe^2+^, SLC7A11 mRNA and GSH in the model group were consistent with those in the wild‐type 3′‐UTR plasmid group. However, compared with those in the model group, the levels of p53^N/T^, ROS, and Fe^2+^ were lower in the HH/R+LV‐ALKBH5 group (*p* < 0.01), and SLC7A11 mRNA and GSH levels were greater (*p* < 0.01). Moreover, the levels of p53^N/T^, ROS, Fe^2+^, SLC7A11 mRNA and GSH in the HH/R+LV‐ALKBH5 group were slightly greater than those in the 3′‐UTR WT group (*p* < 0.01). These results suggest that ALKBH5 affects ferroptosis in the HH/R model through methylation at sites 142 and 173 in the 3′‐UTR of G3BP1 mRNA and that mutations at either site can only partially affect ferroptosis.

### 3.6 ALKBH5 can Affect Ferroptosis Through the G3BP1–YBX1–p53 Axis

**Figure 6:**
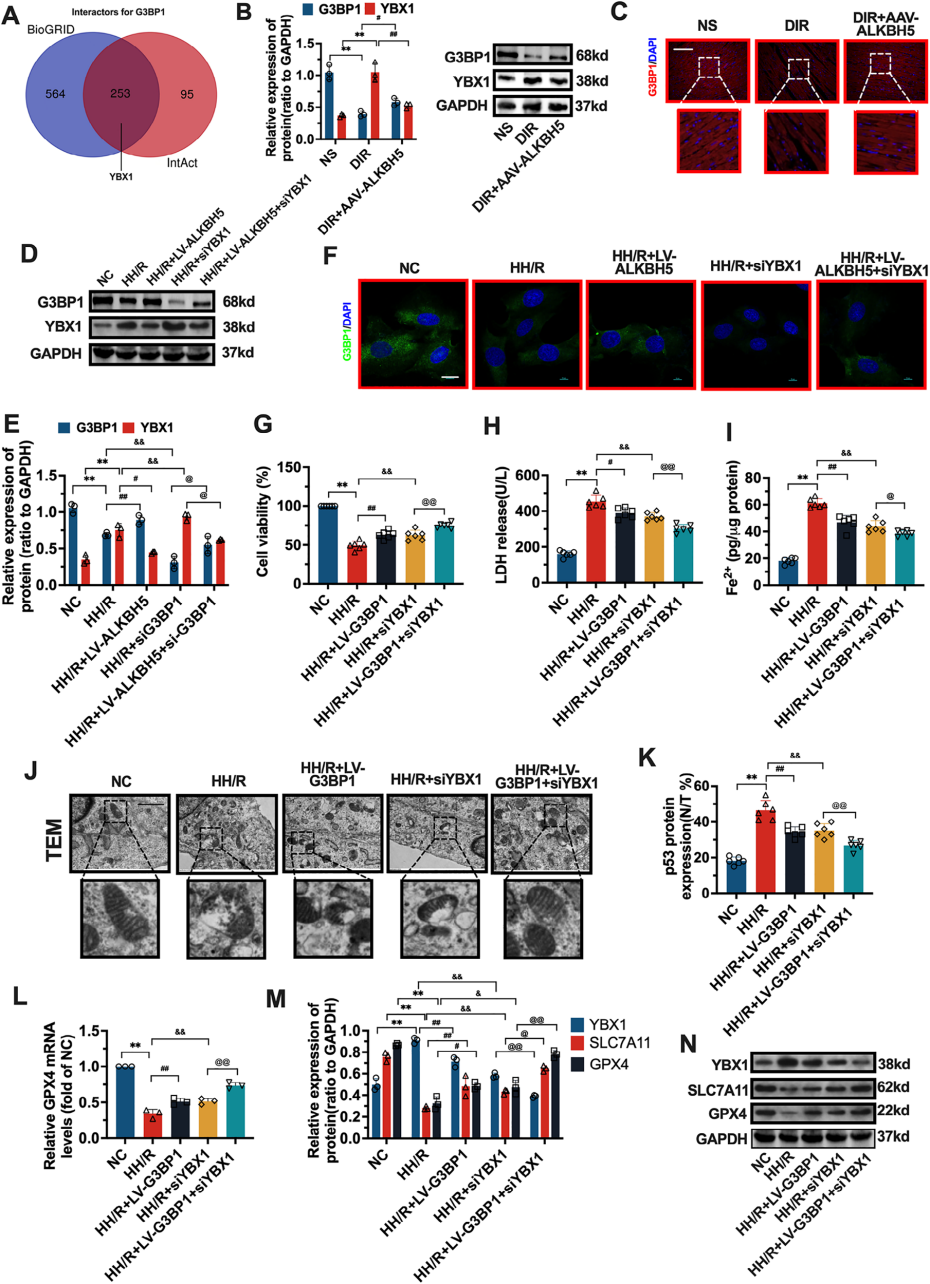
Effect of ALKBH8 on G3BP1‐YBX1‐p53 axis. A) BioGRID and IntAct database were used to search the proteins interacting with the human G3BP1 protein and make a Venn diagram. B,D,E) western blot was used to detect the changes of G3BP1 and YBX1 protein in cells and myocardial tissue. C) IF was used to detect the expression of G3BP1 protein in rat myocardial tissue. E) IF was used to detect the expression of knockdown G3BP1 protein in each group of cells. G–I) The kits were used to detect cell viability, LDH, and Fe^2+^ levels. J) Ultrastructural changes of the wash bar were detected by TEM. K) The kit was used to detect p53^N/T^ level. L) Western blot and RT‐PCR were used to detect the changes of GPX4 mRNA in H9c2 cells. (N) Western blot was used to detect the protein expression of YBX1, SLC7A11, and GPX4 in each group. The data was expressed as means ± SD. *n* = 3. Normality was confirmed using the Shapiro–Wilk test. For comparisons between two groups, Student's *t*‐test was used. For multiple groups, one‐way ANOVA with Tukey's post‐hoc test was applied. The scale bars in Figure C,F, and J represent 100, 20, and 20 µm. ^**^ *p* < 0.01, ^*^ *p* < 0.05. ##*p* < 0.01, #*p* < 0.05. @@*p* < 0.01, @*p* < 0.05. &&*p* < 0.01, &*p* < 0.05.

To further clarify how G3BP1 affects ferroptosis, since the database of G3BP1‐interacting proteins of rat origin is not complete, as shown in **Figure** **6**A, we retrieved the BioGRID (https://thebiogrid.org/115448) and IntAct (https://www.ebi.ac.uk/intact/search?query = id:Q13283^*^#interactors) protein databases and revealed that 817 and 348 proteins interact with the human G3BP1 protein, respectively. A total of 253 common interacting proteins were found. Among 253 common interactors, YBX1 was prioritized due to its established role in p53 signaling and stress granule assembly, the latter of which is dysregulated in ferroptosis.^[^ ^16^, ^20^ ^]^ Subsequent co‐immunoprecipitation validated direct G3BP1‐YBX1‐p53 interactions. However, it was not clear which amino acid domain of the G3BP1 protein could bind to YBX1. Therefore, we selected YBX1 as our research object.

As shown in Figure S10 (Supporting Information), it was first validated the over‐expression of LV interventions for G3BP1, and down‐expression of siRNA interventions for G3BP1 and YBX1 in H9c2 cells. We then verified the effect of ALKBH5 on the G3BP1–YBX1 axis, as shown in Figure 6B,C and Figure S13B (Supporting Information). Compared with the normal group, the protein level of G3BP1 in the DIR group was significantly lower (*p* < 0.01), and the protein level of YBX1 was significantly greater (*p* < 0.01). Compared with that in the DIR group, the protein level of G3BP1 in the AAV‐ALKBH5 group was significantly greater (*p* < 0.05), and the protein level of YBX1 was significantly lower (*p* < 0.01).

ALKBH5 overexpression combined with G3BP1 knockdown was subsequently used to detect changes in the G3BP1–YBX1 axis. As shown in Figure 6D,E, compared with that in the NC group, the protein level of G3BP1 was significantly lower in the HH/R group (*p* < 0.05), and the protein level of YBX1 was significantly greater (*p* < 0.01). Compared with those in the HH/R group, the G3BP1 protein level in the LV‐ALKBH5 group was significantly greater (*p* < 0.01), the YBX1 protein level was significantly lower (*p* < 0.01), the G3BP1 protein level was lower in the siRNA‐G3BP1 group (*p* < 0.01), and the protein level of YBX1 was further increased. Compared with the siRNA‐G3BP1 group, the LV‐ALKBH5 + siRNA‐G3BP1 group presented increased G3BP1 protein levels (*p* < 0.05) and decreased YBX1 protein levels (*p* < 0.05). As shown in Figure 6F and Figure S13C (Supporting Information), the immunofluorescence results revealed that G3BP1 was expressed in the cytoplasm, and the fluorescence intensity of the proteins in each group was consistent with the western blot results.

As shown in Figure 6G–I,K, compared with those of the NC group, the cell viability of the HH/R model group was significantly lower (*p* < 0.01), and LDH, Fe^2+^, and p53^N/T^ levels were significantly greater (*p* < 0.01). Compared with those of the HH/R model group, the cell viability of the HH/R+LV‐G3BP1 group was significantly greater (*p* < 0.01), and the levels of LDH, Fe^2+^ and p53^N/T^ were significantly lower (*p* < 0.01). Compared with those of the HH/R model group, the cell viability of the HH/R+siYBX1 group was also increased (*p* < 0.01), and LDH, Fe^2+^, and p53^N/T^ levels decreased (*p* < 0.01 or 0.05). Compared with those of the HH/R+LV‐G3BP1 or HH/R+siYBX1 group, the cell viability of the HH/R+LV‐G3BP1+siYBX1 group was further greater (*p* < 0.01), and LDH, Fe^2+^, and p53^N/T^ levels were significantly lower (*p* < 0.01).

As shown in Figure 6J, the ultrastructure of the H9c2 cells in the NC group was intact, and the continuity of the mitochondrial cristae was not damaged. The mitochondrial structure was destroyed, and the continuity of the mitochondrial cristae was disrupted in the HH/R group of cells. After LV‐G3BP1 treatment, the damage to the mitochondrial structure and disruption of mitochondrial cristae were alleviated. After siYBX1 intervention, the degree of mitochondrial damage in the cells was also alleviated compared with that in the HH/R group. Compared with that in the HH/R+siYBX1 group, the degree of mitochondrial damage in the HH/R+LV‐G3BP1+siYBX1 group was furtherly alleviated. As shown in Figure 6L–N, compared with those in the NC group, the protein level of YBX1 was greater in the HH/R model group (*p* < 0.01); SLC7A11 and GPX4 protein levels and GPX4 mRNA levels were significantly lower (*p* < 0.01). Compared with those in the HH/R model group, the protein levels of YBX1 in the HH/R+LV‐G3BP1 group were lower (*p* < 0.01), and SLC7A11 and GPX4 protein levels and GPX4 mRNA levels were significantly greater (*p* < 0.05). Compared with those in the HH/R model group, the protein levels of YBX1 in the HH/R+siYBX1 group were lower (*p* < 0.01), and SLC7A11 and GPX4 protein levels and GPX4 mRNA levels were greater (*p* < 0.01 or 0.05). Compared with those in the HH/R+LV‐G3BP1 group, the protein levels of YBX1 in the HH/R+LV‐G3BP1+siYBX1 group were lower (*p* < 0.01), and SLC7A11 and GPX4 protein levels and GPX4 mRNA levels were greater (*p* < 0.01). Compared with those in the HH/R+siYBX1 group, the protein levels of YBX1 in the HH/R+LV‐G3BP1+siYBX1 group were lower (*p* < 0.01), and SLC7A11 and GPX4 protein levels and GPX4 mRNA levels were greater (*p* < 0.01 or 0.05).

### 3.7 Interaction Mechanisms Between G3BP1 and YBX1, YBX1 and p53 and G3BP1 and p53

**Figure 7:**
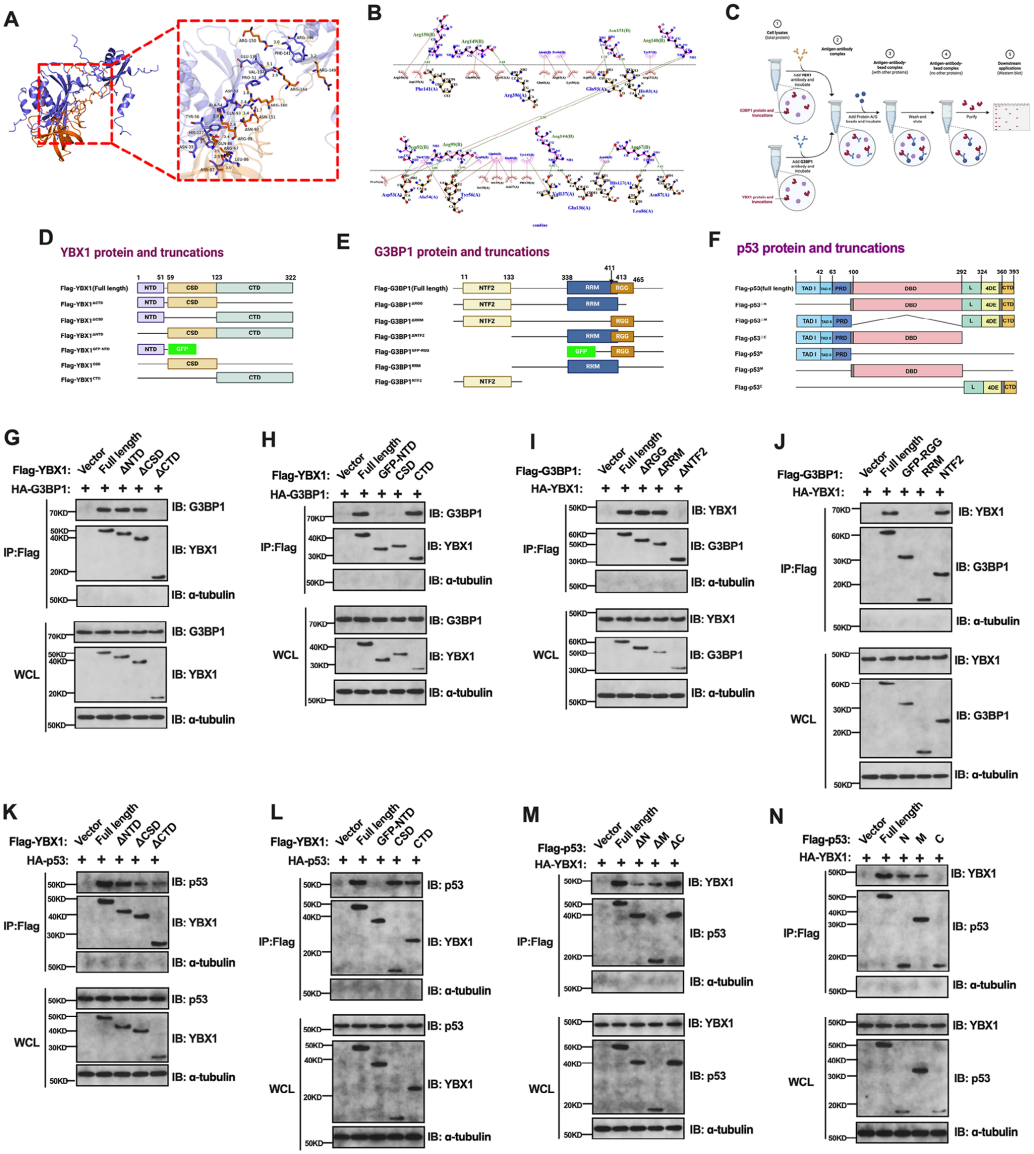
Interaction mechanism between G3BP1 and YBX1 and between YBX1 and p53. A) 3D structure of the interaction between G3BP1 and YBX1 protein. B) The binding domain between G3BP1 and YBX1. C) The experimental procedure of CO‐IP to detect the interaction between G3BP1 protein and YBX1 protein. D) Construct truncated fragments targeting different domains of YBX1 protein. E) Construct truncated fragments targeting different domains of G3BP1 protein. F) Construction of truncates targeting different domains of p53 protein. G,H) CO‐IP was used to detect the YBX1 domain that bound to G3BP1 protein. I,J) CO‐IP was used to detect the G3BP1 domain that bound to YBX1 protein. K,L) CO‐IP was used to detect the YBX1 protein domain that interacted with p53 protein. M,N) CO‐IP was used to detect the p53 protein domain interacting with YBX1 protein.

As shown in **Figure** **7**A,B, we retrieved the RAT‐G3BP1 protein (https://www.uniprot.org/uniprotkb/D3ZYS7/entry#structure) and the RAT‐YBX1 protein (https://www.uniprot.org/uniprotkb/P6 2961/entry#structure). Most of the amino acid structure of G3BP1 bound to YBX1 was predicted to be in the NTF2 domain, and only one amino acid structure was found to be located in the RRM domain via molecular docking. YBX1 bound to G3BP1 via three amino acids in the CSD region and five amino acids in the CTD region.

Figure 7C demonstrates the experimental procedure using co‐immunoprecipitation (CO‐IP) to validate protein–protein interactions. As shown in Figure 7D, YBX1 contains three conserved domains: the N‐terminal domain (NTD), the CSD, and the C‐terminal domain (CTD). On this basis, we constructed the following YBX1s with FLAG tags: FLAG‐YBX1 (full length), deletion NTD (FLAG‐YbX1^ΔNTD^), deletion CSD (FLAG‐YbX1^ΔCSD^), deletion CTD (FLAG‐YBX1^ΔCTD^), NTD motif (FLAG‐YBX1^GFP‐NTD^), CSD domain (FLAG‐YBX1^CSD^), and NTF2 domain (FLAG‐YBX1^CTD^). In addition to the FLAG tag, a GFP tag was added to the NTD motif because of its short length. The HA‐tagged G3BP1 plasmid was used to overexpress H9c2 cells together with FLAG‐tagged full‐length YBX1 and various truncated plasmids in H9c2 cells, and then, M2 beads were used for immunoprecipitation of different truncated YBX1 regions to determine the binding domain of YBX1 to G3BP1. As shown in Figure 7G, we found that full‐length YBX1 with deletion of NTD (ΔNTD) and deletion of CSD (ΔCSD) could be enriched with HA‐G3BP1. When YBX1 was deleted with a CTD (ΔCTD), the FLAG tag enriched with HA‐G3BP1 was not detected. These findings suggest that the region that interacts with G3BP1 may be located in the CTD region of YBX1. As shown in Figure 7H, to analyze the interaction between the two proteins more directly, we incubated the purified recombinant G3BP1 protein in vitro, the full‐length YBX1 with a FLAG tag, and the recombinant NTD, CSD, and CTD proteins. NTD, CSD and CTD were also enriched using M2 beads. Owing to the short motif of the NTD, we added a GFP tag to its N‐terminus in addition to the FLAG tag. Similar results were obtained. The full‐length YBX1 and CTD were enriched with the G3BP1 recombinant protein, but the NTD and CSD were not enriched. These findings suggest that the region that interacts with G3BP1 may be in the CTD region of YBX1.

As shown in Figure 7E, G3BP1 includes the NTF2 (nuclear transporter factor 2) domain, RRM (RNA‐recognition module) domain and RGG (arginine–glycine–glycine) motif. As shown in Figure 7I, we found that full‐length G3BP1 with RGG deletion (ΔRGG) and RRM deletion (ΔRRM) could be enriched with HA‐YBX1. However, the FLAG tag enriched with HA‐YBX1 was not detected in G3BP1 with NTF2 deletion (ΔNTF2). These findings suggest that the region that interacts with YBX1 may be in the NTF2 region of G3BP1. As shown in Figure 7J, to analyze the interaction between the two proteins more directly, we incubated the purified YBX1 recombinant protein, the full‐length G3BP1 with a FLAG tag, and the recombinant NTF2, RGG, and RRM proteins. NTF2, RGG and RRM were also enriched using M2 beads. Owing to the short motif of RGG, we added a GFP tag to its N‐terminus in addition to the FLAG tag. Similar results were obtained. Full‐length G3BP1 and NTF2 were enriched in the YBX1 recombinant protein, whereas RGG and RRM were not enriched. These findings suggest that the region that interacts with YBX1 may be in the NTF2 region of G3BP1.

**Figure 8:**
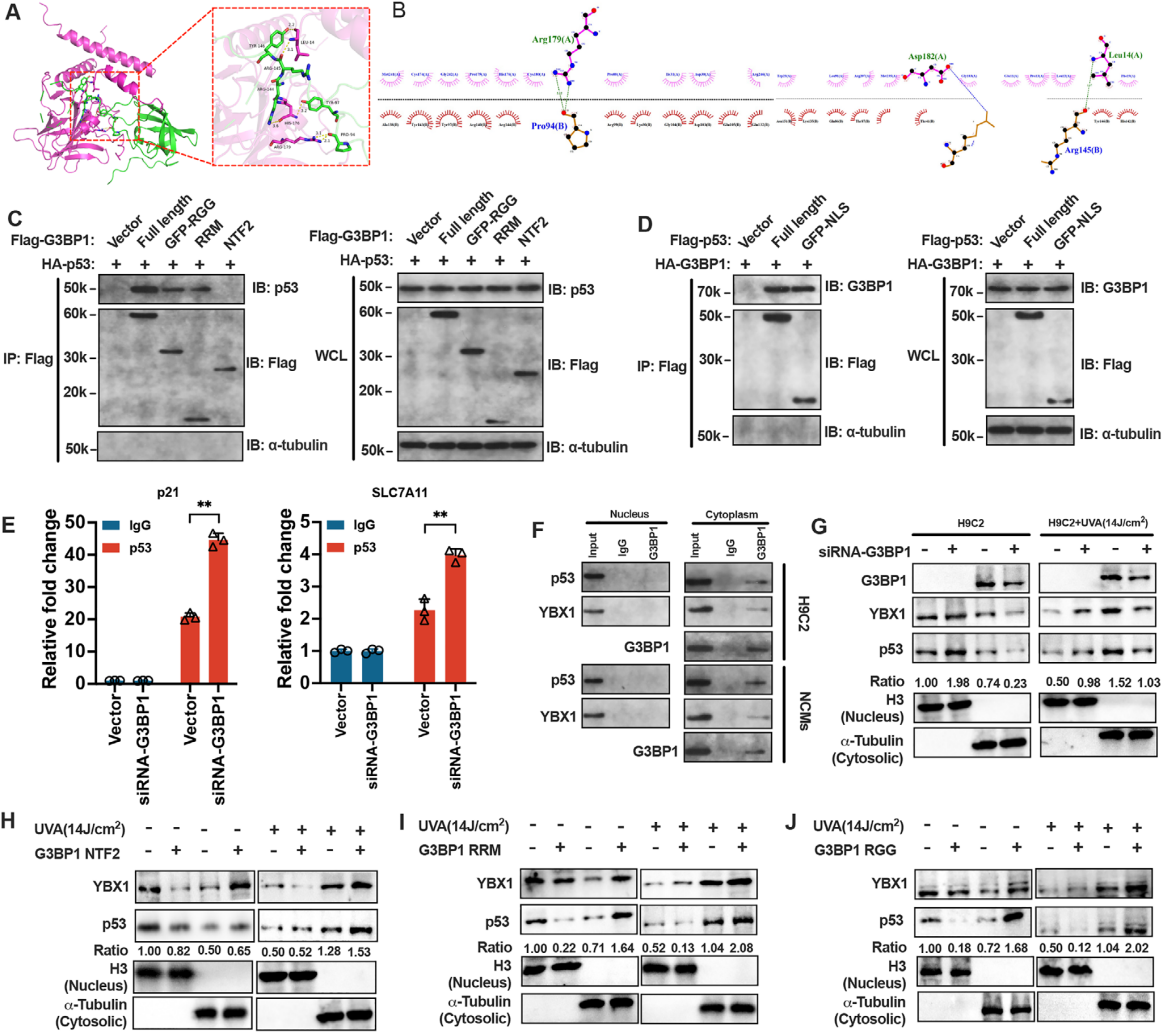
Interaction mechanism between G3BP1 and p53 and the effect of G3BP1 on the distribution of YBX1 and p53 in the nucleus and cytoplasm. A,B) Predicted 3D structure and binding domains of YBX1‐p53 interaction. C,D) Co‐IP analysis of G3BP1 domains (NTF2/RRM/RGG) and p53 domains (N/M/C) in H9c2 cells transfected with HA‐G3BP1/FLAG‐p53 plasmids (48 h). E) ChIP‐qPCR for p53 binding to p21 (positive control) and SLC7A11 promoters in siG3BP1 versus siCtrl cells (72 h post‐transfection). F) CO‐IP was used to detect the binding of YBX1, p53, and G3BP1 proteins in the cytoplasm and nucleus from HH/R‐treated cells (6 h hypoxia/24 h reoxygenation). G–J) western blot was used to detect the expression of G3BP1, YBX1, and p53 proteins in the cytoplasm and nucleus. The data was expressed as means ± SD. *n* = 3. Normality was confirmed using the Shapiro–Wilk test. Two‐group comparisons were analyzed by Student's *t*‐test. ^**^ *p* < 0.01, ##*p* < 0.01.

**Figure 9:**
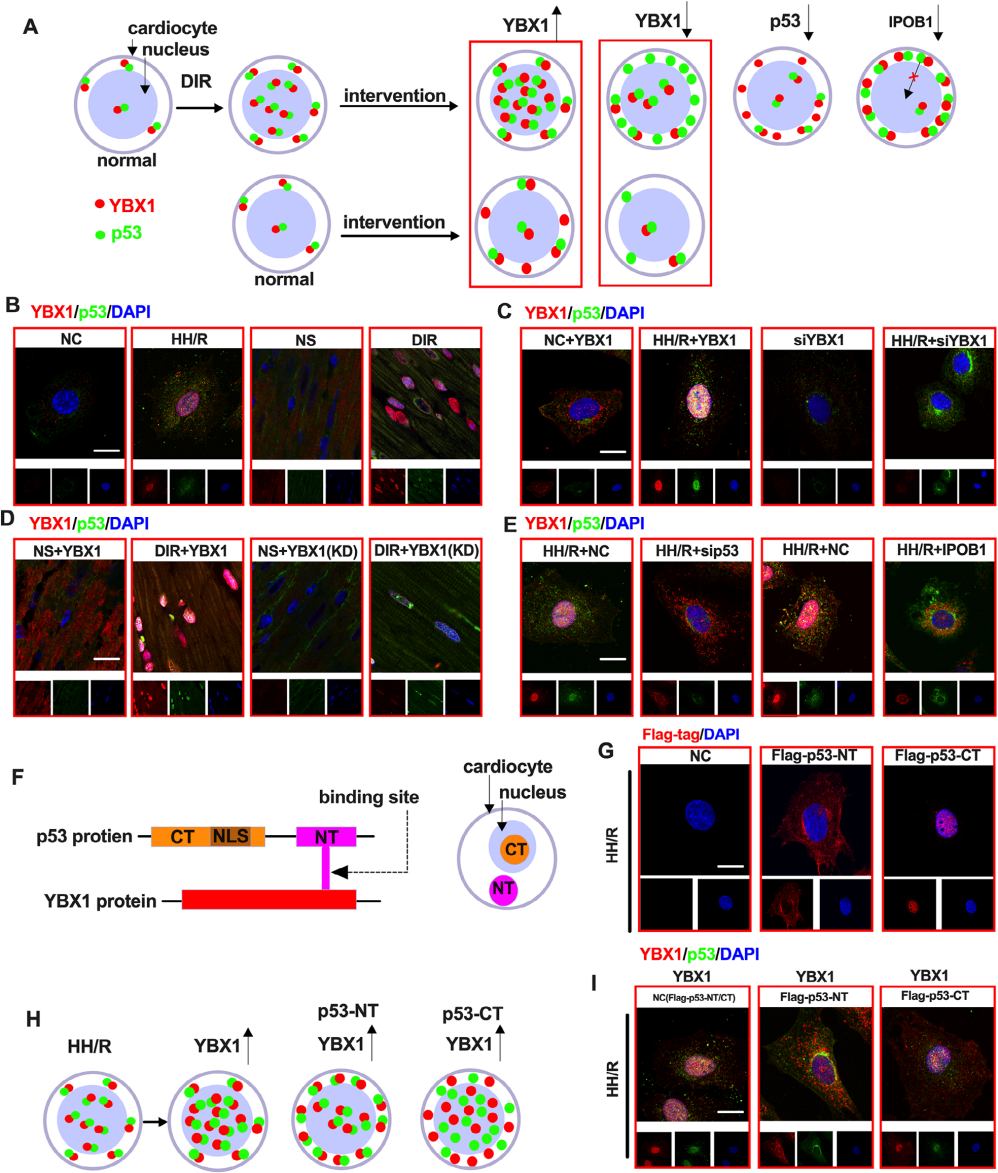
Interdependent nuclear translocation of YBX1 and p53 aggravates myocardial ischemia‐reperfusion injury in diabetic hearts. A) Schematic illustration depicting the nuclear translocation of YBX1 (red dots) and p53 (green dots) in cardiomyocytes under normal and DIR conditions, and the effect of intervention on their distribution. B–D) IF images showing the expression of YBX1 (red) and p53 (green) proteins in myocardial tissue. E) IF images in cardiomyocytes showing the expression of YBX1 and p53 proteins under different treatment conditions. F) Schematic diagram indicating the binding site between p53 protein (with C‐terminal (CT), Nuclear Localization Signal (NLS), and N‐terminal (NT)) and YBX1 protein. G) IF images in cardiomyocytes showing the expression of Flag – tagged p53 – NT (N – terminal) and Flag‐tagged p53‐CT (C‐terminal) under NC and HH/R conditions. H) Schematic representation of the effect of HH/R on the levels of YBX1, p53‐NT, and p53‐CT in cardiomyocytes. I) IF images in cardiomyocytes showing the co‐localization of YBX1 with Flag – tagged p53 – NT and Flag – tagged p53 – CT under HH/R conditions. The scale bars in Figure B,C,D,E,G, and I represent 20 µm.

As shown in Figure 7F, p53 is divided into three domains: N (including the TAD I, TAD II, and PRD domains), M (including the DBD domains), and C (including the L, 4DE, and CTD domains). As shown in **Figure** **8**A,B, we retrieved the RAT‐p53 protein structure (https://www.uniprot.org/uniprotkb/Q95330/entry), and YBX1 was predicted by molecular docking and p53 in combination with an amino acid structure in the CSD domain structure, with two amino acid structures in the CTD domain. p53 bound YBX1 with one amino acid structure in the N region and two amino acid structures in the M region. To analyze the interaction between YBX1 and p53 more directly, we first constructed different YBX1 domains with FLAG tags, as shown in Figure 7D. The HA‐tagged p53 plasmid was used to overexpress H9c2 cells together with FLAG‐tagged full‐length YBX1 and various truncated YBX1 plasmids, and then, M2 beads were used for immunoprecipitation of different truncated YBX1 plasmids. As shown in **Figure** **9**K, we found that full‐length YBX1 with deletion of the NTD (ΔNTD) could be enriched with HA‐p53. However, deletion of CSD (ΔCSD) and CTD (ΔCTD) in YBX1 resulted in a reduced FLAG tag enriched with HA‐p53. As shown in Figure 7L, to analyze the interaction between the two proteins more directly, we incubated the in vitro purified recombinant p53 protein, the full‐length YBX1 with a FLAG tag, and the recombinant NTD, CSD and CTD proteins. NTD, CSD and CTD were also enriched using M2 beads. Owing to the short motif of the NTD, we added a GFP tag to its N‐terminus in addition to the FLAG tag. Similar results were obtained: full‐length p53, CSD and CTD were enriched in the p53 recombinant protein, but NTD was not enriched in the p53 recombinant protein. These findings suggest that the regions that interact with p53 may be in the CSD and CTD regions of YBX1.

We next analyzed which domain of p53 binds YBX1 to each other. As shown in Figure 7M, we found that HA‐YBX1 could be enriched by full‐length, N (ΔC)‐deleted p53. However, deletion of N (ΔN) and M (ΔM) p53 resulted in a reduction in the FLAG tag enriched with HA‐YBX1. As shown in Figure 7N, to analyze the interaction between the two proteins more directly, we incubated the purified recombinant p53 protein in vitro, the full‐length p53 with a FLAG tag, and the N, M, and C recombinant proteins. N, M, and C were also enriched via M2 beads. Similar results were obtained. Full‐length p53, N and M were enriched in the YBX1 recombinant protein, but C was not enriched in the YBX1 recombinant protein. These findings suggest that the region that interacts with YBX1 may be in the N and M regions of p53.

In previous studies, we reported that the NTF2 and RRM domains of the G3BP1 protein can bind to the nuclear localization sequence (NLS) of p53.^[^ ^16^ ^]^ Therefore, this study further verified this conclusion in H9c2 cells. As shown in Figure 8C, we incubated the recombinant p53 protein purified in vitro with the full‐length FLAG‐tagged G3BP1 and the NTF2, RGG, and RRM recombinant proteins. NTF2, RGG, and RRM were also enriched using M2 beads. We found that full‐length G3BP1, RGG and RRM were enriched in the p53 recombinant protein, but NTF2 was not enriched in the p53 recombinant protein. These findings suggest that the region that interacts with p53 may be in the RGG and RRM regions of G3BP1. As shown in Figure 8D, in vitro purified recombinant G3BP1 protein and FLAG‐tagged full‐length p53 and NLS histone proteins were incubated. The NLS was also enriched using M2 beads. We found that both full‐length p53 and the NLS could enrich the recombinant G3BP1 protein, suggesting that the NLS region of p53 may interact with G3BP1.

### 3.8 G3BP1 Regulates the Nuclear Translocation of YBX1 to p53

In the above studies, we found that G3BP1 could interact with YBX1, YBX1 could interact with p53, and G3BP1 could interact with p53. However, G3BP1 was located mainly in the cytoplasm, and the YBX1 and p53 proteins were expressed in both the cytoplasm and nucleus. Therefore, this study partially investigated the effects of G3BP1 on YBX1 and the p53 nuclear import. As shown in Figure 8E, a ChIP assay revealed that p53 recruitment to the ALC7A11 promoter region increased after G3BP1 was knocked down in H9c2 cells (*p* < 0.01). Among them, p21 was used as a positive control. As shown in Figure 8F, the co‐IP results revealed that the G3BP1 protein could interact with the p53 and YBX1 proteins in the cytoplasm of H9c2 and NMVC cells. However, G3BP1, p53 and YBX1 did not bind to each other in the nucleus. As shown in Figure 8G and Figure S13D (Supporting Information), since the expression of G3BP1 sequesters p53 and YBX1 proteins in the cytoplasm, we found that the knockdown of G3BP1 in H9c2 cells reduced the levels of the p53 and YBX1 proteins in the cytoplasm and increased the levels of the p53 and YBX1 proteins in the nucleus. UVA (14 J cm^−2^) induced p53 and YBX1 to enter the cytoplasm, but knockdown of G3BP1 increased the expression of the p53 and YBX1 proteins in the nucleus and decreased the expression of the p53 and YBX1 proteins in the cytoplasm.

The effects of the G3BP1 domain on the protein expression of p53 and YBX1 in the cytoplasm and nucleus were examined under UVA irradiation and nonirradiated conditions. As shown in Figure 8H, the NTF2 domain of G3BP1 reduced YBX1 protein expression in the nucleus and increased YBX1 protein expression in the cytoplasm. The NTF2 domain had little effect on p53 protein expression in the nucleus or cytoplasm. Similarly, the NTF2 domain of G3BP1 induced only YBX1 expression in the cytoplasm under UVA treatment. As shown in Figure 8I, the RRM domain of G3BP1 reduced the expression of the p53 protein in the nucleus and increased the expression of the p53 protein in the cytoplasm. The NTF2 domain had little effect on the expression of the YBX1 protein in the nucleus or cytoplasm. Similarly, the RRM domain of G3BP1 only increased p53 expression in the cytoplasm under UVA treatment. As shown in Figure 8J, the RGG domain of G3BP1 simultaneously reduced the expressions of the YBX1 and p53 proteins in the nucleus and increased the expressions of the YBX1 and p53 proteins in the cytoplasm. Similarly, the RRM domain of G3BP1 further increased YBX1 and p53 expression in the cytoplasm under UVA treatment. These findings suggest that the NTF2 and RGG domains of G3BP1 can block the cytoplasmic expression of YBX1 and that the RRM and RGG domains can block the cytoplasmic expression of p53.

### 3.9 Interdependent Nuclear Translocation of YBX1 and p53 Exacerbates Myocardial Ischemia‒Reperfusion Injury in Diabetic Hearts

We further clarified whether interfering with the expression of YBX1 or the nuclear translocation of YBX1 and p53 is an important therapeutic strategy and molecular target for cardiac ischemia‒reperfusion injury. Figure 9A shows the intracellular expression and distribution of YBX1 and p53 proteins in the normal, model, and intervention groups.

As shown in Figure 9B and Figure S11A–H (Supporting Information), YBX1 and p53 were found to be colocalized in the cytoplasm in H9c2 cells and normal cardiac tissue under normoxic conditions. Under conditions of HH/R in cells and DIR in tissues, the combination was enhanced and transferred to the nucleus (*p* < 0.01).

As shown in Figure 9C and Figure S11I–L (Supporting Information), knockdown or overexpression of YBX1 had no effect on the expression or distribution of p53 in H9c2 cells under normoxic conditions. When the expression of YBX1 was knocked down, the expression of p53 in the nucleus decreased (*p* < 0.01). The expression of p53 in the HH/R group was not significantly greater than that in the HH/R group. As shown in Figure 9D and Figure S8M–P (Supporting Information), when knockdown or overexpression of YBX1 in the myocardial tissue, the p53 and YBX1 showed same trend as that observed in the cell experiments.

As shown in Figure 9E and Figure S11Q (Supporting Information), p53 knockdown under H/R conditions reduced YBX1 nuclear translocation (*p* < 0.01). As shown in Figure 9E and Figure S11R,S (Supporting Information), importin‐β1 is a classical protein that promotes p53 nuclear translocation. Importin‐β1 knockdown also reduced the level of YBX1 nuclear translocation (*p* < 0.01), indicating that p53 is required for YBX1 nuclear translocation.

As shown in Figure 9F, two p53 fragments were subsequently constructed, with an N‐terminal fragment (p53‐NT) containing the YBX1 binding domain without an NLS. The C‐terminal fragment (p53‐CT) contained a p53‐associated NLS. As shown in Figure 9G, the immunofluorescence results revealed that FLAG‐p53‐NT was distributed in the cytoplasm of H9c2 cells under HH/R conditions, whereas FLAG‐p53‐CT was distributed in the nucleus. As shown in Figure 9H–I, FLAG‐p53‐NT and endogenous p53 competitively bind YBX1; however, since FLAG‐p53‐NT does not have an NLS, both YBX1 and p53 are expressed mainly in the cytoplasm, so FLAG‐p53‐NT inhibits YBX1 nuclear translocation. However, FLAG‐p53‐CT could not bind to YBX1 and contained a p53‐associated NLS. Therefore, p53 was expressed mainly in the nucleus without affecting the nuclear translocation of YBX1. While initial candidate selection relied on bioinformatics, empirical validation via Co‐IP and functional assays (Figures 7, 8, 9) confirmed YBX1 as a key mediator. As shown in Figure S14 (Supporting Information), ALKBH5 could influence the level of G3BP1, YBX1, and p53. However, ALKBH5 overexpression only significantly increased the m^6^A level of G3BP1, with no obvious effect on the m^6^A levels of YBX1 and p53. Combined with the results from MeRIP‐seq and RNA‐seq in Figure 4, which showed that ALKBH5 does not affect the RNA methylation levels of YBX1 and p53, we conclude that ALKBH5 may indirectly regulate the G3BP1‐YBX1‐p53 axis by influencing the m6A level of G3BP1.

As shown in Figure S12A–D, YBX1 overexpression increased the HH/R‐induced apoptosis rate (*p* < 0.01), and the HH/R‐induced apoptosis rate decreased after YBX1 was knocked down (*p* < 0.01). There was potentially dependent accumulation of JC‐1 dye within the mitochondria. When the mitochondrial membrane potential was high, JC‐1 aggregated in the mitochondrial matrix to form aggregates, which emitted intense red fluorescence. When the mitochondrial membrane potential decreased, JC‐1 could not aggregate and was in the monomer form, emitting green fluorescence. When cell damage occurs, the mitochondrial membrane potential decreases, and the aggregation/monomer ratio decreases. YBX1 overexpression further reduced the HH/R‐induced aggregation/monomer ratio (*p* < 0.01), whereas the ratio was increased by the knockdown of YBX1 (*p* < 0.01). As shown in Figure S12E (Supporting Information), TUNEL staining revealed that p53 knockdown reversed YBX1‐induced apoptosis (*p* < 0.01).

## 4 Discussion

Diabetic ischemic cardiomyopathy (DICM) poses a growing clinical challenge, with myocardial ischemia‐reperfusion (I/R) injury exacerbating oxidative stress and ferroptosis—a programmed cell death driven by lipid peroxidation and glutathione depletion.^[^ ^21^, ^22^ ^]^ Central to ferroptosis is SLC7A11, which sustains glutathione synthesis via cystine uptake; its suppression disrupts GPX4 activity, amplifying reactive oxygen species (ROS) and mitochondrial damage.^[^ ^23^, ^24^ ^]^ Diabetic patients exhibit heightened ferroptosis susceptibility due to iron dysregulation and oxidative imbalance,^[^ ^25^ ^]^ yet the role of RNA methylation in this context remains unclear. Our study uncovers a critical role for the RNA demethylase ALKBH5 in regulating ferroptosis through the G3BP1‐YBX1‐p53 axis in cardiomyocyte and fibroblast (Figure S15, Supporting Information), offering both mechanistic insights and translational opportunities. Moreover, diabetes‐specific factors aggravated myocardial I/R injury and ferroptosis, influenced the ALKBH5‐G3BP1‐YBX1/p53 axis (Figure S16, Supporting Information).

ALKBH5 holds translational potential as a therapeutic target. The identification of ALKBH5 as a regulator of ferroptosis in diabetic myocardial injury highlights its clinical biomarker and therapeutic potential. Clinical data from 15 DM patients and 8 non‐DM controls revealed inverse correlations between ALKBH5 expression and cardiac injury markers (BNP, cTnl), suggesting ALKBH5 levels could serve as a diagnostic indicator for DICM severity. In preclinical models, AAV‐mediated ALKBH5 overexpression reduced infarct size by 42% in DIR rats (Figure 2F), outperforming nonspecific ferroptosis inhibitors (e.g., Ferrostatin‐1) and antioxidants. This specificity arises from ALKBH5's targeted regulation of the m⁶A‐G3BP1‐p53 axis, which avoids the broad systemic effects of traditional therapies.^[^ ^23^, ^24^ ^]^

Compared to gene therapies like SIRT3 overexpression, our strategy uniquely combines ferroptosis inhibition with cytoplasmic sequestration of pro‐apoptotic YBX1/p53, potentially promoting dual protective‐reparative effects. The use of viral vectors (AAV) ensures sustained transgene expression, aligning with prior studies on viral delivery in myocardial injury.^[^ ^26^ ^]^ However, translating this to humans requires addressing vector safety (e.g., immune responses, insertional mutagenesis) and optimizing delivery to cardiac tissue. Nanocarriers (e.g., pH‐responsive lipid nanoparticles), which offer reduced immunogenicity and targeted delivery, represent a promising alternative for future clinical development.^[^ ^26^ ^]^

The study establishes a novel epigenetic mechanism linking RNA methylation to ferroptosis in diabetes. ALKBH5‐mediated demethylation of G3BP1's 3′‐UTR (sites 142/173) stabilizes G3BP1 mRNA, preventing nuclear translocation of the YBX1‐p53 complex. This mechanism diverges from G3BP1's role in stress granules (SGs) by specifically integrating m⁶A modification with protein‐protein interactions. Unlike previous SGs‐focused studies,^[^ ^20^, ^27^, ^28^, ^29^, ^30^, ^31^ ^]^ our work identifies a diabetes‐specific axis where ALKBH5‐G3BP1 blocks p53‐dependent repression of SLC7A11, thereby preserving glutathione synthesis and GPX4 activity.

The interplay between ALKBH5 and the m⁶A methyltransferase METTL3/FTO further underscores the dynamic nature of RNA methylation in ferroptosis: while ALKBH5 and FTO (another demethylase) both inhibit ferroptosis, ALKBH5 exhibits stronger efficacy, likely due to its preferential targeting of G3BP1.^[^ ^6^, ^25^ ^]^ These findings expand our understanding of epigenetic regulation in diabetic complications, positioning ALKBH5 as a central node in a network linking RNA stability, subcellular signaling, and cell fate.

For the clinical challenges and optimization pathways, while AAV‐mediated ALKBH5 delivery shows promise, its translational trajectory requires addressing two key barriers: 1) Immune risks from viral vectors, as pre‐existing immunity to AAV serotypes in 30–60% of humans may limit efficacy,^[^ ^26^ ^]^ and 2) Potential oncogenicity from sustained p53 cytoplasmic retention, despite our DIR model showing no tumorigenesis at 12‐week follow‐up. To mitigate these, we propose hybrid delivery systems combining nanocarriers (e.g., cardiac‐targeted lipid nanoparticles from reference)^[^ ^26^ ^]^ with transient ALKBH5 expression—a strategy that could leverage the cardiomyocyte specificity of AAV while reducing viral load. Furthermore, the partial ferroptosis rescue in G3BP1‐mutant models (Figure 4D) suggests adjuvant therapies targeting parallel nodes (e.g., YBX1 inhibitors) may be needed for patients with ALKBH5 polymorphisms—a hypothesis testable through pharmacogenomic stratification in upcoming trials.

For the limitations and future directions, despite these advances, several challenges limit immediate clinical translation. First, model limitations: while DIR rats mimic human DICM in ALKBH5 downregulation and ferroptosis,^[^ ^25^ ^]^ they lack the complexity of human diabetes comorbidities (e.g., obesity, hypertension). Longitudinal studies are needed to validate ALKBH5's role in chronic DICM progression. Second, vector‐related risks: AAV‐mediated overexpression requires careful dosing to avoid off‐target effects, and lentiviral integration risks necessitate non‐viral alternatives.^[^ ^26^ ^]^ Third, compensatory pathways: partial rescue of ferroptosis after G3BP1 m⁶A site mutagenesis suggests additional regulators (e.g., other m⁶A‐modified genes in the RNA‐seq/MeRIP‐seq dataset), which warrant further investigation.

Future studies should prioritize: clinical validation via prospective trials correlating ALKBH5 levels with DICM prognosis and treatment response; delivery optimization through developing cardiac‐targeted nanocarriers to enhance ALKBH5 specificity and reduce systemic exposure; and combination therapies by pairing ALKBH5 with iron chelators or SGs modulators to address multi‐pathway dysregulation in diabetes.

**Figure 10:**
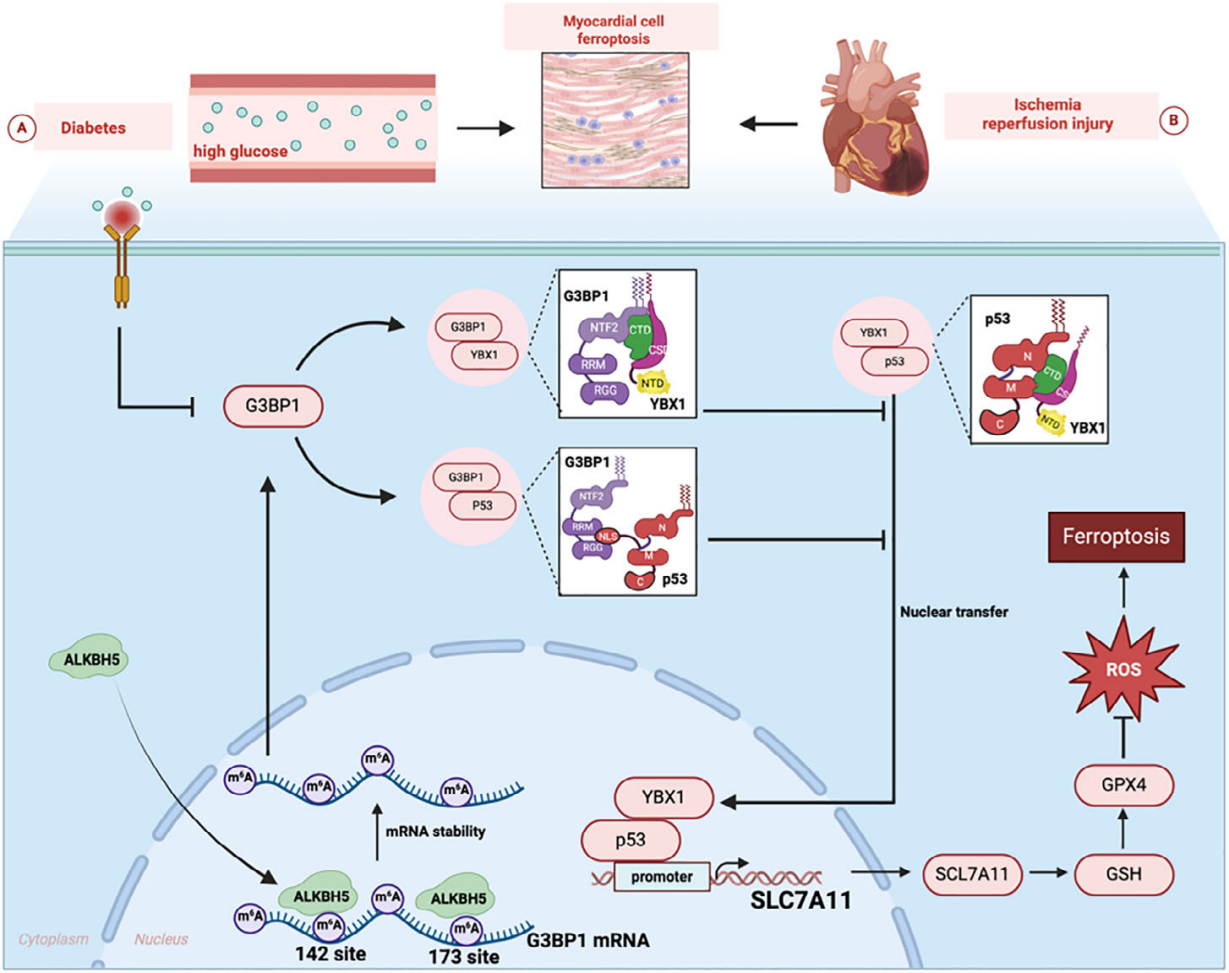
Pattern diagram of ALKBH5 regulating G3BP1 through m^6^A modification affecting cardiomyocyte ferroptosis. In the process of diabetic myocardial ischemia‐reperfusion, the protein level of G3BP1 is decreased, and the amount of binding p53 and YBX1 proteins is decreased. After p53 translocation into the nucleus, it binds to the promoter region of SLC7A11 gene, the transcription level of SLC7A11 is decreased, the expression of GSH and GPX4 is decreased, and then the level of ROS‐mediated ferroptosis is increased. ALKBH5 promoted the expression of G3BP1 by regulating the 142 and 173 m^6^A methylation sites on G3BP1 mRNA. Subsequently, G3BP1 binds to the CTD domain of YBX1 through the NTF2 domain and the NLS domain of p53 through the RGG and RRM domains, thereby inhibiting p53 and YBX1 nuclear translocation. p53 and YBX1 can synergistically enter the nucleus to aggravate myocardial cell injury. Therefore, overexpression of G3BP1 can reduce the nuclear translocation of p53 by capturing YBX1 and p53, which increases the transcription level of SLC7A11 and the expression of GSH and GPX4 and subsequently inhibits ROS‐mediated ferroptosis of cardiomyocytes.

In conclusion, this study bridges RNA epigenetics and cardiac biology, demonstrating that ALKBH5 safeguards against diabetic myocardial I/R injury by stabilizing G3BP1 and disrupting the YBX1‐p53 pro‐ferroptotic axis (**Figure** **10**). While challenges in translation persist, the specificity of this epigenetic mechanism offers a promising avenue for precision medicine in DICM. By integrating mechanistic discovery with translational strategy, our findings lay the groundwork for developing ALKBH5‐based diagnostics and therapies, with broader implications for RNA methylation‐targeted interventions in neurodegenerative and metabolic diseases.

## Conflict of Interest

The authors declare no conflict of interest.

## Author Contributions

W. L., W. L. and Y. L. contributed equally to this article. Y.W. and Z.X. takes responsibility for the integrity of the work as a whole, from inception to published article. Y.W., Z.X., and W.L. conceived and designed the study. W.L., W.L., Y.L, and H.X. perform the experiment. Y.W., Z.X., W.L. and Y.L. wrote the paper. Y.W. and Z.X. edited the article. All authors approved the final version of the manuscript.

## Supporting information



Supporting Information

## Data Availability

The data that support the findings of this study are available from the corresponding author upon reasonable request.
