# Supplementary material for: ALKBH5‐Mediated M6A Demethylation of G3BP1 Attenuates Ferroptosis Via Cytoplasmic Retention of YBX1/p53 in Diabetic Myocardial Ischemia‐Reperfusion Injury
Source: Adv Sci (Weinh). 2025 Jun 23;12(35):e07254. doi: 10.1002/advs.202507254 (PMC12463097; doi:10.1002/advs.202507254)
Supplement: Supplementary file 1 — Supporting Information [file ADVS-12-e07254-s001.docx]

**Figure S1 Establishment and verification of the diabetic myocardial ischemia‒reperfusion injury animal model**

We first examined the effects of DM on the heart in animal experiments. As shown in Fig. S1A–H, compared with those in the nondiabetic group, the levels of blood glucose, water intake, heart weight/body weight, LVIDs and LVIDd in the diabetic group were significantly greater (*P*<0.01), and the body weight, heart rate and LVEF were significantly lower (*P*<0.01). The echocardiographic results in Fig. S1I show that the systolic function of the heart was significantly impaired in diabetic rats compared with nondiabetic rats. Fig. S1J Speckle imaging results showed normal coronary flow and filling of the myocardial tissue in the myocardial supply territory of the left coronary artery prior to myocardial ischemia. After 30 min of ischemia, myocardial tissue blood flow in the myocardial supply territory of the left coronary artery was significantly reduced. After 120 min of reperfusion, myocardial tissue blood flow in the myocardial supply territory of the left coronary artery was restored, but it was less than that before ischemia.

As shown in Fig. S1K–M, a laser Doppler flowmeter (PeriFlux 600) was used to monitor the blood flow in the heart in real time and to monitor blood perfusion (PU) and the erythrocyte migration rate (ESR) simultaneously (VU). PU and VU values were significantly lower in the myocardium than they were before ischemia and significantly lower after 30 min of myocardial ischemia. The PU and VU values increased immediately after myocardial ischemia‒reperfusion. PU and VU decreased after 120 min of reperfusion. As shown in the ECG results in Fig. S1N, the ST segment was at baseline during pre-ischemia. When the LAD artery was ligated, the ST segment was elevated, and the QRS wave was widened. At 30 min of ischemia, the ST segment elevation and QRS wave significantly widened. Immediately after reperfusion, the ST segment retreated, and the wide QRS complex became narrower than it was during ischemia. At 60 min of reperfusion, the ST segment decreased significantly, and the QRS wave narrowed. At 120 min of reperfusion, the ST segment returned to the baseline level, and the wide QRS wave further narrowed. However, it was wider than that before ischemia, and the heart rate was significantly lower than that before ischemia. These results indicated that the diabetic model was successfully constructed and that the pumping function of the heart was affected. In the context of diabetes mellitus, the myocardial blood supply, blood perfusion and cardiac electrophysiology in the area dominated by the left coronary artery changed significantly, which indicated that the diabetic myocardial ischemia‒reperfusion injury model was successfully constructed.

**
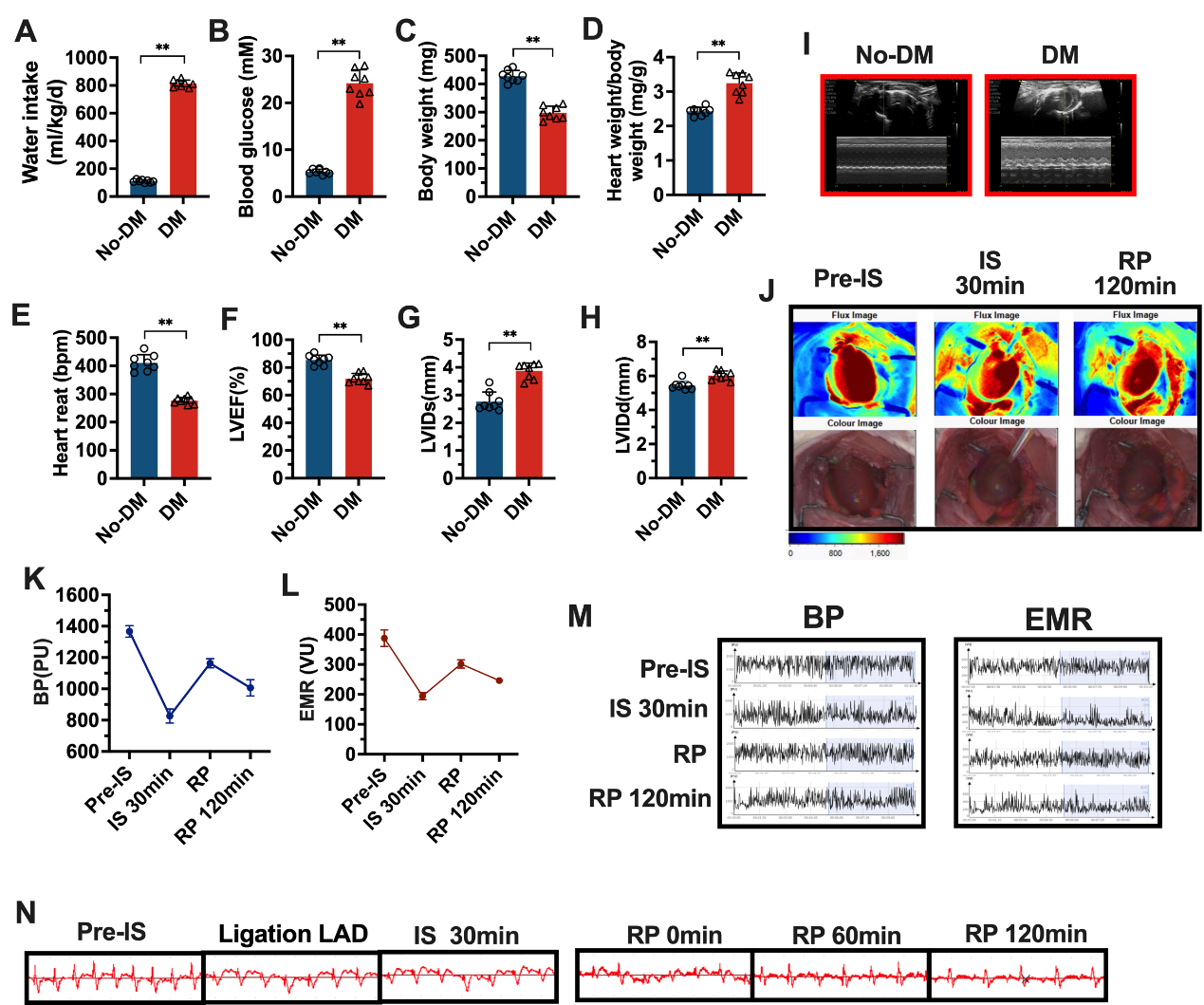
**

**Figure S1 legend** Construction and validation of an animal model of diabetic myocardial ischemia-reperfusion injury. (A-H) The changes of water intake, blood glucose, body weight, heart weight/body weight, hear rate, LVEF, LVIDs, and LVIDd levelsin No-DM and DM group. (I) Cardiac systolic function was detected by echocardiography. (J) Speckle imaging was used to detect the changes of cardiac blood perfusion in rats. (K-M) The changes of PU and VU were measured by laser Doppler flowmeter (PeriFlux 600). (P) Electrocardiogram was used to detect the changes of cardiac electrophysiology. The data was expressed as means ± SD. N=8. ***P<0.01*. Normality was confirmed using the Shapiro-Wilk test. Two-group comparisons were analyzed by Student’s t-test. BP, blood perfusion. EMR, erythrocyte migration rate. IS, ischmia. RP, reperfusion.

**Figure S2** **Changes in RNA methylation and ferroptosis levels in the HH/R cell model and DIR animal model**

As shown in Fig. S2A, we extracted NMVCs from primary rat cardiomyocytes and detected the expression of the MHC protein in NMVCs via immunofluorescence. The MHC protein was expressed in all the extracted cells.

Changes in RNA methylation and ferroptosis levels were validated in the HH/R H9c2 cell model, the NMVC primary cell model, and the animal model. As shown by the dot blot results in Fig. S2B, detection of the 200 ng and 400 ng mRNAs revealed that the overall level of m6A was significantly increased in the H9c2 cell model, the primary NMVC cell model, and the animal model. We then examined the expression levels of the RNA demethylases ALKBH5 and FTO in the three models.

As shown in Fig. S2C, compared with that in the normal group, the protein level of ALKBH5 was significantly greater in the HH/R and DIR groups in the three models (*P<0.01*). In both the H9c2 and NMVC cell models, FTO protein levels were significantly greater in the HH/R group than in the normal group (*P*<0.05), and there was no significant difference in the protein level of ALKBH5. However, the protein level of ALKBH5 in the DIR group was not significantly different from that in the normal group. These findings suggest that the RNA demethylase ALKBH5 protein changes more obviously than does the FTO protein during myocardial ischemia‒reperfusion injury. The protein and mRNA levels of ALKBH5, cell damage indicators, cell viability, LDH, the ferroptosis-related molecule Fe^2+^, and GPX4 were detected in the three models. As shown in Fig. S2D–F, compared with those in the normal group, cell viability and the mRNA and protein levels of ALKBH5 and GPX4 were significantly lower in the HH/R model H9c2 cells (*P*<0.01), whereas the Fe^2+^ and LDH levels were significantly greater (*P*<0.01). The same trend was observed for the above indicators in the HH/R model of NMVC cells.

As shown in Fig. S2G, the cardiomyocytes in the myocardial tissue of the normal group had a normal morphology, a regular and neat arrangement, and small intercellular gaps. In the myocardial tissue of the DIR group, the arrangement of the myocardial cells was disordered, the myocardial fibers were broken, and the intercellular space became larger. Fig. S2G also shows the electron microscopy of the mitochondrial structure in the myocardial tissue of the normal group, which was intact, and the continuity of mitochondrial cristae was not damaged. In the myocardial tissue structure of the DIR group, the mitochondrial structure was destroyed, and the continuity of the mitochondrial cristae was disrupted. The TTC results in Fig. S2G revealed that there were no infarcted foci in the myocardial tissue of the normal group, whereas the infarct area of the myocardial tissue of the rats in the DIR group was significantly enlarged. As shown in Fig. S2H, compared with those in the normal group, the mRNA and protein levels of ALKBH5 and GPX4 in the serum and myocardial tissue of the DIR group were significantly lower (*P<0.01*), whereas the levels of CK-MB, LDH, cTnl, and Fe^2+^ were significantly greater (*P<0.01*). Therefore, the m6A RNA methylation level and the expression of the RNA demethylase ALKBH5 were significantly increased during myocardial ischemia‒reperfusion injury in diabetic rats. Moreover, the degree of myocardial injury and ferroptosis were also significantly increased.

**
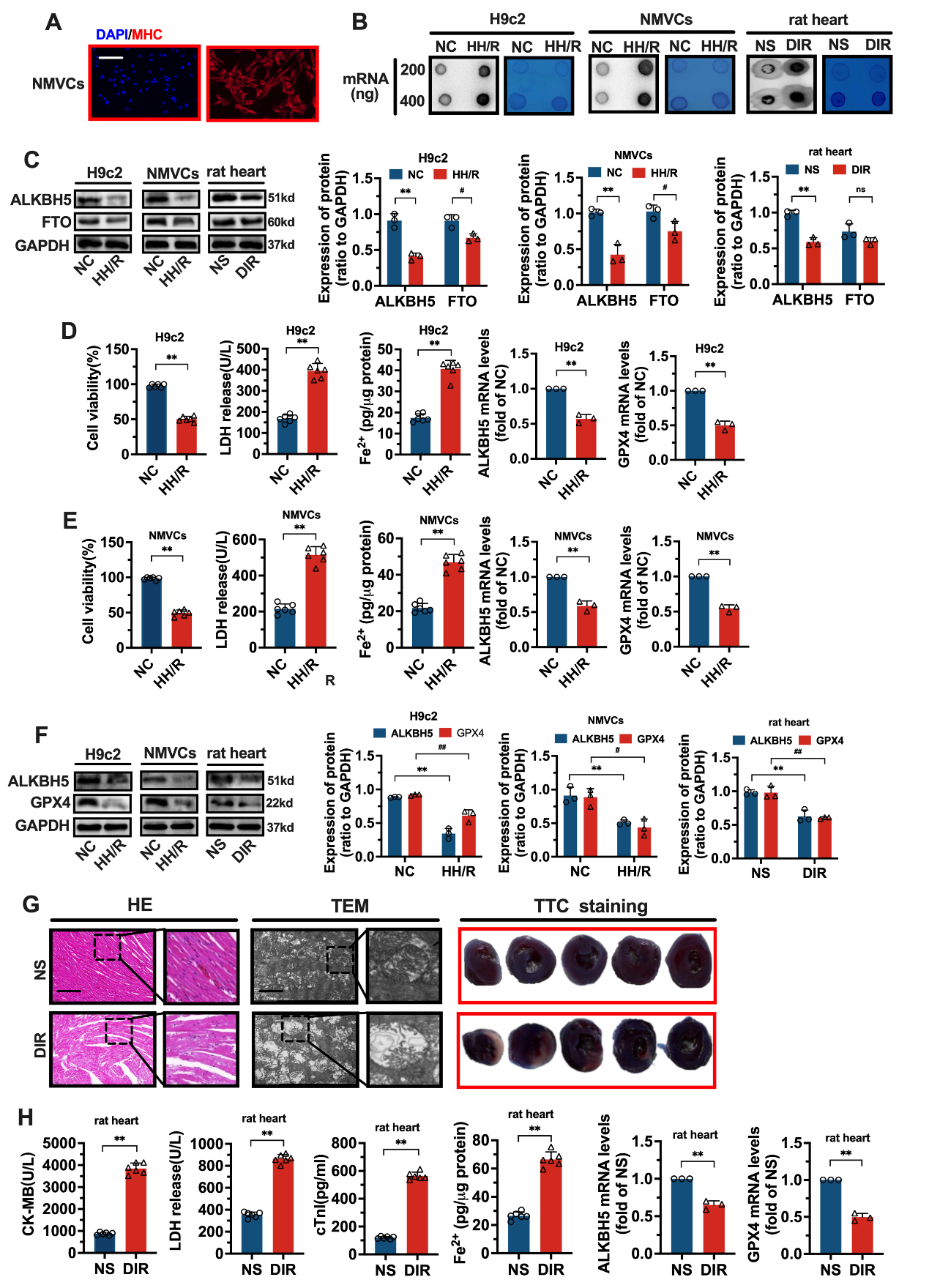
**

**Figure S2 legend** Changes in RNA methylation, ferroptosis levels in HH/R cell model and DIR animal model. (A) Immunofluorescence detection of MHC protein in NMVCs of primary rat cardiomyocytes. (B) Dot blot was used to detect the overall levels of m6A in H9c2 and NMVCs cell models and animal models at 200ng and 400ng mRNA levels. (C) Western blot was used to detect the changes of RNA demethylases ALKBH5 and FTO in H9c2 and NMVCs cell models and animal models. (D) The cell viability, LDH and Fe^2+^ levels were detected by kit, the mRNA levels of ALKBH5 and GPX4 were detected by RT-PCR, and the protein levels of ALKBH5 and GPX4 were detected by western blot in H9c2 cell models. (E) The cell viability, LDH and Fe^2+^ levels were detected by kit, the mRNA levels of ALKBH5 and GPX4 were detected by RT-PCR, and the protein levels of ALKBH5 and GPX4 were detected by western blot in NMVCs cell models. (F) Western blot was used to detect the changes of ALKBH5 and GPX4 in H9c2 and NMVCs cell models and animal models. (G) HE staining (left), TEM (middle), and TTC staining (right) were used to detect the pathological changes, ultrastructural pathological changes and infarct size of myocardial tissue. (H) The levels of CK-MB, LDH, cTnl and Fe^2+^ were detected by test kit. The mRNA levels of ALKBH5 and GPX4 were detected by RT-PCR. The data was expressed as means ± SD. N=3 or 6. Normality was confirmed using the Shapiro-Wilk test. Two-group comparisons were analyzed by Student’s t-test. ***P<0.01*. ##*P<0.01*. #*P<0.05*. ns, no significant. The scale bars in Figure A represent 100μm.

**Figure S3** **ALKBH5 and GPX4 expression at different timepoints in** **DIR model**

Extending reperfusion to days (e.g., day 0, 1, 7, 14) was impractical due to the technical Challenges, open-chest DIR models require ventilator support, increasing infection risk and mortality with prolonged anesthesia. For the pathological Relevance: Chronic reperfusion (days) reflects remodeling rather than acute injury, which is the primary focus of our ferroptosis mechanistic study. Existing data show that acute injury markers (e.g., MDA, TNF-α) stabilize by 24h [1], consistent with our finding of no significant changes beyond 2h.

In order to clarify the rationale for our timepoint selection in the DIR model, we chose a 30-minute ischemia period based on established literature, as irreversible myocardial injury typically initiates after 20–40 minutes of ischemia [2, 3]. This duration ensures reproducible infarction while minimizing excessive tissue damage that could confound results.

Our timepoints (1h, 2h, 6h, 12h, 24h) focus on the acute phase of reperfusion injury, during which oxidative stress, inflammation, and ferroptosis are most pronounced. Recent studies emphasize that the earliest reperfusion hours (0–2h) drive peak damage due to neutrophil infiltration, mitochondrial dysfunction, and metabolic reprogramming [4]. Specifically, neutrophil N1 polarization and lactate-mediated mitochondrial damage peak within 2 hours [4], aligning with our observation of maximal injury at 2h.

We set reperfusion timepoints at 1h, 2h, 6h, 12h, and 24h and found that myocardial injury was most severe at 2h post-reperfusion. As shown in Figure S3, no significant differences in injury severity were observed between 6h and 24h compared to 2h. This aligns with literature demonstrating that acute reperfusion injury peaks within 2-6 hours due to oxidative stress and neutrophil infiltration[5], while prolonged reperfusion beyond 24 hours shifts the pathological focus to chronic remodeling rather than acute ferroptosis [6].

**
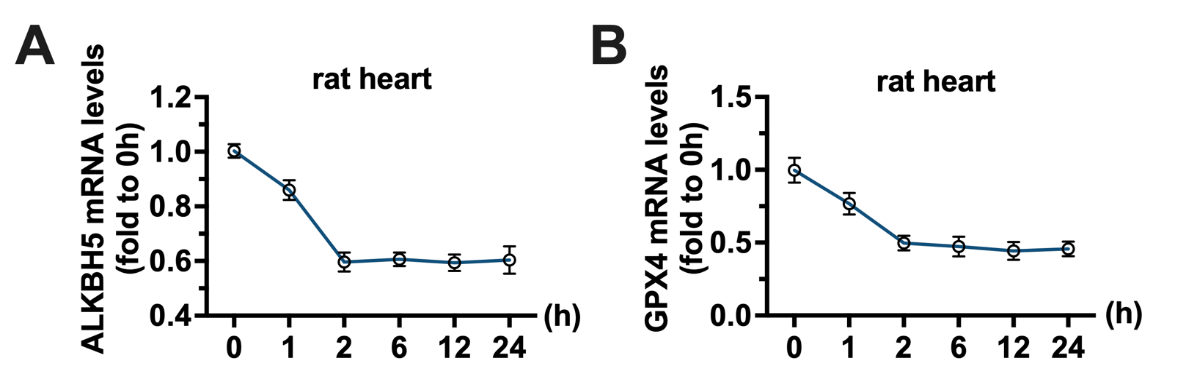
**

**Figure S3** **legend** (A) ALKBH5 and (B) GPX4 mRNA expression at different timepoints (0h, 1h, 2h, 6h, 12h, 24h) in the acute phase of reperfusion injury for DIR rat model. Normality was assessed using the Shapiro-Wilk test. For multiple groups, one-way ANOVA with Tukey’s post-hoc test was applied. N=3. The data was expressed as means ± SD.

**Figure S4 Validation of Adeno-associated Virus (AAV) Interventions for ALKBH5**

As shown in Fig. S4A–B, we first verified the effect on ALKBH5 expression after adeno-associated virus transfection; ALKBH5 mRNA and protein levels were not significantly different between the AVV-Ctrl group and the NS group. AVV-ALKBH5 treatment significantly increased the mRNA and protein levels of ALKBH5 (*P<0.01 or 0.05*). ALKBH5 could be overexpressed successfully by an adeno-associated virus vector, which could be used in subsequent experiments.

**
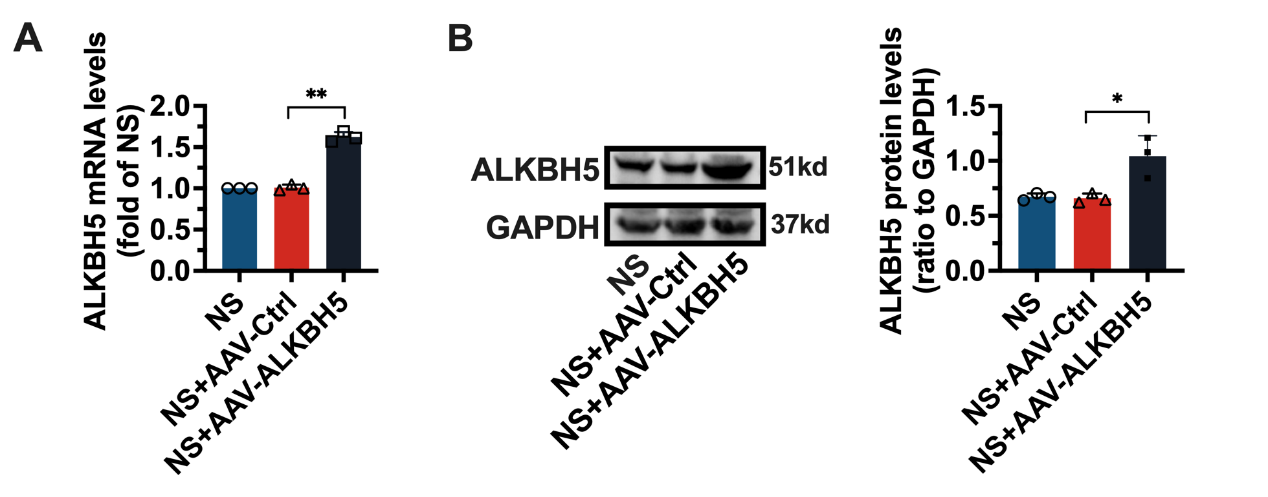
**

**Figure S4** **legend** Verify the effect of adeno-associated virus (AAV) - mediated overexpression of ALKBH5 in rat heart. (A) RT-PCR was used to detect the expression levels of ALKBH5 mRNA in myocardial tissues of each group. (B) Western blot was employed to detect the expression levels of ALKBH5 protein in myocardial tissues of each group. The data was expressed as means ± SD. N=3. Normality was confirmed using the Shapiro-Wilk test. Two-group comparisons were analyzed by Student’s t-test. ***P<0.01*, **P<0.05*.

**Figure S5 Effects of ALKBH5 on ferroptosis related markers in the DIR model**

As shown in Fig. S5A-D, compared with those in the NS group, the levels of ROS, lipid peroxidation markers (MDA, 4-HNE), and ACSL4 mRNA level in the serum and myocardial tissue of the DIR group were significantly greater (*P<0.01*). Compared with those in the DIR group, the levels of ROS, MDA, 4-HNE, and ACSL4 mRNA level in the serum and myocardial tissue of the AAV-ALKBH5 group were significantly lower (*P<0.01*).

**
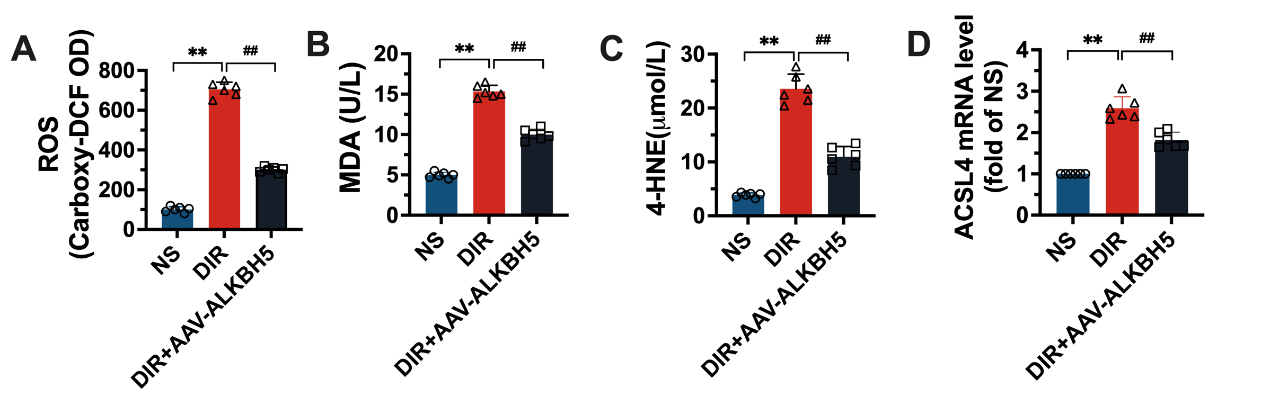
**

**Figure S5** The effect of ALKBH5 on ferroptosis-related markers in DIR rats. (A-C) The levels of ROS, MDA, 4-HNE were detected by test kit. (D) The mRNA level of ACSL4 was detected by RT-PCR. The data was expressed as means ± SD. N=6. Normality was confirmed using the Shapiro-Wilk test. Two-group comparisons were analyzed by Student’s t-test. ***P<0.01*, ##*P<0.01*.

**Figure S6 Validation of** **Lentivirus (LV) Interventions for ALKBH5 in H9c2 and NMVCs cells**

As shown in Fig. S6A-B, we first verified the effect of lentivirus transfection on ALKBH5 expression in H9c2 and NMVCs cells, and ALKBH5 mRNA and protein levels were not significantly different between the LV-Ctrl group and the NC group. After LV-ALKBH5 treatment, the ALKBH5 mRNA and protein levels were significantly increased (*P<0.01 or 0.05*). The constructed lentivirus can successfully overexpress the ALKBH5 molecule, which can be used for subsequent experiments.


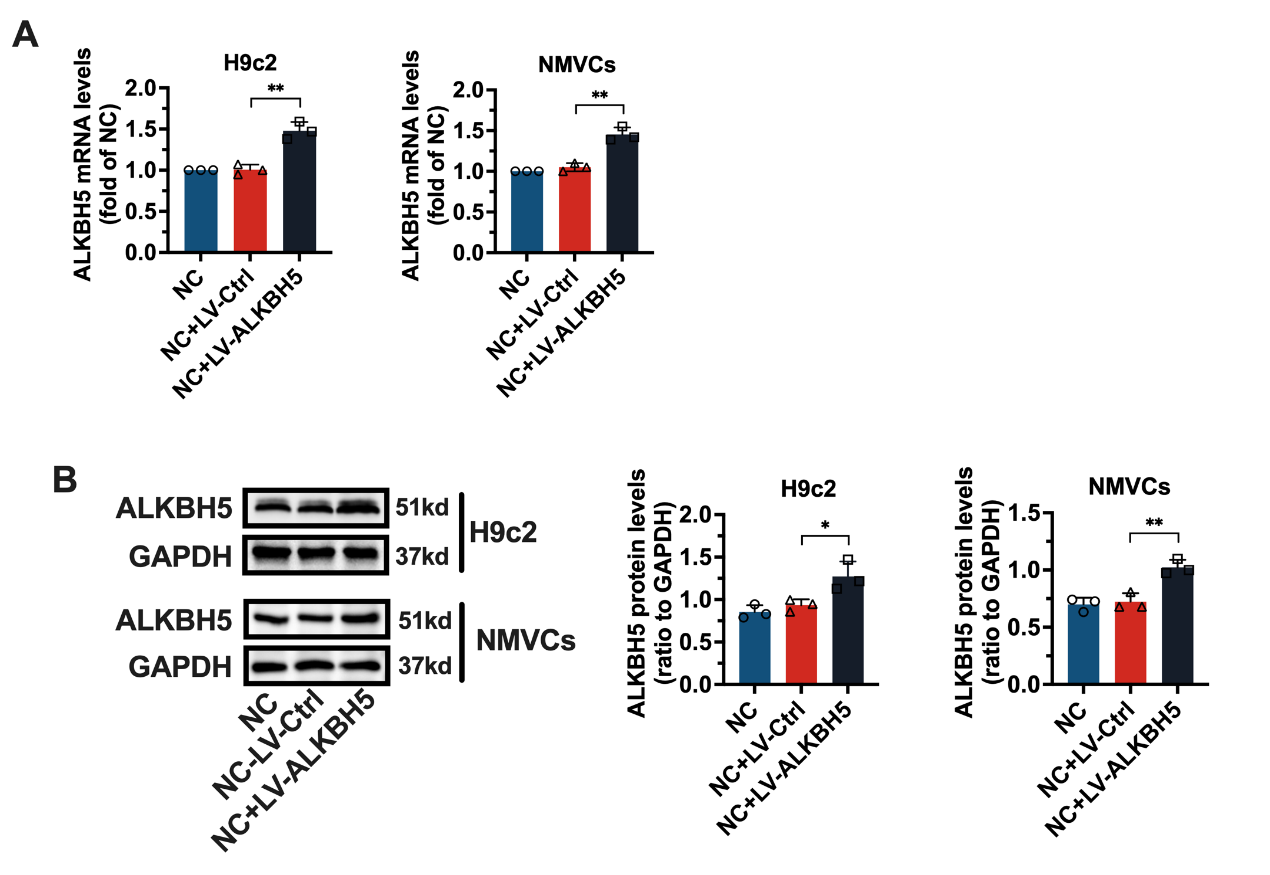


**Figure S6** Verify the effect of Lentivirus (LV) - mediated overexpression of ALKBH5 in cells. (A) The mRNA level of ALKBH5 in H9c2 and NMVCs cells was detected by RT-PCR. (B) The protein level of ALKBH5 protein in H9c2 and NMVCs cells was detected by western blot. The data was expressed as means ± SD. N=3. Normality was confirmed using the Shapiro-Wilk test. Two-group comparisons were analyzed by Student’s t-test. ***P<0.01*, **P<0.05*.

**Figure S7 Effects of ALKBH5 on ferroptosis related** **markers in the HH/R model**

As shown in Fig. S7A-C, compared with those in the NC group, the levels ROS, MDA, and 4-HNE, and ACSL4 mRNA level in the HH/R group of H9c2 cells were significantly greater (*P*<0.01). Compared with those in the HH/R group, the ROS, MDA, 4-HNE and ACSL4 mRNA levels in the LV-ALKBH5 group were significantly lower (*P<0.01 or 0.05*). Compared with those in the HH/R group, the levels of ROS, MDA, and 4-HNE, and ACSL4 mRNA level in the erastin group were further increased (*P<0.01 or 0.05*). Compared with those in the HH/R+Era and HH/R+LV-ALKBH5 groups, the levels of ROS, MDA, and 4-HNE, and ACSL4 mRNA level in the HH/R+Era+LV-ALKBH5 group were decreased and increased (*P<0.01 or 0.05*). Similarly, the changes of above markers in NMVC cells were consistent with those in H9c2 cells.

**
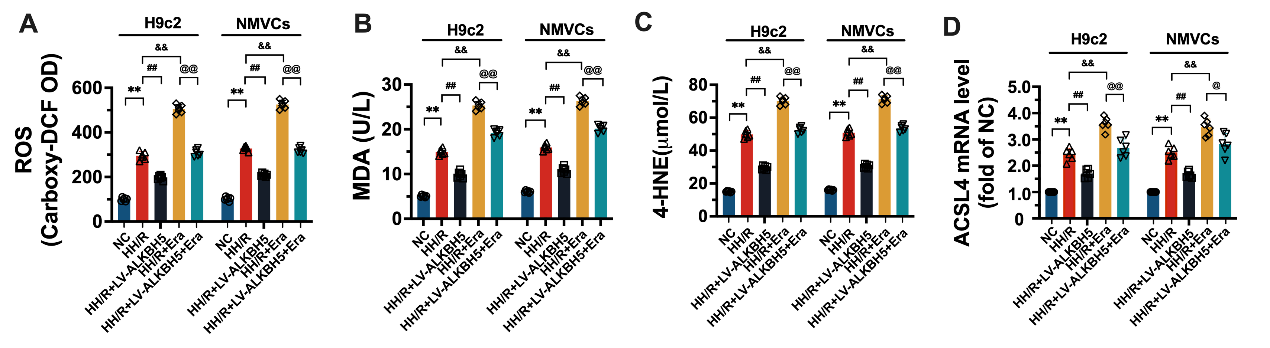
**

**Figure S7** The effect of ALKBH5 on ferroptosis-related markers in HH/R cell models. (A-C) The levels of ROS, MDA, 4-HNE in H9c2 and NMVCs cells were detected by test kits. (D) The mRNA level of ACSL4 in H9c2 and NMVCs cells was detected by RT-PCR. The data was expressed as means ± SD. N=6. Normality was assessed using the Shapiro-Wilk test. For comparisons between two groups, Student’s t-test was used. For multiple groups, one-way ANOVA with Tukey’s post-hoc test was applied. ***P<0.01*, *P< 0.05. ##*P< 0.01*, #*P<0.05*. @@*P<0.01*, @P< 0.05. &&*P< 0.01*, &*P<0.05*.

**Figure S8 Verify the effect of LV-mediated overexpression of METTL3 and FTO, and the effect of METTL3 and FTO on ferroptosis-related markers in HH/R cell models**

As shown in Fig. S8A-B, we first verified the effect of lentivirus transfection on METTL3 and FTO expression in H9c2 cells, and METTL3 and FTO mRNA levels were not significantly different between the LV-Ctrl group and the NC group. After LV- METTL3 and FTO treatment, the METTL3 and FTO mRNA levels were significantly increased (*P<0.01 or 0.05*). The constructed lentivirus can successfully overexpress the METTL3 and FTO molecule, which can be used for subsequent experiments.

As shown in Supplementary Fig. S8C–F, ALKBH5 and FTO overexpression in HH/R cells significantly reduces Fe^2+^ level, mitochondrial ROS (P<0.05 or P<0.01) and lipid peroxidation markers (MDA, 4-HNE; P<0.05 or P<0.01), while METTL3 overexpression in HH/R cells significantly elevated Fe^2+^, ROS, MDA and 4-HNE levels (P<0.01). When compared with HH/R+LV-FTO group, the levels of Fe^2+^, ROS, MDA and 4-HNE in HH/R+LV-ALKBH5 group is lower (P<0.05). LV-mediated ALKBH5 overexpression in HH/R+METTL3 group paradoxically alleviated Fe²⁺, ROS, MDA and 4-HNE levels (P<0.05 or P<0.01), suggesting METTL3 may antagonize ALKBH5 in this context.

**
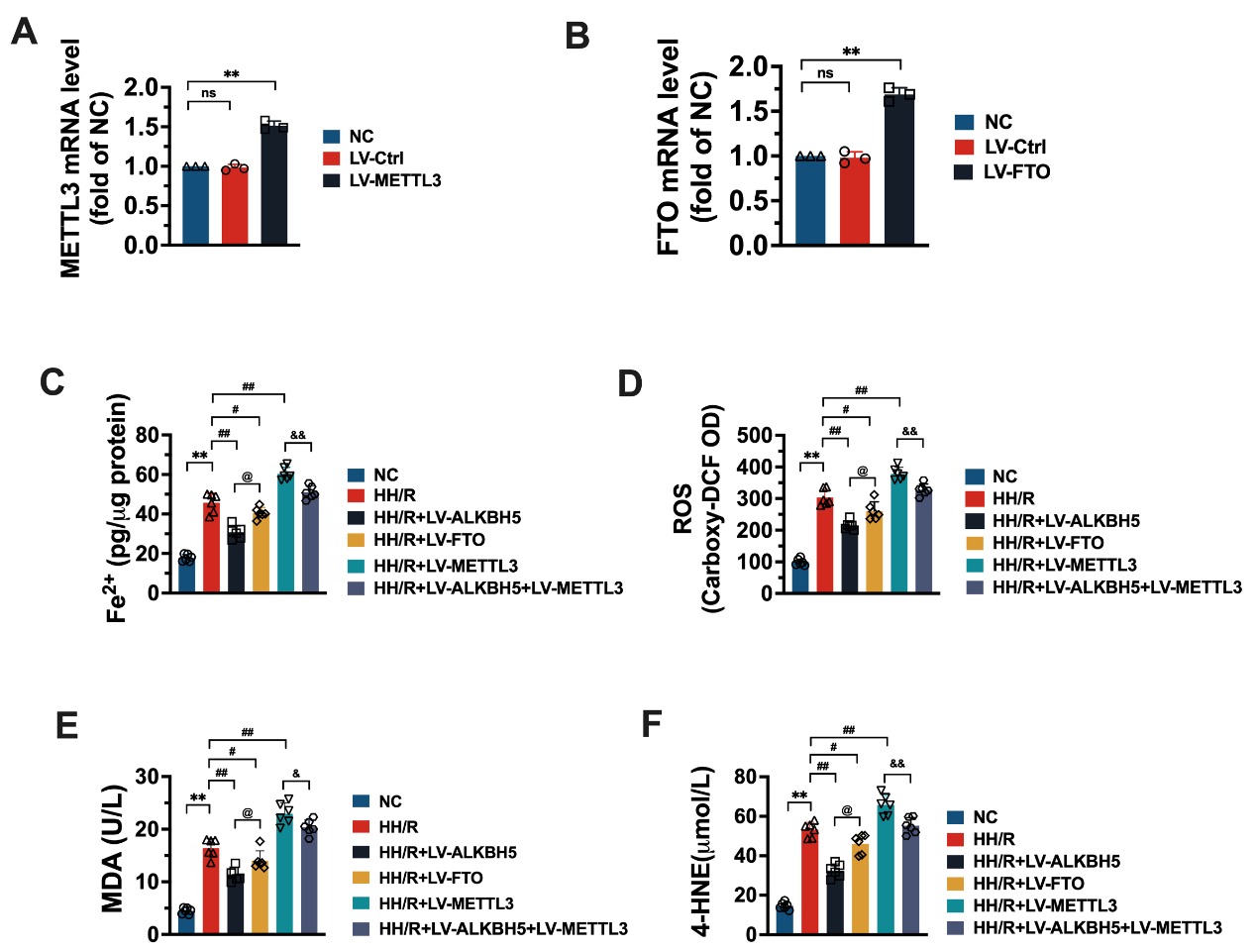
**

**Figure S8 legend** Verify the effect of LV-mediated overexpression of METTL3 and FTO, and the effect of METTL3 and FTO on ferroptosis-related markers in HH/R cell models. (A-) The mRNA level of METTL3 and FTO in H9c2 cells was detected by RT-PCR. (C-F) The levels of Fe^2+^, ROS, MDA, 4-HNE were detected by test kit. The data was expressed as means ± SD. N=6. Normality was assessed using the Shapiro-Wilk test. For comparisons between two groups, Student’s t-test was used. For multiple groups, one-way ANOVA with Tukey’s post-hoc test was applied. ***P<0.01*, *P< 0.05. ##*P< 0.01*, #*P<0.05*. @@*P<0.01*, @P< 0.05. &&*P< 0.01*, &*P<0.05*.

**Figure S9 Effect of G3BP1 3’UTR region and m6A post on ferroptosis of cardiomyocytes**

As shown in Fig. S9A, after overexpression of ALKBH5, the degradation of G3BP1 mRNA was slower in NMVC cells than in control cells. As shown in Fig. S9B, ALKBH5 knockdown significantly reduced the fluorescence intensity in H9c2 cells (*P<0.01*). In the mutant 3'-UTR reporter plasmid group, ALKBH5 knockdown did not significantly change the fluorescence intensity. Moreover, when ALKBH5 was knocked down, the fluorescence intensity in the mutant 3'-UTR reporter plasmid group was significantly greater than that in the wild-type 3'-UTR reporter plasmid group.

As shown in Fig. S9C, a plasmid (3'-UTR Mut) containing the 3'-UTR region of the mutant G3BP1 gene was constructed, and a wild-type plasmid (3'-UTR WT) was synthesized. H9c2 and NMVC cells were transfected with an ALKBH5-overexpressing lentivirus (LV-ALKBH5) or a control lentivirus (Ctrl), and the degradation rate of G3BP1 mRNA was detected and analyzed. In both H9c2 and NMVC cells, mRNA degradation was significantly reduced after ALKBH5 overexpression in the wild-type 3'-UTR plasmid group. There was no significant difference in the mRNA degradation rate among the 3'-UTR mutant plasmids. In addition, there was no significant difference in the mRNA degradation rate between the mutant 3'-UTR plasmid group and the wild-type 3'-UTR plasmid group under the same conditions in which ALKBH5 was overexpressed. ALKBH5 expression affects the stability of the G3BP1 3'-UTR, which is dependent on the demethylase activity of ALKBH5 and the location of the G3BP1 mRNA 3'-UTR.

As shown in Fig. S9D, NMVC cells were treated with sirNA-ALKBH5 or control siRNA (Ctrl) and transfected with reporter plasmids (WT, Mut 142 or Mut 173). The luciferase assay was subsequently performed according to the instructions of the dual luciferase kit, and the m6A site of the 3′-UTR of G3BP1 was determined by the change in the fluorescence value. As shown in Fig. S9E, ALKBH5 knockdown significantly reduced the fluorescence intensity in NMVC cells (*P<0.01*). In the Mut 142 and Mut 173 reporter plasmid groups, the fluorescence intensity of the Mut 142 and Mut 173 reporter plasmid groups was significantly greater than that of the wild-type 3'-UTR reporter plasmid group after ALKBH5 knockdown.

**
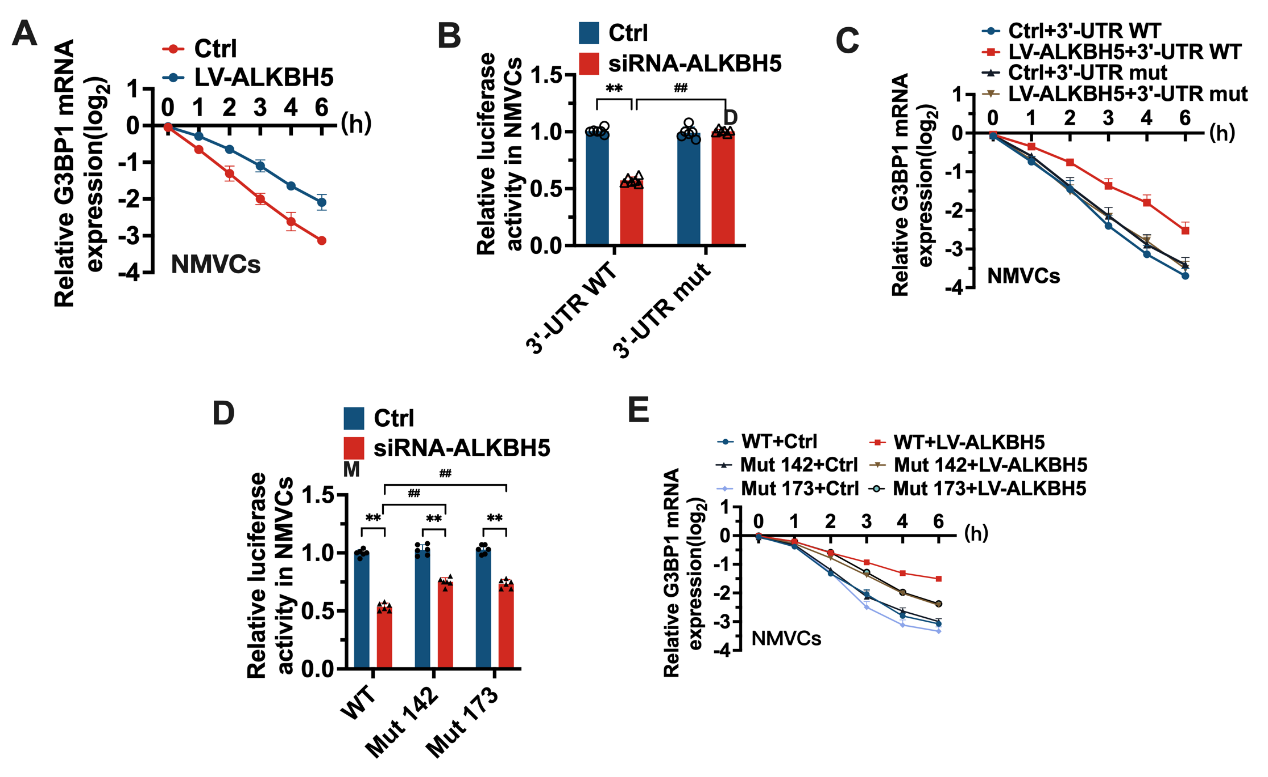
**

**Figure S9 legend** Effect of G3BP1 3’UTR region and m6A post on ferroptosis of cardiomyocytes. (A, C, E) Determination of the rate of G3BP1 mRNA degradation in NMVCs cells. (B, D) Dual-luciferase assay was used to detect the fluorescence intensity of the reporter gene in NMVCs cells. The data was expressed as means ± SD. N=3 or 6. Normality was assessed using the Shapiro-Wilk test. For comparisons between two groups, Student’s t-test was used. For multiple groups, one-way ANOVA with Tukey’s post-hoc test was applied. ***P<0.01*, ##*P<0.01*.

**Figure S10 Validation of siRNA** **Interventions for G3BP1 and YBX1, LV Intervention for** **G3BP1**

As shown in Fig. S10A, we verified the effect of siRNA transfection on G3BP1 expression in H9c2 cells, and G3BP1 protein levels did not change significantly in the siCtrl group compared with those in the NC group. However, after siRNA-mediated G3BP1 intervention, the G3BP1 protein level was significantly decreased (*P<0.01*). This finding indicated that the siRNA itself did not affect the expression of the target molecule in H9c2 cells and that the constructed siRNA could successfully knock down the G3BP1 molecule, which could be used for subsequent experiments.

As shown in Fig. S10B–C, we verified the effects of LV and siRNA transfection on the expression of G3BP1 and YBX1 in H9c2 cells, and G3BP1 protein and mRNA levels were not significantly different between the LV-Ctrl group and the NC group. However, after LV-G3BP1 intervention, the protein and mRNA levels of G3BP1 were significantly decreased (*P<0.01*). Compared with those in the NC group, the protein and mRNA levels of YBX1 in the siCtrl group did not change significantly. However, after siYBX1 intervention, the protein and mRNA expression levels of YBX1 were significantly decreased (*P<0.01*). These findings indicated that LV or siRNA alone could not affect the expression of G3BP1 or YBX1 in H9c2 cells and that the constructed LV or siRNA could successfully overexpress G3BP1 and knock down the YBX1 molecule, which could be used for subsequent experiments.

**
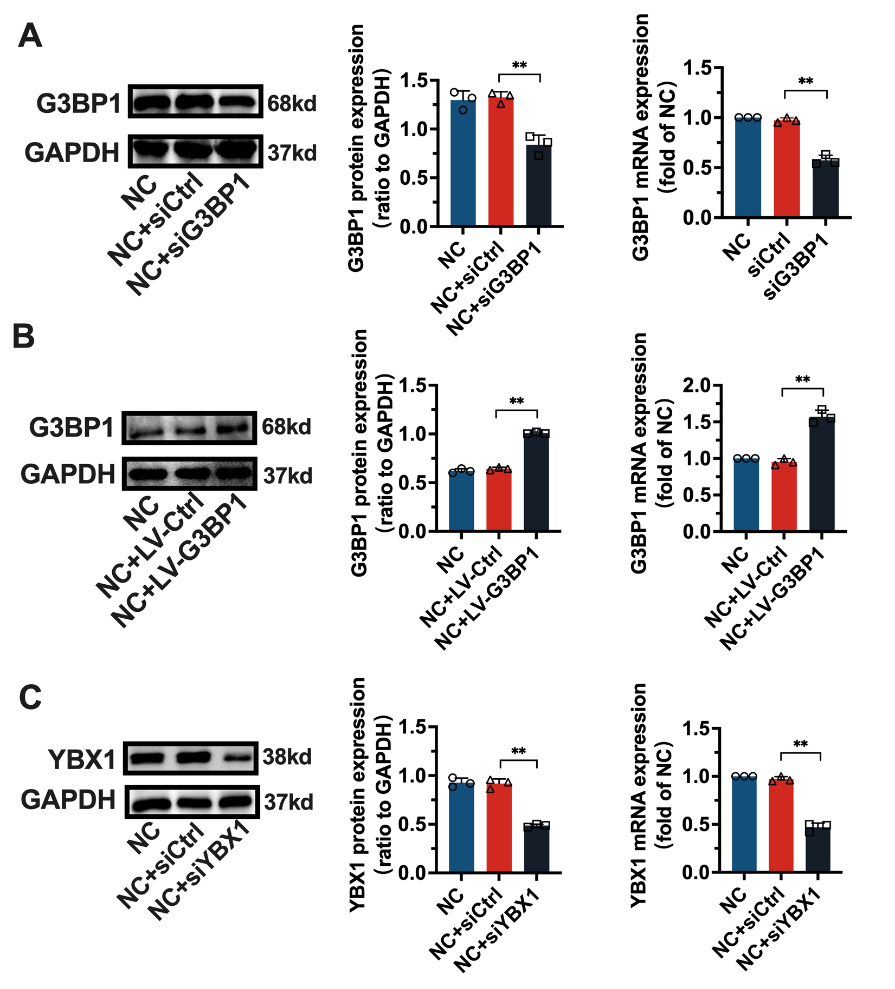
**

**Figure S10 legend** Verify the effect of siRNA-mediated down-expression of G3BP1 and YBX1, LV-mediated overexpression of G3BP1 in cells. (A-B) The protein and mRNA levels of ALKBH5 in H9c2 cells were detected by western blot and RT-PCR. (C) The protein and mRNA levels of YBX1in H9c2 cells were detected by western blot and RT-PCR. The data was expressed as means ± SD. N=3. Normality was confirmed using the Shapiro-Wilk test. Two-group comparisons were analyzed by Student’s t-test. ***P<0.01*. T test was used.

**Figure S11 legend** **Changes in the distribution and expression of p53 and YBX1 proteins after different interventions in the HH/R cell model and the DIR rat model.**

As shown in Fig. S11A–H, YBX1 and p53 were found to be colocalized in the cytoplasm in H9c2 cells and normal cardiac tissue under normoxic conditions. Under conditions of HH/R in cells and DIR in tissues, the combination was enhanced and transferred to the nucleus (*P<0.01*).

As shown in Fig. S11 I–L, knockdown or overexpression of YBX1 had no effect on the expression or distribution of p53 in H9c2 cells under normoxic conditions. When the expression of YBX1 was knocked down, the expression of p53 in the nucleus decreased (*P<0.01*). The expression of p53 in the HH/R group was not significantly greater than that in the HH/R group. As shown in Fig. 9D and Fig. S8M–P, when knockdown or overexpression of YBX1 in the myocardial tissue, the p53 and YBX1 showed same trend as that observed in the cell experiments

As shown in Fig. S11Q, p53 knockdown under H/R conditions reduced YBX1 nuclear translocation (*P<0.01*). As shown in Fig. S11R-S, importin-β1 is a classical protein that promotes p53 nuclear translocation. Importin-β1 knockdown also reduced the level of YBX1 nuclear translocation (*P<0.01*), indicating that p53 is required for YBX1 nuclear translocation.

**
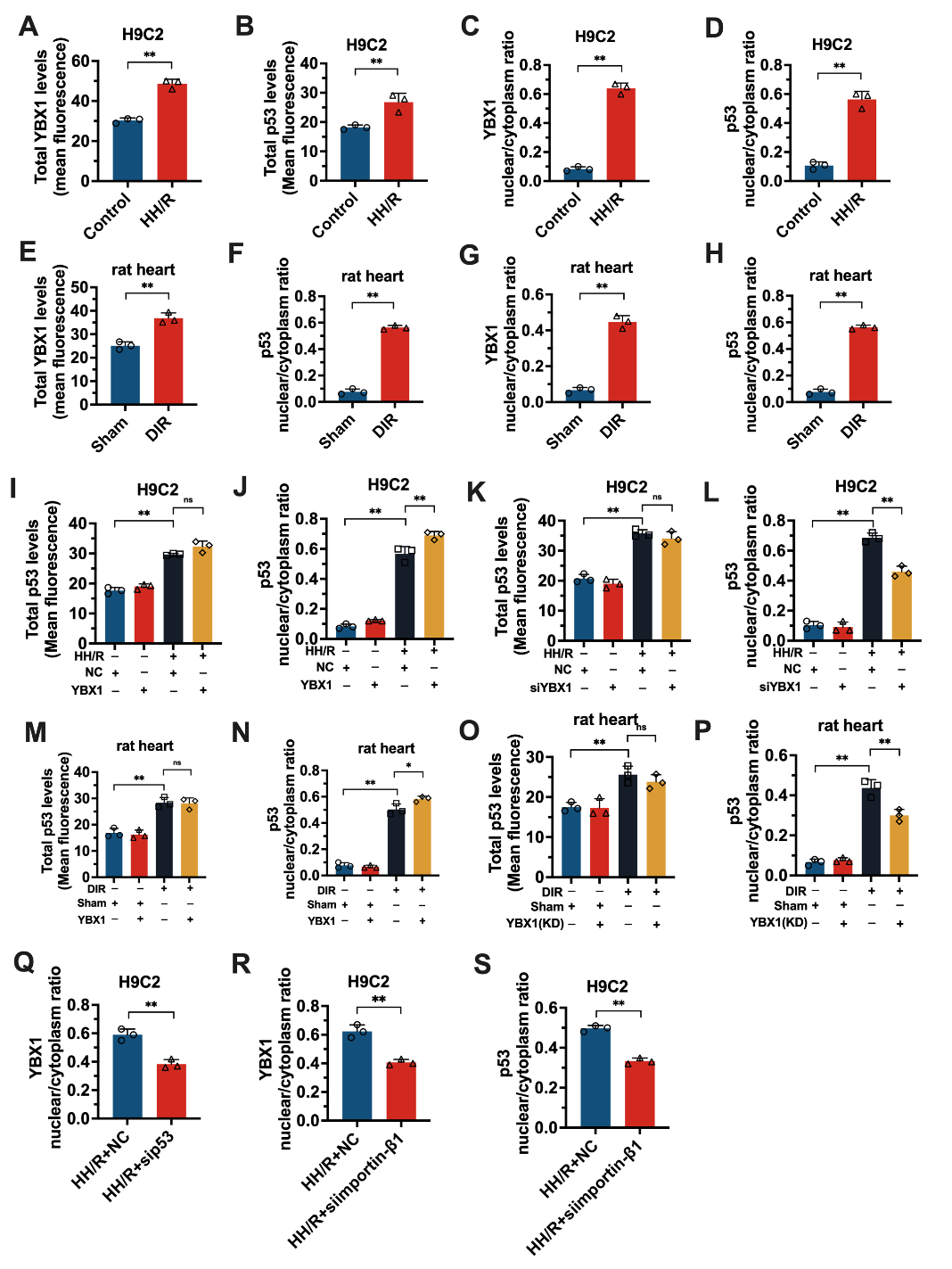
**

**Figure S11** Changes in the distribution and expression of p53 and YBX1 proteins after different interventions in the HH/R cell model and the DIR rat model. (A-D) In H9C2 cells, bar graphs showing the total YBX1 levels (A), total p53 levels (B), nuclear - cytoplasmic ratio of YBX1 (C), and nuclear-cytoplasmic ratio of p53 (D) under control and HH/R conditions. (E-H) In rat hearts, bar graphs presenting the total YBX1 levels (E), total p53 levels (F), nuclear - cytoplasmic ratio of YBX1 (G), and nuclear-cytoplasmic ratio of p53 (H) under Sham and DIR conditions, with statistical significance shown. (I-L) In H9C2 cells, bar graphs displaying the total p53 levels (I, K) and nuclear-cytoplasmic ratio of p53 (J, L) under H/R conditions with different treatments including NC, YBX1 overexpression, and siYBX1. (M-P) In rat hearts, bar graphs showing the total p53 levels (M, O) and nuclear-cytoplasmic ratio of p53 (N, P) under DIR conditions with various treatments such as YBX1 overexpression and YBX1(KD) (kinase-dead YBX1). Statistical significance is marked. (Q-S) In H9C2 cells, bar graphs presenting the nuclear - cytoplasmic ratio of YBX1 (Q, R) and p53 (S) under H/R conditions with treatments like siP53 (p53 knockdown) and importin - β1 overexpression. The data was expressed as means ± SD. N=3. Normality was confirmed using the Shapiro-Wilk test. Two-group comparisons were analyzed by Student’s t-test. ***P<0.01*. **P<0.05*. ns, no significant.

**Figure S12** **The effects of intervening YBX1 and p53 on apoptosis and membrane potential in the HH/R cell model.**

As shown in Fig. S12A–D, YBX1 overexpression increased the HH/R-induced apoptosis rate (*P<0.01*), and the HH/R-induced apoptosis rate decreased after YBX1 was knocked down (*P<0.01*). There was potentially dependent accumulation of JC-1 dye within the mitochondria. When the mitochondrial membrane potential was high, JC-1 aggregated in the mitochondrial matrix to form aggregates, which emitted intense red fluorescence. When the mitochondrial membrane potential decreased, JC-1 could not aggregate and was in the monomer form, emitting green fluorescence. When cell damage occurs, the mitochondrial membrane potential decreases, and the aggregation/monomer ratio decreases. YBX1 overexpression further reduced the HH/R-induced aggregation/monomer ratio (*P<0.01*), whereas the ratio was increased by the knockdown of YBX1 (*P<0.01*). As shown in Fig. S12E, TUNEL staining revealed that p53 knockdown reversed Ybx1-induced apoptosis (*P<0.01*).

**
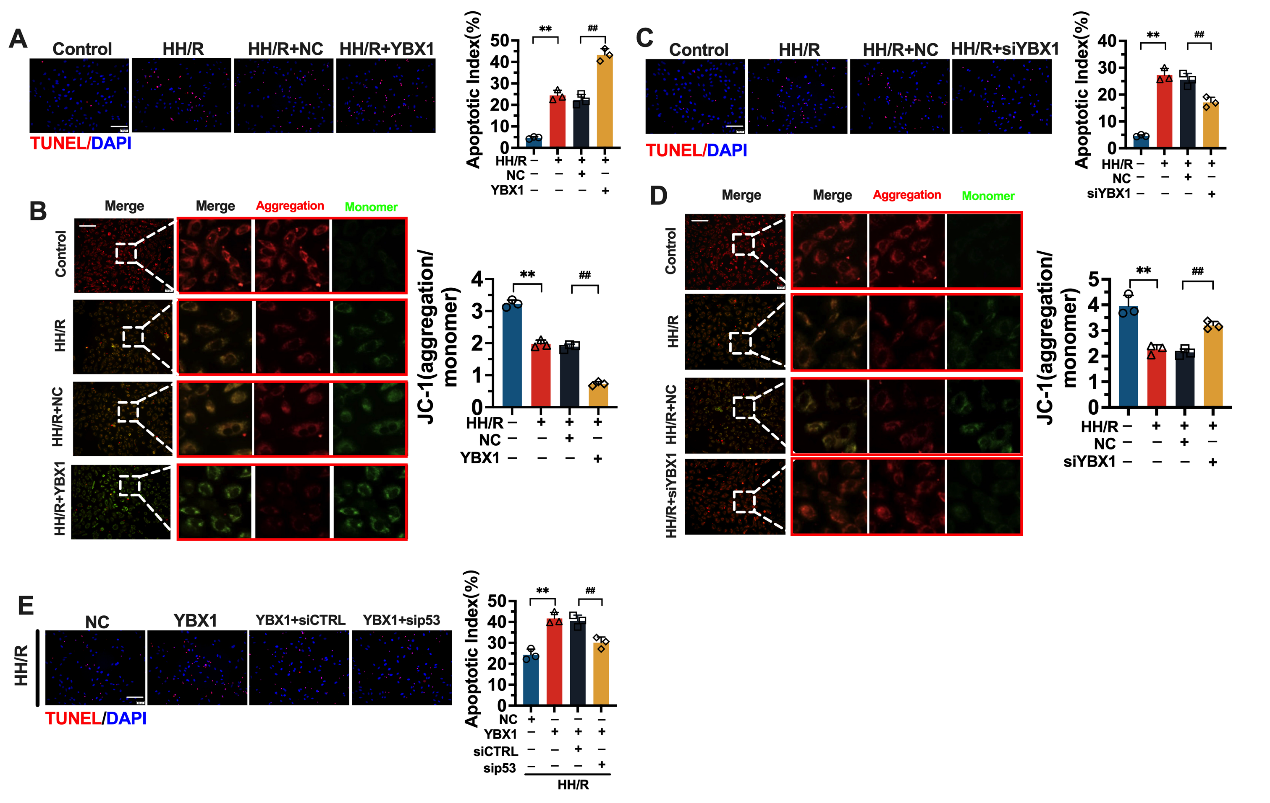
**

**Figure S12 legend** The effects of intervening YBX1 and p53 on apoptosis and membrane potential in the HH/R cell model. (A, C, E) TUNEL staining was used to detect the apoptosis rate of myocardial tissue. (B, D) Mitochondrial membrane potential assay kit with JC-1 was used to detect mitochondrial membrane potential. The data was expressed as means ± SD. N=3. Normality was confirmed using the Shapiro-Wilk test. Two-group comparisons were analyzed by Student’s t-test. The scale bars in Figure A, B, C, D, and E represent 20μm.***P<0.01*. ##*P<0.01*.

**Figure S13 The Statistical Graph for** **Fig. 2J, Fig. 6C, Fig. 6F, and Fig. 8G**

As shown in Fig. S13A-B, compared with NS rats, the expression levels of GPX4 and G3BP1 proteins in myocardial tissue of DIR rats were significantly decreased (*P<0.01*). Compared with the DIR group, the expressions of GPPX4 and G3BP1 proteins were significantly increased in the DIR+AAV-ALKBH5 group (*P<0.01 or 0.05*). As shown in Fig. S13C, compared with that in the NC group, the protein level of G3BP1 was significantly lower in the HH/R group (*P<0.01*). Compared with those in the HH/R group, the G3BP1 protein level in the LV-ALKBH5 group was significantly greater (*P<0.01*), the G3BP1 protein level was lower in the siRNA-G3BP1 group (*P<0.01*), and the protein level of YBX1 was further increased. Compared with the siRNA-G3BP1 group, the LV-ALKBH5+ siRNA-G3BP1 group presented increased G3BP1 protein levels (*P<0.01*).

**
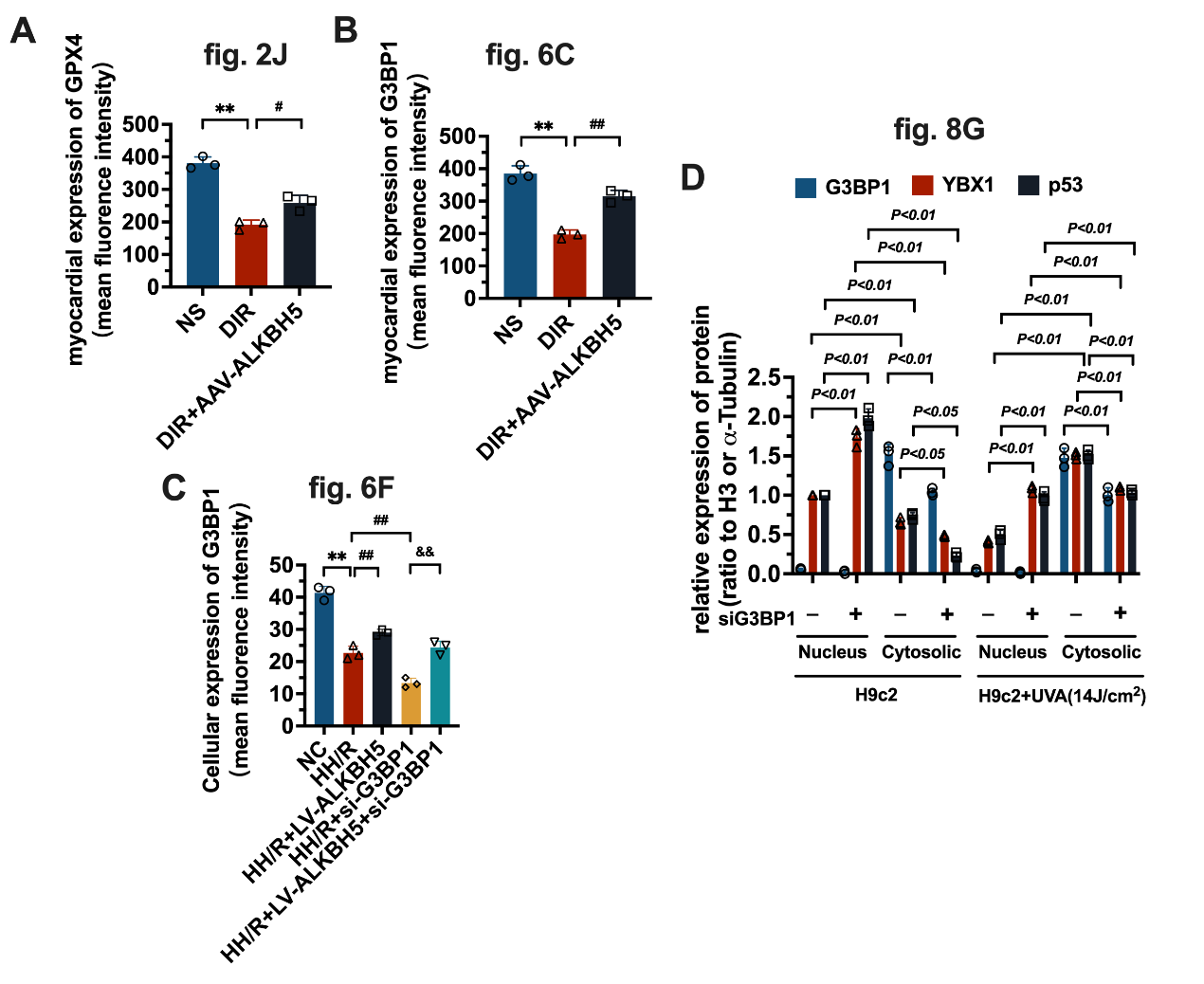
**

**Figure S13 legend** The Statistical Graph for Fig. 2J, Fig. 6C, Fig. 6F, and Fig. 8G. (A-B) Quantitative plot of GPX4 and G3BP1 expression in myocardial tissue detected by immunofluorescence. (C) Quantitative plot of G3BP1 expression in H9c2 cells detected by immunofluorescence. (D) Quantitative plots of protein expression in each group of cells detected by Western blot. The data was expressed as means ± SD. N=3. Normality was assessed using the Shapiro-Wilk test. For comparisons between two groups, Student’s t-test was used. For multiple groups, one-way ANOVA with Tukey’s post-hoc test was applied. ***P<0.01*.

**Figure S14 Role of ALKBH5 in Regulating the G3BP1-YBX1-p53 Axis in the DIR Model**

In the DIR mouse model, we specifically overexpressed ALKBH5 using adeno-associated virus (AAV) technology. Western blot was then used to detect the expression levels of G3BP1, YBX1, and p53 proteins in myocardial tissues of mice across different treatment groups. As shown in Fig. S14B, it was showed that ALKBH5 overexpression significantly increased the protein levels of G3BP1, YBX1, and p53 in myocardial tissues of DIR model rats. Additionally, MeRIP-qPCR was employed to detect the m^6^A levels of G3BP1, YBX1, and p53. As shown in Fig. S14A, Compared with the NS group, the m^6^A levels of G3BP1, YBX1, and p53 were significantly decreased in the DIR group; however, ALKBH5 overexpression only significantly increased the m^6^A level of G3BP1, with no obvious effect on the m^6^A levels of YBX1 and p53. Combined with the results from MeRIP-seq and RNA-seq in Figure 4, which showed that ALKBH5 does not affect the RNA methylation levels of YBX1 and p53, we conclude that ALKBH5 may indirectly regulate the G3BP1-YBX1-p53 axis by influencing the m^6^A level of G3BP1.


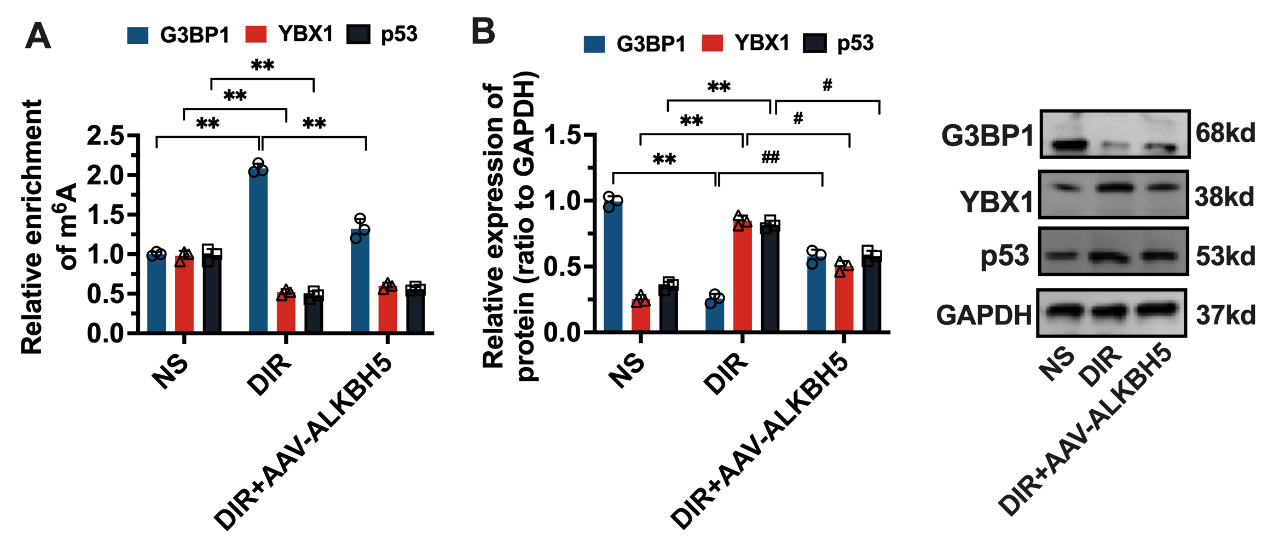


**Figure S14 legend** Role of ALKBH5 in Regulating the G3BP1-YBX1-p53 Axis in the DIR Model. (A) MeRIP-qPCR was employed to detect the m^6^A levels of G3BP1, YBX1, and p53. (B) The protein levels of G3BP1, YBX1, and p53 were detected by western blot. The data was expressed as means ± SD. N=3. Normality was assessed using the Shapiro-Wilk test. For comparisons between two groups, Student’s t-test was used. For multiple groups, one-way ANOVA with Tukey’s post-hoc test was applied. ***P<0.01*. #*P<0.05*. ##*P<0.01*.

**Figure S15** **Role of ALKBH5 in Regulating the G3BP1-YBX1-p53 Axis and Ferroptosis in** **Cardiomyocyte and Fibroblast**

As shown in Fig. S15A, we extracted neonatal rat cardiac fibroblasts (NRCFs) from primary rat cardiomyocytes and detected the expression of the vimentin protein in NRCFs via immunofluorescence. The vimentin protein was expressed in all the extracted cells. As shown in Fig. S15B-C, compared with NMVCs, the mRNA and protein levels of ALKBH5 expression were slightly lower in NRCFs (*P<0.05*).

As shown in Fig. S15D-E, we first verified the effect of lentivirus transfection on ALKBH5 expression in NRCFs cells, and ALKBH5 mRNA and protein levels were not significantly different between the LV-Ctrl group and the NC group. After LV-ALKBH5 treatment, the ALKBH5 mRNA and protein levels were significantly increased (*P<0.01 or 0.05*). The constructed lentivirus can successfully overexpress the ALKBH5 molecule, which can be used for subsequent experiments.

As shown in Fig. S15G–L, compared with those in the NC group, the levels of Fe^2+^, ROS, MDA, 4-HNE, p53^N/T^, ACSL4 mRNA, and YBX1 protein levels in the HH/R group of NMVCs and NRCFs cells were significantly greater (*P*<0.01). GPX4 mRNA and G3BP1, SLC7A11, GPX4 protein levels were significantly lower (*P*<0.01). Compared with those in the HH/R group, the Fe^2+^, ROS, MDA, 4-HNE, p53^N/T^, ACSL4 mRNA, and YBX1 protein levels in the LV-ALKBH5 group were significantly lower (*P<0.01 or 0.05*), and the GPX4 mRNA and G3BP1, SLC7A11, GPX4 protein levels were significantly greater (*P<0.01 or 0.05*) in NMVCs and NRCFs cells. Therefore, the changes of above markers in NRCFs cells were consistent with those in NMVCs cells. The results showed that ALKBH5 overexpression improved the G3BP1-YBX1-p53 axis and ferroptosis pathway in both NMVCs and NRCFs cell models, with more pronounced effects in NMVCs than in NRCFs.


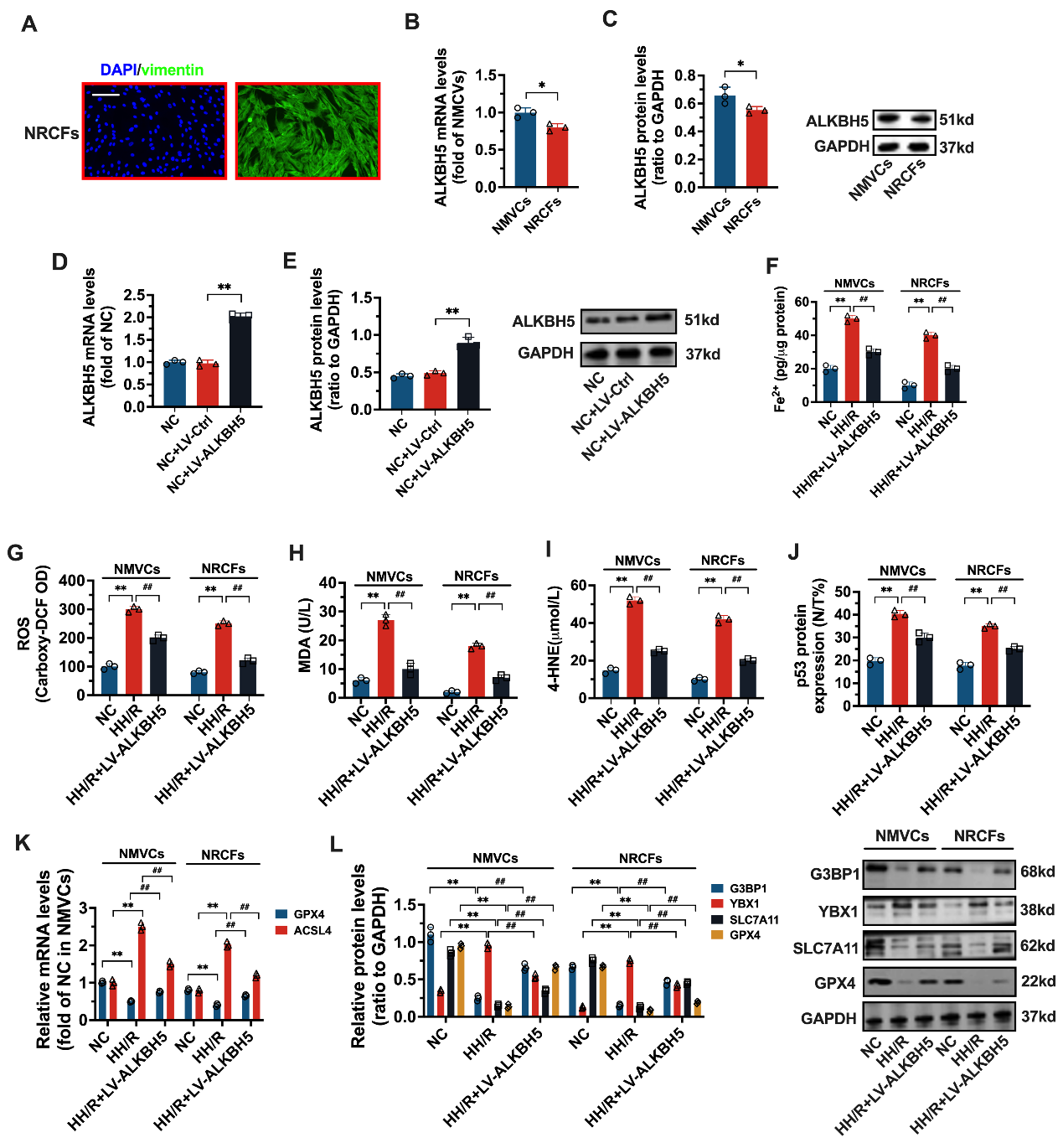


**Figure S15 legend** Role of ALKBH5 in regulating the G3BP1-YBX1-p53 axis and ferroptosis in cardiomyocyte and fibroblast. (A) Immunofluorescence detection of vimentin protein in NRCFs of primary rat cardiomyocytes. (B-C) The mRNA and protein levels of ALKBH5 in NRCFs cells were detected by RT-PCR and western blot. (F-J) The levels Fe^2+^, ROS, 4-HNE, and p53^N/T^ were detected by kit. (K) The mRNA levels of GPX4 and ACSL4 were detected by RT-PCR. (L) The protein levels of G3BP1, YBX1, SLC7A11, and GPX4 were detected by western blot. The data was expressed as means ± SD. N=3. Normality was assessed using the Shapiro-Wilk test. For comparisons between two groups, Student’s t-test was used. For multiple groups, one-way ANOVA with Tukey’s post-hoc test was applied. ***P<0.01*. #*P<0.05*. ##*P<0.01*. The scale bars in Figure A represent 100μm.

**Figure S16** **Diabetes-specific factors influence the mechanisms of myocardial I/R injury, ALKBH5-G3BP1-YBX1/p53 axis and ferroptosis**

As shown in Fig. S16A-H, compared with I/R group, the levels of IA/AAR, Fe^2+^, ROS, MDA, 4-HNE, p53^N/T^, ACSL4 mRNA, and YBX1 protein levels in the DIR group were significantly elevated (*P<0.01*), and the GPX4 mRNA and ALKBH5, G3BP1, SLC7A11, GPX4 protein levels were significantly lower (*P<0.01*).


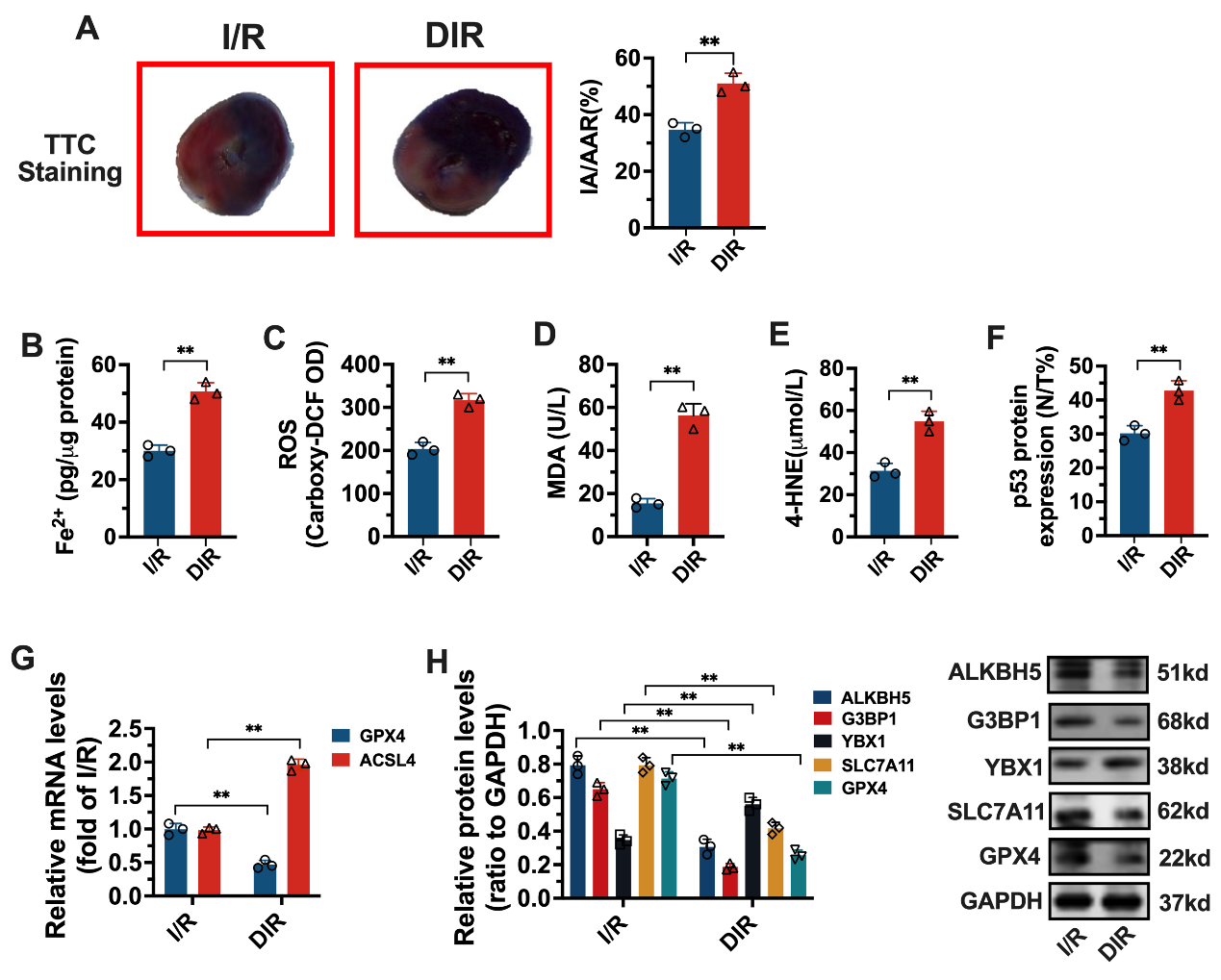


**Figure S16 legend** Diabetes-specific factors influence the mechanisms of myocardial I/R injury, ALKBH5-G3BP1-YBX1/p53 axis and ferroptosis. (A) Triphenyltetrazolium Chloride (TTC) staining (left) of heart sections and calculation of Infarct Area/Affected Area Ratio (IA/AAR) (right) in different groups 24 h post-reperfusion. (B-F) The levels Fe^2+^, ROS, 4-HNE, and p53^N/T^ were detected by kit. (G) The mRNA levels of GPX4 and ACSL4 were detected by RT-PCR. (H) The protein levels of ALKBH5, G3BP1, YBX1, SLC7A11, and GPX4 were detected by western blot. The data was expressed as means ± SD. N=3. Normality was confirmed using the Shapiro-Wilk test. Two-group comparisons were analyzed by Student’s t-test. ***P<0.01*. #*P<0.05*. ##*P<0.01*.

**Supplementary Table S1 Primer sequences for each molecule**

| **Gene** | **Forward primer (5`-3`)** | **Reverse primer (5`-3`)** |
| --- | --- | --- |
| **ALKBH5** | TTCTTTAGCGACTCGGCACTTT | CCTTGCGGTGGGACCTTTT |
| **GPX4** | GGGGACAAAGAGCCGGTAG | GGTTACTGGGACCTAGGGGA |
| **SLC7A11** | ATACGCTGAGTGTGGTTTGC | CTTCATCCACTTCCACAGCG |
| **G3BP1** | GGAAGAGATGGTGGGAAGCAGTTG | GCTCACAGGCACTCACAGGAAC |
| **GAPDH** | CCGCATCTTCTTGTGCAGTG | CGATACGGCCAAATCCGTTC |

**Supplementary Table S2** **Baseline characteristics of every participant**

| **Subject** | **Diagnosis** | **Age**  **(year)** | **Gender** | **Glu**  **(mmol/L)** | **NT-proBNP**  **(pg/mL)** | **cTnI**  **(ng/mL)** | **HbA1**  **(%)** | **TC**  **(mmol/L)** | **LVEF**  **(%)** |
| --- | --- | --- | --- | --- | --- | --- | --- | --- | --- |
| 1 | Donor | 37 | Female | 4.2 | 17 | 1.5 | 3.9 | 3.6 | 60 |
| 2 | Donor | 55 | Male | 5.5 | 67 | 6.1 | 4.2 | 4.2 | 62 |
| 3 | Donor | 28 | Male | 6.0 | 50 | 14.7 | 4.1 | 4.9 | 58 |
| 4 | Donor | 50 | Male | 5.2 | 70 | 4.8 | 4.9 | 5.5 | 59 |
| 5 | Donor | 50 | Male | 6.1 | 49 | 13.6 | 4.5 | 3.5 | 60 |
| 6 | Donor | 45 | Female | 5.9 | 85 | 7.8 | 5.1 | 3.8 | 62 |
| 7 | Donor | 55 | Male | 8.2 | 132 | 15.0 | 6.0 | 3.9 | 52 |
| 8 | Donor | 52 | Male | 5.0 | 118 | 11.6 | 5.5 | 5.1 | 55 |
| 9 | DIC | 63 | Male | 8.6 | 2030 | 97.8 | 9.7 | 5.3 | 38 |
| 10 | DIC | 58 | Male | 8.3 | 1465 | 315.6 | 6.9 | 6.7 | 45 |
| 11 | DIC | 59 | Male | 6.7 | 237 | 123.4 | 5.5 | 5.0 | 50 |
| 12 | DIC | 61 | Male | 7.3 | 218 | 205.4 | 8.3 | 6.0 | 46 |
| 13 | DIC | 56 | Male | 8.1 | 329 | 17.4 | 9.6 | 6.7 | 55 |
| 14 | DIC | 60 | Female | 12.3 | 1242 | 162.1 | 6.5 | 6.9 | 49 |
| 15 | DIC | 59 | Female | 7.2 | 1473 | 106.9 | 5.9 | 7.5 | 45 |
| 16 | DIC | 65 | Male | 15.1 | 4892 | 117 | 6.3 | 5.4 | 46 |
| 17 | DIC | 59 | Male | 7.2 | 1281 | 641.51 | 5.5 | 4.9 | 51 |
| 18 | DIC | 62 | Male | 6.2 | 621 | 22.8 | 6.2 | 5.0 | 57 |
| 19 | DIC | 63 | Male | 5.1 | 5450 | 87.02 | 5.9 | 4.8 | 53 |
| 20 | DIC | 56 | Male | 7.0 | 1837 | 91.2 | 5.6 | 4.7 | 47 |
| 21 | DIC | 57 | Male | 6.5 | 539 | 19.1 | 4.7 | 5.2 | 45 |
| 22 | DIC | 59 | Female | 5.6 | 75 | 28 | 5.6 | 4.3 | 40 |
| 23 | DIC | 59 | Female | 7.3 | 55 | 8.9 | 7.0 | 5.1 | 49 |

**Supplementary Table S3 Statistical data on Baseline characteristics of the participants**

|  | **Donor (n=8)** | **DIC (n=15)** |
| --- | --- | --- |
| **Age (year)** | 46.5±9.52 | 59.73±2.63 |
| **Sex(M/F)** | 6/2 | 11/4 |
| **Glu (mmol/L)** | 5.76±1.17 | 7.9±2.59* |
| **NT-proBNP (pg/mL)** | 73.50±37.67 | 1449.6±1647.63** |
| **cTnI (ng/L)** | 9.38±5.06 | 136.28±162.32* |
| **HbA1 (%)** | 4.78±0.73 | 6.61±1.48** |
| **TC (mmol/L)** | 4.31±0.75 | 5.57±0.95** |
| **LVEF (%)** | 58.50±3.46 | 47.73±5.13** |

**Supplementary Table S4 Comparative Summary for therapeutics**

| **Parameter** | **ALKBH5-G3BP1 Axis** | **Ferroptosis Inhibitors** | **Antioxidants** |
| --- | --- | --- | --- |
| **Efficacy (Infarct Size)** | ↓42% (animal model) | ↓30–40% (animal models) | ↓<20% (animal/models) |
| **Target Specificity** | Epigenetic regulation of ferroptosis | Direct lipid peroxidation inhibition | Broad ROS scavenging |
| **Safety Profile** | No acute toxicity (rodents) | Immunosuppression, off-target effects | Low toxicity, but limited efficacy |
| **Duration of Action** | 4–6 weeks (AAV) | Short half-life (<24 hours) | Transient (<48 hours) |
| **Translation Status** | Preclinical | Phase II trials (e.g., Liproxstatin-1) | Clinically approved |

**Supplementary Descriptions**:

1. **The KEGG and GO pathway analysis for RNA-seq and MeRIP-seq**

The KEGG and GO pathway analysis for RNA-seq were in MAPK signaling pathway, Pathways in cancer, Cytokine-cytokine receptor interaction, Toxoplasmosis, Cell cycle, Malaria, p53 signaling pathway, Protein digestion and absorption, NOD-like receptor signaling pathway, and African trypanosomiasis. The KEGG and GO pathway analysis for MERIP-seq were Leukocyte transendothelial migration, Growth hormone synthesis, secretion and action, Vasopressin-regulated water reabsorption, Phospholipase D signaling pathway, Inositol phosphate metabolism, Bacterial invasion of epithelial cells, Viral carcinogenesis, cAMP signaling pathway, Salmonella infection, Phosphatidylinositol signaling system, NF-kappa B signaling pathway, Endocytosis, HIF-1 signaling pathway, Estrogen signaling pathway, Yersinia infection, MicroRNAs in cancer, RNA transport and Tyrosine metabolism.

Based on the KEGG and GO pathway analyses of RNA-seq and MeRIP-seq data, several critical pathways emerge that directly align with the study’s focus on ferroptosis and diabetic myocardial ischemia-reperfusion (I/R) injury. While the initial analysis broadly categorized pathways, a refined interpretation highlights key signaling axes that mechanistically link ALKBH5-mediated m6A modification, stress granule (SGs) dynamics, and ferroptosis regulation.

The RNA-seq data identified MAPK signaling, p53 signaling, and cytokine-cytokine receptor interaction as top pathways. These pathways are intrinsically tied to ferroptosis: (1) MAPK signaling (e.g., JNK/p38) modulates oxidative stress and lipid peroxidation, both hallmarks of ferroptosis; (2) p53 directly regulates ferroptosis via transcriptional control of SLC7A11 and other redox-sensitive genes; and (3) cytokine signaling (e.g., TNF-α/IL-6) exacerbates inflammatory injury, synergizing with ferroptosis in diabetic myocardium. Notably, our data show that ALKBH5 overexpression suppresses p53 nuclear translocation (via G3BP1-YBX1 interaction), thereby rescuing SLC7A11 expression and glutathione synthesis—a mechanism directly intersecting with the p53 pathway.

The MeRIP-seq analysis further revealed enrichment in NF-κB signaling, HIF-1 signaling, and phospholipase D (PLD) pathways. These pathways contextualize the interplay between metabolic stress and epigenetic regulation: (1) NF-κB activation in diabetes amplifies inflammatory damage and mitochondrial ROS production, priming cells for ferroptosis; (2) HIF-1α stabilization under hypoxia reprograms iron metabolism and lipid peroxidation; and (3) PLD signaling influences membrane phospholipid remodeling, a critical step in ferroptotic membrane rupture. Importantly, ALKBH5’s role in reducing m6A levels on G3BP1 mRNA stabilizes SGs, which sequester pro-ferroptotic mRNAs (e.g., p53, YBX1) and disrupt NF-κB/HIF-1-driven transcriptional cascades. This SGs-mediated buffering aligns with the observed reduction in mitochondrial ROS and lipid peroxidation markers (MDA, 4-HNE) upon ALKBH5 overexpression.

The intersection of RNA-seq and MeRIP-seq data underscores leukocyte transendothelial migration and viral carcinogenesis pathways. While these may appear tangential, they reflect the systemic inflammatory milieu of diabetes. Leukocyte infiltration exacerbates myocardial oxidative damage, while viral carcinogenesis-related genes (e.g., TLR4, MYC) overlap with ferroptosis regulators (e.g., SLC7A11). The shared emphasis on redox imbalance and inflammatory signaling reinforces the centrality of ferroptosis in diabetic I/R injury.

Strikingly, the m6A-dependent regulation of G3BP1 bridges these pathways. G3BP1’s 3′UTR m6A sites (142/173) govern its mRNA stability, and ALKBH5-mediated demethylation enhances G3BP1 expression. This stabilizes SGs, which physically sequester YBX1/p53 in the cytoplasm, blocking their nuclear pro-ferroptotic functions. This mechanism directly intersects with the p53 and MAPK pathways while countering NF-κB/HIF-1-driven inflammation. Thus, the existing KEGG/GO results inherently highlight pathways central to ferroptosis, albeit requiring contextual integration with the ALKBH5-G3BP1 axis.

In summary, the current pathway analyses provide a robust foundation to prioritize p53 signaling, MAPK/NF-κB inflammation, and SG-mediated RNA stability as critical axes. Future discussions can deepen mechanistic links without additional experiments, emphasizing how ALKBH5’s epigenetic regulation of G3BP1 disrupts these pathways to mitigate ferroptosis.

**References**

[1] Y. Yang, Y. Zhang, J. Yang, M. Zhang, T. Tian, Y. Jiang, X. Liu, G. Xue, X. Li, X. Zhang, S. Li, X. Huang, Z. Li, Y. Guo, L. Zhao, H. Bao, Z. Zhou, J. Song, G. Yang, L. Xuan, H. Shan, Z. Zhang, Y. Lu, B. Yang, Z. Pan, Interdependent Nuclear Co-Trafficking of ASPP1 and p53 Aggravates Cardiac Ischemia/Reperfusion Injury, Circ Res 132(2) (2023) 208-222.

[2] J.P. Kaltenbach, R.B. Jennings, Metabolism of ischemic cardiac muscle, Circ Res 8 (1960) 207-13.

[3] O. Akande, Q. Chen, S. Toldo, E.J. Lesnefsky, M. Quader, Ischemia and reperfusion injury to mitochondria and cardiac function in donation after circulatory death hearts- an experimental study, PLoS One 15(12) (2020) e0243504.

[4] Y. Zhang, X. Li, Y. Dai, Y. Han, X. Wei, G. Wei, W. Chen, S. Kong, Y. He, H. Liu, N. Ma, J. Bin, N. Tan, P. He, Y. Liu, Neutrophil N1 polarization induced by cardiomyocyte-derived extracellular vesicle miR-9-5p aggravates myocardial ischemia/reperfusion injury, J Nanobiotechnology 22(1) (2024) 632.

[5] K. Venkatachalam, S.D. Prabhu, V.S. Reddy, W.H. Boylston, A.J. Valente, B. Chandrasekar, Neutralization of interleukin-18 ameliorates ischemia/reperfusion-induced myocardial injury, J Biol Chem 284(12) (2009) 7853-65.

[6] S.T. Chang, C.M. Chu, C.S. Cheng, K.L. Pan, Y.S. Lin, C.M. Chung, M.S. Lin, Effect of the Ligation and Reperfusion Timeframe on Maximal Ischemia-Reperfusion Injury in Diverse Rat Models, Acta Cardiol Sin 39(1) (2023) 135-143.
